# Genomic characterisation of perinatal Western Australian *Streptococcus agalactiae* isolates

**DOI:** 10.1371/journal.pone.0223256

**Published:** 2019-10-02

**Authors:** Lucy L. Furfaro, Barbara J. Chang, Charlene M. Kahler, Matthew S. Payne

**Affiliations:** 1 The School of Medicine, Division of Obstetrics and Gynaecology, The University of Western Australia, Perth, Western Australia, Australia; 2 The School of Biomedical Sciences, The Marshall Centre for Infectious Diseases Research and Training, The University of Western Australia, Perth, Western Australia, Australia; Institut National de la Recherche Agronomique, FRANCE

## Abstract

As a leading cause of neonatal sepsis, *Streptococcus agalactiae*, commonly known as Group B Streptococcus, is a major neonatal pathogen. Current global screening practices employ risk- or culture-based protocols for detection of these organisms. In Western Australia (WA), universal culture-based screening is provided, with subsequent intrapartum antibiotic prophylaxis for all *S*. *agalactiae*-positive women during labour. Widespread antibiotic exposure is not ideal and this is one of the factors driving development of vaccines against *S*. *agalactiae*. Vaccine candidates have focused on the capsule, surface proteins and pilus types, however, capsule serotypes are known to vary geographically. The aim of this study was to use genome sequencing to gain an understanding of the circulating genotypes in WA, and to assess variations in the associated gene pools. We sequenced 141 antenatal carriage (vaginal/rectal) isolates and 10 neonatal invasive disease isolates from WA. Based on the global PubMLST database, the 151 strains were characterised into 30 sequence types, with clustering of these mainly into clonal complexes 1, 12, 17, 19 and 23. Of the genes encoding eleven surface proteins that were analysed, the most prevalent were *fbp*, *lmb* and *scpB* which were present in ≥ 98% of isolates. A cluster of non-haemolytic isolates, one of which was a neonatal invasive disease isolate, appeared to lack the entire *cyl* locus. Admixture analysis of population structure revealed evidence of genetic transfer among the WA isolates across structural groups. When compared against the PubMLST *S*. *agalactiae* data, WA isolates showed high levels of strain diversity with minimal apparent clustering. This is the first whole genome sequence study of WA *S*. *agalactiae* isolates and also represents the first addition of Australian isolate data to PubMLST. This report provides insight into the distribution and diversity of vaccine targets of *S*. *agalactiae* within Western Australia, indicating that the most appropriate capsular vaccine for this population would be the proposed pentavalent (Cps Ia, Ib, II, III and V) preparation, whilst vaccines targeting surface proteins should ideally utilise Fbp, Lmb and/or ScpB.

## Background

As an opportunistic pathogen, *Streptococcus agalactiae*, commonly known as Group B Streptococcus, is able to reside as a commensal in some individuals and cause serious infection in others; commonly those who are immunocompromised, and typically the elderly or neonates. As the leading cause of neonatal sepsis, *S*. *agalactiae* is targeted in the antenatal and intrapartum period by antibiotic treatment to reduce the risk of transmission from mother to infant and effectively reduce associated disease [1]. Currently, all women in Western Australia (WA) are offered *S*. *agalactiae* testing to assess presence at 35–37 weeks gestation and this involves culture analysis. Approximately 10–30% of women will be carriers of *S*. *agalactiae* and receive antibiotics based on this presence/absence result [2]. This universal screening practice provides a currently under-utilised opportunity to attain a better understanding of circulating *S*. *agalactiae* strains in our population through serotyping and whole genome sequencing of clinical specimens to further refine *S*. *agalactiae* diagnostic and therapeutic approaches.

The recent comprehensive systematic review and meta-analysis by Russell et al. [2] demonstrated the substantial global geographical variation in circulating *S*. *agalactiae* serotypes. For example, serotype III, most commonly associated with invasive disease, had an overall global prevalence rate of 25% of colonised women, yet in Central America and South-Eastern Asia the prevalence rate was less than half this at 11% and 12%, respectively. In addition, Dutra et al. [3] documented regional variation in circulating GBS serotypes in Brazil highlighting the importance of documenting population dynamics at a local level, such as in WA, where we recently described the first data on circulating GBS capsular genotypes [2–4]. However, although knowledge of capsule (CPS) serotype is of significant importance for formulating vaccines that target the *S*. *agalactiae* capsule, with the exception of CPS III, information on serotype is not particularly useful for comparing carriage and invasive disease isolates which are generally found distributed amongst all serotypes [5]. This is further complicated by reports of capsular switching [6–9] and discrepant results between serotyping methods [10,11].

While some serotypes predominate across all countries, variation can impact the potential vaccine coverage within each population. In Japan for example, the globally rarer serotypes such as VI, VIII and incidences of IX, are observed more frequently [12]. Serotype VIII in particular is strongly associated with Japan, with studies reporting high rates in Japanese pregnant women [13]. It is apparent that each region should tailor serotype vaccines to their population to optimise efficacy. The success of this approach will rely on the knowledge of the circulating serotypes within each country.

Beyond serotype description, we have the capability of delving more deeply into the characterisation and epidemiology of *S*. *agalactiae* isolates and a number of molecular approaches have been described [12]. A common molecular identifier is through use of the seven housekeeping genes, *adhP*, *pheS*, *atr*, *glnA*, *sdhA*, *glcK* and *tkt*, described by Jones and colleagues [14]. The multi locus sequence typing (MLST) database, PubMLST, is a valuable public source of *S*. *agalactiae* sequence typing data, however, it currently contains no Australian isolates. MLST profiles were often determined via PCR [14], however, the use of whole genome sequencing (WGS) is becoming more popular. Population analysis allowed by WGS enables the detection of new and emerging clusters of GBS, some of which may represent pathogenic clades, which would, therefore, have implications for public health surveillance. The depth of information that can be afforded by such methods can also clarify the origins of disease. For example, Da Cunha and others [15] used WGS to show that the emergence of *S*. *agalactiae* disease in humans can be traced through genetic lineage as a result of extensive tetracycline use.

The aim of this study was to use WGS to characterise circulating *S*. *agalactiae* strains in WA pregnant women and neonates and to compare these data to those from other geographical areas, in an effort to inform vaccine targets.

## Methods

### Clinical isolates

Antenatal isolates were collected upon written consent as part of a larger cohort study, Predict1000, which was approved by the Women and Newborn Health Service Human Research Ethics Committee (201535EW). This study was conducted at King Edward Memorial Hospital, Perth, the largest Tertiary Obstetrics Hospital in WA, over 2015–2017 [4]. The majority of the participants included in this study resided in the broader Perth and surrounding areas, however, rural participants were also represented in this cohort (n = 4). Specimen collection involved self-collected vaginal and rectal e-swabs (Copan, Italy) from pregnant women at 14–22 weeks gestational age (GA) and again at 33–40 weeks GA. Specimens were processed immediately by culture and also stored at -80°C for molecular analysis. Culture for *S*. *agalactiae* detection involved Strep B Carrot Broth^™^ (Hardy Diagnostics, California, USA) followed by sub-culture onto CHROMagar™ StrepB (CHROMagar, Paris, France), the latter with the ability to detect non-haemolytic *S*. *agalactiae* isolates. All *S*. *agalactiae* positive cultures were confirmed by PCR [16] and stored at -80°C as pure isolates. Of the 151 antenatal isolates, 139 represented isolates from different participants, while 12 isolates represented multiple sampling (different gestation or sample site) of 5 participants. Ten archived invasive disease isolates collected from neonatal blood cultures at King Edward Memorial Hospital over 2012–2014 were also included in analyses, and these were cultured from pure stocks.

### DNA extraction

DNA from pure *S*. *agalactiae* isolates was extracted using the QIAGEN MagAttract Microbial DNA Extraction Kit (QIAGEN) as per manufacturer’s instructions using the KingFisher Duo (ThermoFisher Scientific) automated platform.

### Whole genome sequencing

Extracted DNA was quantified on a Qubit^TM^ fluorometer (Life Technologies) with a dsDNA broad range kit (Life Technologies), and 1.0 ng of DNA was used in the Illumina Nextera XT library preparation protocol as per manufacturer’s instructions (Illumina). The genomic DNA was tagmented, indexed by PCR and purified using AMPure XP beads. Library pooling was performed after quantification of the library size using the LabChip^®^ GXII. The pooled DNA library (12 pM) from all 151 *S*. *agalactiae* strains was sequenced on an Illumina Nextseq using a paired-end 300 bp read workflow.

### Sequence analysis

DNA sequences were analysed using the Short Read Sequence Typing for Bacterial Pathogens (SRST2) program [17] for the detection of the Multi-Locus Sequence Typing (MLST) loci [14]. Alleles were assigned integers using the *S*. *agalactiae* database within PubMLST (http://pubmlst.org/sagalactiae/) [18]. The seven housekeeping loci used for MLST were *adhP*, *pheS*, *atr*, *glnA*, *sdhA*, *glcK* and *tkt* [14]. Assembly was performed using SPAdes (version 3.10.3) [19] followed by the PubMLST database automated pipeline for annotation of genes, in addition to the online tool Genome Comparator [18]. All assembled genomes were analysed using QUAST genome assembly evaluation tool [20] to ensure quality (S2 Table). The capsular genotypes were determined through analyses of the capsular locus as previously described [21]. Additionally, SRST2 [17] was used to identify partial genes which were not annotated by the automated pipeline (alleles: *rib* = 1, *bac* = 1–89, *bca* = 1, *alp2* = 1–2, *alp3* = 1–6 and *alp4* = AJ488912.1). The presence of *alp* genes encoding a family of surface protein antigens was confirmed by multiplex PCR, as described previously [22] and confirmed with Sanger sequencing. Those that were not confirmed by PCR were manually curated to ensure flanking regions of the gene were adjacent or that SRST2 did not detect partial genes. Population structure was analysed using BAPS (version 6) [23] with hierarchical clustering via HierBAPS admixture analysis [24].

### Representative global database

The PubMLST *S*. *agalactiae* database (http://pubmlst.org/sagalactiae/) [18] contained 4,214 *S*. *agalactiae* publicly available submissions; of these, a total of 2,801 isolate records contained >1500 tagged loci, indicative of genomic sequence availability. These were exported and assessed to include all variations available for continent, country, ST, source, disease and year. The resulting dataset of 1378 isolates [25–29] was used as a global representative for further analysis and comparison with our Australian isolates (S3 Table). The genome comparator tool was used to align all loci, followed by neighbour-joining tree analysis using MEGA (version 7) [30] with 500 boot-straps and visualised using the Interactive Tree of Life online software (iTOL version 3) [31].

## Results

### Population structure

**Fig 1:**
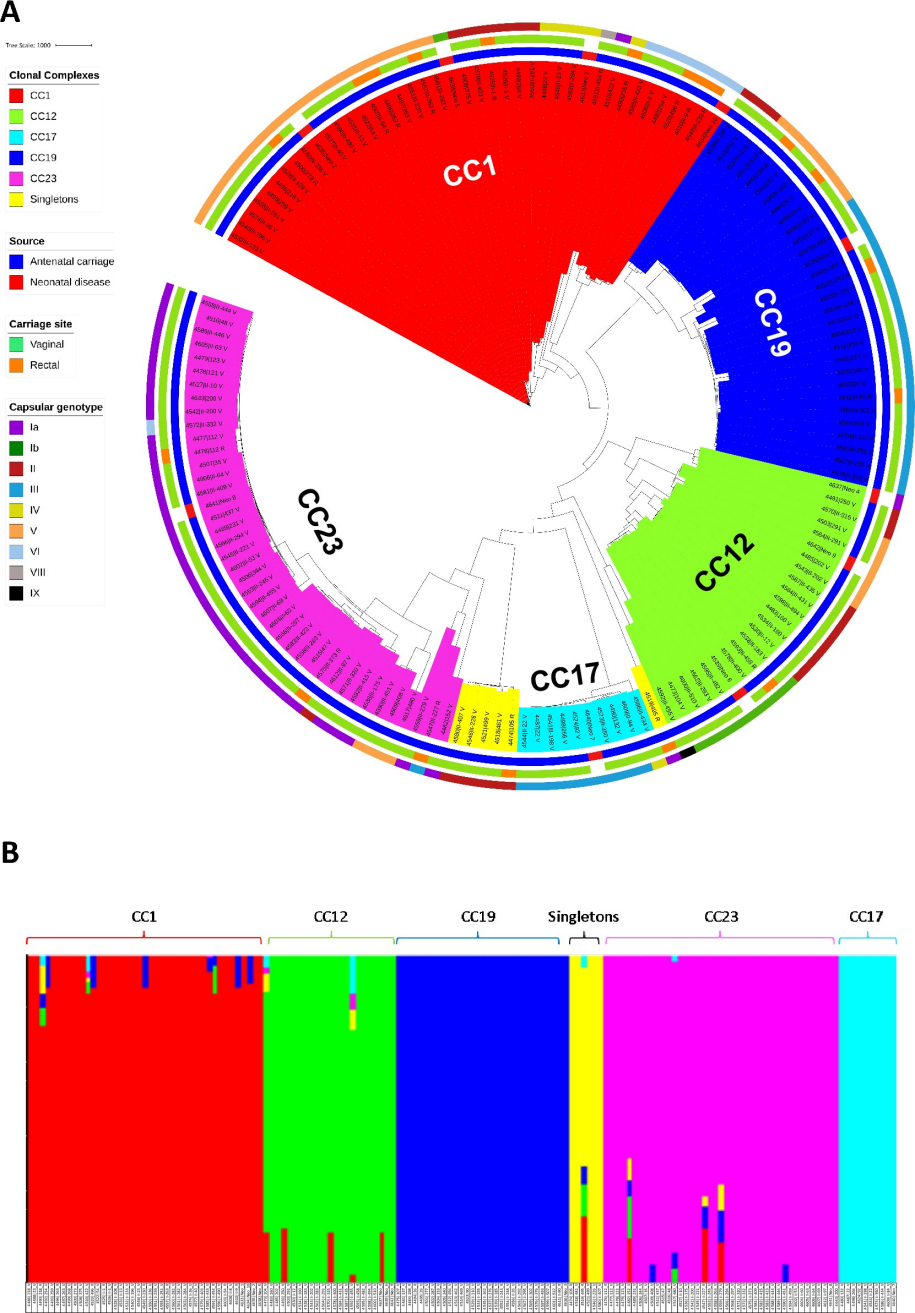
**(A) The core genome maximum likelihood phylogeny of 151 clinical *Streptococcus agalactiae* isolates collected from Western Australian pregnant women (n = 141) and neonates (n = 10).** Clonal complexes are highlighted within the tree. The first colour strip indicates antenatal carriage (blue) and neonatal invasive disease (red) isolate origins. The central colour strip indicates carriage site for the antenatal isolates, either vaginal (green) or rectal (orange) and the outer colour strip indicates the capsular genotype (Ia–IX). **(B) Admixture analysis of population structure of the same *S*. *agalactiae* isolates as defined by BAPS.** Isolates are displayed as vertical coloured bars, with colours indicative of the proportion of admixture that has occurred between the isolate and other structural groups.

The majority of the clinical isolates collected from WA were observed to cluster into five clonal complexes (CCs), CC1, CC12 (also known as CC10), CC17, CC19 and CC23 (Fig 1A), however, a small cluster (n = 5) of ST22 isolates (which may be considered CC17) and a single ST248 isolate were also observed. Neonatal invasive disease-causing isolates were distributed relatively evenly among the antenatal carriage isolates across each CC. The phylogenetic tree revealed diversity within the CC1 lineage with various branches and seven (Ia, Ib, II, IV, V, VI and VIII) associated capsular genotypes (n = 41 isolates). In contrast, CC17 appeared largely clonal and consisted of ST17 (all CPS III, n = 9) and a single ST291 (CPS IV) isolate. Similarly, CC23 also exhibited minimal branching in the WA population, comprising four capsular genotypes (Ia, III, V and VI) indicative of recent clonal expansion (n = 41). BAPS analysis identified six subgroups within the WA *S*. *agalactiae* population in accordance with the CCs and phylogenetic analysis. Multiple isolates were sequenced from 13 patients and included different sites (vaginal and rectal) and/or different gestational ages during pregnancy. Paired isolates clustered together as identical isolates, except for two cases. The first case included vaginal and rectal specimens from a mother at both sampling gestations (≤22 weeks and ≥33 weeks) where three of the four *S*. *agalactiae* isolates were identical and within CC1 (263_V, 263_R and II-263_R), while the remaining isolate collected from the vagina (II-263_V) was distinct and clustered within CC23. Another case included specimens from different sites of the same mother (198_R and II-198_V) at different gestational ages which were genetically distinct (S1 Table).

### Admixture analysis

Admixture analysis exposed the extent of genetic exchange evident in this population and identified various donor subgroups (Fig 1B). CC17 and CC19 showed no evidence of recombination within this population and appeared only as genetic donors, particularly CC19 with CC1 and CC23. CC12 appeared to have obtained the majority of genetic information from CC1. The singleton cluster appeared uniform (all ST22 isolates), except for a single ST248 isolate, which branched outside the ST22 cluster and contained recombination originating from CC1, CC12 and CC19.

### Sequence types

A total of 150/151 isolates were represented by 30 currently described STs. A novel MLST profile was identified for an additional isolate with the allele combination of 5, 4, 6, 1, 2, 1 and 2 for *adhP*, *pheS*, *atr*, *glnA*, *sdhA*, *glcK* and *tkt*, respectively. Three STs (ST1, 23 and 19) accounted for >50% of the isolates. Comparison with the PubMLST database revealed several STs that had hypothetical allelic profiles and had been assigned ST numbers accordingly, but where no representative clinical isolates had currently been described (ST15, ST248, ST414 and ST509). In addition, six STs including ST861, ST1167, ST529, ST569, ST585 and ST890 were identified where only one isolate had been submitted to the database.

### Virulence genes

Several virulence genes were examined across this cohort including those encoding capsules, surface proteins, pilus islands, haemolysins and hyaluronate lyase (Table 1). All capsular genotypes were represented in this study, except for CPS VII. Overall, capsular genotypes Ia, V, III and II predominated in this cross-sectional population. Neonatal disease isolates were only represented by capsular genotypes Ia–VI. Eleven surface protein genes were assessed, with fibrinogen-binding protein (SAG0152), laminin-binding protein (SAG01234), and serine peptidase (SAG1236) detected in ≥ 98% of all sequences. To a lesser extent, *bac* (SAG2195) was detected in 15.7% and *rib* (SAG0433) in 27.8% of isolates. The majority of isolates contained pilus islands 1 and 2a (1/2a) in combination followed by 2a, 1/2b and 2b. The *cyl* genes that form the haemolysin locus were detected in the majority of isolates, however, we were unable to detect any genes within the entire locus for six isolates, including pairs 291_V, II-291_V, 202_V and II-202_V, which comprised capsular genotype V, ST41 isolates within CC12. The other two isolates included ST585 neonatal invasive isolate (Neo_9, also capsular genotype V) within CC12 and ST22 carriage isolate (105_R, capsular genotype II). In addition, seven other strains lacked detection of different *cyl* genes within the locus including II-493_V, II-302_V, 469_V (capsular genotype III, ST19), II-431_V, Neo_6 (capsular genotype II and Ib, respectively, ST12), Neo_2 (capsular genotype V, ST1) and 152_V (capsular genotype Ia, ST23). Additionally, 80% of the isolates possessed detectable *hlyB* gene sequences (SAG1197) encoding hyaluronate lyase; of those lacking *hlyB*, 20/30 were capsular genotype III, CC19 isolates.

**Table 1:** Summary of the presence of virulence genes including the capsule, surface proteins, pilus islands, haemolysin and hyaluronate lyase within the antenatal carriage and neonatal invasive disease isolates collected from Western Australia.

	Antenatal n (%)		Neonatal n (%)
Capsular genotype	Vaginal	Rectal	
Ia	34 (28.1)	3 (15)	1 (10)
Ib	6 (5)	1 (5)	2 (20)
II	20 (16.5)	2 (10)	1 (10)
III	25 (20.7)	3 (15)	2 (20)
IV	4 (3.3)	1 (5)	1 (10)
V	27 (22.3)	6 (30)	2 (20)
VI	4 (3.3)	3 (15)	1 (10)
VII	0	0	0
VIII	0	1 (5)	0
IX	1 (0.8)	0	0
**Surface Proteins**			
SAG0433 (*rib*)	35 (28.9)	4 (20)	3 (30)
SAG0832 (*hyp*)	59 (48.8)	13 (65)	8 (80)
SAG1052 (*fbp*)	118 (97.5)	20 (100)	10 (100)
SAG1234 (*lmb*)	120 (99.2)	19 (95)	10 (100)
SAG1236 (*scpB*)	120 (99.2)	19 (95)	10 (100)
SAG2195 (*bac*)	19 (15.7)	1 (5)	3 (30)
SAG2196 (*bca*)	25 (20.7)	5 (25)	3 (30)
AH013348 (*alp1*)	37 (30.6)	5 (25)	2 (20)
SAG2197 (*alp2*)	2 (1.6)	0 (0)	0
SAG2198 (*alp3*)	20 (16.5)	7 (35)	2 (20)
AJ488912.1 (*alp4*)	0 (0)	0 (0)	0 (0)
**Pilus Islands**			
2a	46 (38)	4 (20)	1 (10)
1/2a	65 (53.7)	14 (70)	8 (80)
2b	0	1 (5)	1 (10)
1/2b	10 (8.3)	1 (5)	0
**Haemolysin Locus**			
SAG0663 (*cylD*)	116 (95.9)	19 (95)	9 (90)
SAG0664 (*cylG*)	116 (95.9)	19 (95)	8 (80)
SAG0665	117 (96.7)	19 (95)	9 (90)
SAG0666 (*cylZ*)	117 (96.7)	19 (95)	9 (90)
SAG0667 (*cylA*)	116 (95.9)	19 (95)	9 (90)
SAG0668 (*cylB*)	117 (96.7)	19 (95)	9 (90)
SAG0669 (*cylE*)	116 (95.9)	19 (95)	8 (80)
SAG0670 (*cylF*)	116 (95.9)	19 (95)	9 (90)
SAG0671 (*cylI*)	116 (95.9)	19 (95)	9 (90)
SAG0672 (*cylJ*)	117 (96.7)	19 (95)	9 (90)
SAG0673 (*cylK*)	115 (95)	19 (95)	9 (90)
**Hyaluronate lyase**			
SAG1197 (*hlyB*)	95 (78.5)	18 (90)	8 (80)
**Total (n)**	**121**	**20**	**10**

### Global comparison

**Fig 2:**
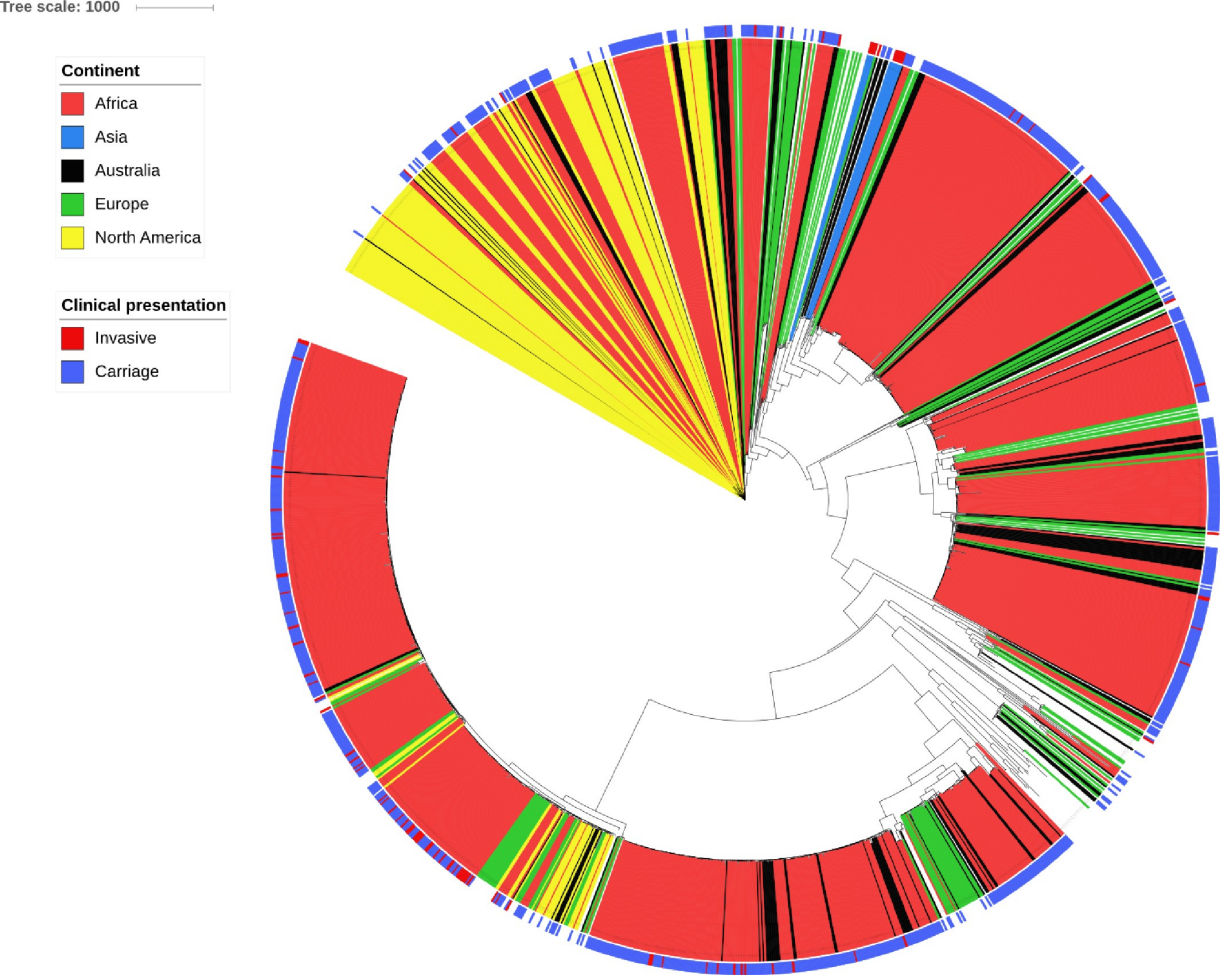
Maximum likelihood phylogeny tree comparing a global dataset representative of the diversity available in PubMLST to Western Australian isolates. The clade colouring is indicative of the continent of isolate origin and the outer colour strip indicates the clinical presentation of invasive disease or carriage.

In order to compare the genetic profiles of Australian isolates with those from other international cohort studies, WGS data contained within the PubMLST database was accessed. A representative global database was selected as described above and consisted of 1,378 isolates containing whole genome sequence data representative of the diversity available from the PubMLST database; these data were then used for comparison with the Australian isolates (Fig 2). A maximum likelihood phylogeny tree constructed from the combined global and Australian isolate data was dominated by African isolates (a notable bias as these represent the majority of the *S*. *agalactiae* isolates in the PubMLST database). Although cladal structures were discernible, isolates from Africa, Europe and Australian origin were distributed amongst these clades suggesting there was no linkage with geographic isolation of the strains. In contrast, North American isolates clustered into a limited number of cladal groups (Fig 2). There was no apparent clustering of invasive disease isolates within any of the global data, with distribution of these amongst carriage isolates being commonplace.

## Discussion

Whole genome sequencing of WA *S*. *agalactiae* isolates has revealed a clonal population structure with evidence of genetic exchange between structural groups. The five main CCs identified here included CC1, CC12, CC17, CC19 and CC23. The ten neonatal disease isolates included in this analysis showed no evidence of clustering based on invasive phenotype, rather these isolates were distributed amongst carriage isolates of all CCs. Although the small sample size of neonatal isolates is a limitation, invasive neonatal isolates were distributed across multiple CCs (CC12 = 3/23 (13%); CC17 = 1/10 (10%); CC1 = 4/41 (9.7%); CC19 = 1/30 (3.3%) and 1/41 (2.4%)). Further admixture analysis of the population structure provided evidence of genetic exchange across all structural groups with CC1, CC12 and CC23 being the most diverse of these groups. This diversity was also evident in the capsular genotypes within CC1 (Ia, Ib, II, IV, V, VI and VIII), CC12 (Ia, Ib, II, V and IX) and CC23 (Ia, II, III, V and VI). In contrast, here we observed no evidence of genetic recombination within CC17 (III and IV) and CC19 (II, III and V) which contained uniform structural groups, but appeared to act as potential donors of genetic material to the other CCs.

Virulence factors such as capsule and surface proteins are being assessed as vaccination targets, with several vaccines in development [32,33]. It is crucial to understand the population dynamics of these targets to assess the population coverage of the candidate vaccines, in addition to further understanding their association with virulence. Capsular types are well-described among *S*. *agalactiae* studies and our study is consistent with global data that suggest capsular genotypes Ia, Ib, II, III and V predominate [2,34,35]. Type VI is often reported in Asian countries, in particular Japan [13] and Malaysia [36,37], however, it was prevalent in similar proportions to Ib and IV in our West Australian antenatal cohort. Furthermore, type VIII and IX were also described in this population, which have rarely been reported in Australasia, with CPS VIII representing <2% in some studies [38–40], while IX was first described in 2007 [41] and neither VIII nor IX was detected in another Australian study performed in 2015 [42]. The differences in the genetic diversity of these data sets could be a result of introduction and expansion of founder isolates into these regions and diversification over extended periods of time by evolution or continued introductions into the same region [43].

The emergence of type IV has been reported in the literature in the last decade, particularly in the United States [44–50]. The main circulating STs of CPS IV include ST452 in CC23, ST459 and ST196 in CC1 and ST291 in CC17. We found three STs encompassing the CPS IV isolates in our study including ST196 (n = 4), ST414 (n = 1) and ST291 (n = 1). The only invasive disease isolate of this capsular genotype was an ST196 isolate (Neo_1). This has been observed in other studies [44–48] and interestingly, Lyhs et al. found both cattle and human isolates that represent ST196 of capsular genotype IV, however, genomes were unavailable for comparison [51].

All capsular genotype IV isolates were found to cluster within CC1, except for ST291 which represented the only non-ST17 isolate within CC17. Bellais et al. [6] analysed the evolution of this ST which formed from a capsular switch between a hypervirulent strain from ST17 (CC17) and ST459 (CC1, CPS IV) that provided a 35.5 Kb genomic segment comprising the type IV *cps* operon. Florindo and colleagues [52] found that 10% (9/89) of the type IV isolates were represented by ST291 within CC17. Ferrieri et al. [53] described two ST291 isolates in CC17 isolated from adults (pregnant and non-pregnant) that caused invasive disease. Similarly studies in Ireland and Canada observed type IV isolates of ST291 within CC17 also [8,54]. This has been reported as a notable strain for surveillance monitoring and isolate II-434_V is the first description from Australia. This suggests serotype replacement is a possibility and as with any capsule-specific vaccination strategy, lack of coverage may lead to the emergence of previously rare serotypes causing disease after vaccination implementation [55].

Capsule-based vaccination strategies such as the bivalent (II and III), trivalent (Ia, Ib and III) [33] and pentavalent (Ia, Ib, II, III and V) [32] combinations would potentially cover 35.1%, 51% and 89.4% of isolates in the WA cohort, respectively. The remaining capsular genotypes, as identified in this cohort, include CPS IV (4%), VI (5.3%), VIII (0.7%) and IX (0.7%) and would not be covered and as mentioned above could be a concern given emergence and expansion of previously rare capsular genotypes.

In addition to the capsular polysaccharide, surface proteins have also been shown to be immunogenic and potential vaccine candidates. Rib and alpha proteins have shown promise as surface protein targets with respect to low antibody levels and the association with invasive *S*. *agalactiae* infection [56]. While formulations are currently in development, in this WA cohort *rib* and alpha C (*bca*) genes were not the most frequently identified (27.8% and 21.9%, respectively), suggesting that vaccines targeting these antigens would result in minimal coverage if used in WA women. In contrast, genes encoding surface proteins such as laminin-binding protein, fibrinogen-binding protein and serine peptidase were present in ≥98% of isolates and may represent ideal targets for vaccination (if these genes are expressed) with regard to isolate coverage. Lower prevalence was observed with genes *bac*, *alp1*, *alp2* and *alp3* with 15.2%, 29.1%, 1.3% and 19.2%, respectively, and no *alp4* was detected within WA isolate genomes. In a previous study conducted examining a range of reference isolates from the US and New Zealand, in addition to 206 clinical isolates (NZ and Australia), only a single isolate was found to contain the *alp4* surface protein gene (n = 244) which supports our lack of detection [57]. Dangor and colleagues assessed maternal antibody levels against pilus islands, *bibA* and *fbsA* and found a weak relative association between anti-fbsA antibody concentrations and invasive *S*. *agalactiae* disease. These data suggest that these surface proteins may not be ideal candidates for neonatal disease protection [55].

A neonatal strain of interest was isolate ST585 which had no current PubMLST data, and lacked a detectable haemolysin locus, being one of six isolates (from four subjects) that were non-haemolytic. Non-haemolytic isolates of *S*. *agalactiae* are thought to be rare with descriptions of <5% of isolates lacking haemolysin activity [58,59]. With haemolysin recognised as a major virulence factor, non-haemolytic strains are often considered less pathogenic, however, a case of hypervirulence associated with such a strain has been reported [60]. Our data provides further evidence that non-haemolytic *S*. *agalactiae* ST585 isolates are clinically significant, ST585 (Neo_9) having been isolated from a case of neonatal disease (n = 10). The antenatal carriers of non-haemolytic GBS, however, did not contain any indication of disease development either themselves or in their neonate according to medical record data, highlighting that this is not exclusive to invasive or carriage isolates. Screening approaches that omit the identification of non-pigmented and non-haemolytic isolates could limit the accurate detection of pathogenic *S*. *agalactiae* strains. Capsule, surface proteins and haemolysin production are examples of the virulence factors *S*. *agalactiae* use to cause disease and evade immune defences. A range of other virulence factors have also been described in this species [61–63] and we have examined several of relevance to protein vaccine targets, colonisation and persistence, and disease outcomes.

Another virulence factor, hyaluronate lyase, acts by cleaving a major component of connective tissues and enables the organism to spread. We found the *hlyB* gene to be present within 80% of both carriage and invasive disease isolates. Associations have been made between elevated production of this enzyme and neonatal disease within type III isolates [64], however, other studies have observed invasive disease caused by isolates lacking this gene [65].

The pili of *S*. *agalactiae* represent a potential vaccine target, but have also been linked to the colonisation of the genitourinary tract and biofilm formation. Animal models have revealed that deletion of the *pilA* gene results in reduced ability to persist in the vaginal tract of mice [66]. Similarly, biofilm production (suggestive of a persistent coloniser) has been associated with specific pilus types. Rinaudo et al. reported biofilm-forming capacity in isolates that carried pilus 2a and not pilus types 1 and 2b [67]. One of the two isolates we found with pilus island 2b, isolate Neo_7, was an ST17, capsular genotype III neonatal invasive disease isolate. Lazzarin and colleagues showed that ST17 isolates with pilus 2b knockouts were recovered significantly less from blood, lungs and brain tissue in mice, suggesting that this pilus type plays a role in invasion within this ST [68].

More generally, pilus island distribution was assessed among the *S*. *agalactiae* lineages and we found similar prevalence of pilus islands based on CCs [69]. CC23 was composed mainly of 2a with some 1/2a isolates, CC17 was all 1/2b except for a single 2b isolate, CC12 consisted of mainly 1/2a with several 2a isolates and CC19 (separated into CC1 and CC19 in the previous study) contained mainly 1/2a, but also 1/2b and 2a isolates. Springman reported pilus islands 2a > 1/2a > 1/2b prevalence in maternal colonising strains, with no detection of 2b [69]. We detected all pilus islands, however, with differences in prevalence including 1/2a > 2a > 1/2b > 2b among the antenatal carriage isolates. The single 2b isolate was a singleton of ST248 isolated from the rectum and exhibited a transient loss of colonisation over the two collection time points during pregnancy. Neonatal invasive isolates were also characterised by the previous study and had prevalence of 1/2b > 1/2a > 2a, while again, no 2b was detected [69]. Our study found an order of prevalence of 1/2a > 2a > 2b, with no 1/2b detected in the small number of neonatal invasive disease isolates characterised. Interestingly, the neonatal 2b isolate represented one of two isolates in which the 2b pilus island was detected in the entire cohort.

The small sample size of neonatal isolates in comparison to the antenatal isolates represents a limitation of this study; however, we have attempted to adjust for this through comparison to the extensive global data on invasive *S*. *agalactiae* disease. We present genomic analysis of isolates of clinical importance to WA pregnant women and neonates and have shed light on the population structure of this group. This is the initial step toward monitoring *S*. *agalactiae* evolution in our population.

## Conclusion

Current global screening protocols, particularly those culture-based approaches (as implemented in WA) resemble an ideal surveillance tool for *S*. *agalactiae* in individual populations. We have described *S*. *agalactiae* isolate genetic diversity within a WA cohort for the first time, and have added valuable whole genome sequence data to the public database to allow more representative global analyses. We observed similarities in CCs and capsular genotype distribution and our results are consistent with the global trend of type IV emergence. Additional description of a non-haemolytic cluster of isolates raises questions about screening detection, as some methods lack the capability to detect such isolates. The potential importance of their detection is highlighted as one of these isolates came from a case of neonatal invasive disease. In addition to monitoring prevalent circulating sequence types, our data also provide information on potential therapeutic targets that may be of use in both implementation and assessment of efficacy of future vaccination strategies. Our data indicate that the most appropriate capsular vaccine for this population would be the proposed pentavalent (Cps Ia, Ib, II, III and V) preparation, whilst vaccines targeting surface proteins should ideally utilise the Fbp, Lmb and/or ScpB.

## Supporting information

S1 Table Summary of 151 clinical *Streptococcus agalactiae* isolate characteristics including study and PubMLST identification numbers. (DOCX)

S2 Table Quality statistics for the sequencing and assembly of the 171 *Streptococcus agalactiae* isolates. (DOCX)

S3 Table Isolates included in the global phylogenetic comparison, including characteristics and reference to publication, accession number or PubMLST contributor [25,27–29]. (DOCX)
