# Supplementary material for: Genomic characterisation of perinatal Western Australian Streptococcus agalactiae isolates
Source: PLoS One. 2019 Oct 2;14(10):e0223256. doi: 10.1371/journal.pone.0223256 (PMC6774530; doi:10.1371/journal.pone.0223256)
Supplement: S1 Table — (DOCX) [file pone.0223256.s001.docx]

**Supporting information**

**S1 Table.** Summary of 151 clinical *Streptococcus agalactiae* isolate characteristics including study and PubMLST identification numbers.

|  | | | | |  | Surface protein genes | | | | | | | | |  |  | Haemolysin/cytolysin genes | | | | | | | | | | |  |
| --- | --- | --- | --- | --- | --- | --- | --- | --- | --- | --- | --- | --- | --- | --- | --- | --- | --- | --- | --- | --- | --- | --- | --- | --- | --- | --- | --- | --- |
| PubMLST Isolate ID | **Study ID** | **CC** | **MLST** | **Capsular genotype** | **SAG0433 (*rib)*** | **SAG0832 (Hyp)** | **SAG1052 (*Fbp*)** | **SAG1234 (*lmb*)** | **SAG1236 (*scpB*)** | **SAG2195 (*bac*)** | **SAG2196 (*bca*)** | **AH013348 (*alp1*)** | **SAG2197 (*alp2*)** | **SAG2198 (*alp3*)** | **AJ488912.1 (*alp4I)*** | **Pilus Islands** | **cylD** | **CylG** | **Acyl carrier protein** | **cylZ** | **cylA** | **cylB** | **cylE** | **cylF** | **cylI** | **cylJ** | **cylK** | **SAG1197 (Hyaluronate lyase)** |
| 4532 | II-133_V | 1 | 1 | V | ND | + | ND | + | + | ND | ND | ND | ND | + | ND | 1/2a | + | + | + | + | + | + | + | + | + | + | + | ND |
| 4540 | II-196_V | 1 | 1 | V | ND | + | + | + | + | ND | ND | ND | ND | + | ND | 1/2a | + | + | + | + | + | + | + | + | + | + | + | ND |
| 4574 | II-36_V | 1 | 1 | V | ND | + | ND | + | + | ND | ND | ND | ND | + | ND | 1/2a | + | + | + | + | + | + | + | + | + | + | + | ND |
| 4555 | II-251_V | 1 | 1 | V | ND | ND | + | + | + | ND | ND | ND | ND | + | ND | 1/2a | + | + | + | + | + | + | + | + | + | + | + | + |
| 4493 | 259_V | 1 | 1 | V | ND | + | + | + | + | ND | ND | ND | ND | + | ND | 1/2a | + | + | + | + | + | + | + | + | + | + | + | + |
| 4486 | 218_V | 1 | 1 | V | ND | + | + | + | + | ND | ND | ND | ND | + | ND | 1/2a | + | + | + | + | + | + | + | + | + | + | + | + |
| 4528 | II-108_V | 1 | 1 | V | ND | + | + | + | + | ND | ND | ND | ND | + | ND | 1/2a | + | + | + | + | + | + | + | + | + | + | + | + |
| 4500 | 273_R | 1 | 1 | V | ND | + | + | + | + | ND | ND | ND | ND | + | ND | 1/2a | + | + | + | + | + | + | + | + | + | + | + | + |
| 4550 | II-236_V | 1 | 1 | V | ND | + | + | + | + | ND | ND | ND | ND | + | ND | 1/2a | + | + | + | + | + | + | + | + | + | + | + | + |
| 4635 | Neo_2 | 1 | 1 | V | ND | + | + | + | + | ND | ND | ND | ND | + | ND | 1/2a | + | + | + | + | + | + | ND | + | + | + | + | + |
| 4577 | II-40_V | 1 | 1 | V | ND | + | + | + | + | ND | ND | ND | ND | + | ND | 1/2a | + | + | + | + | + | + | + | + | + | + | + | + |
| 4596 | II-490_V | 1 | 1 | V | ND | + | + | + | + | ND | ND | ND | ND | + | ND | 1/2a | + | + | + | + | + | + | + | + | + | + | + | + |
| 4531 | II-13_V | 1 | 1 | V | ND | + | + | + | + | ND | ND | ND | ND | + | ND | 1/2a | + | + | + | + | + | + | + | + | + | + | + | + |
| 4522 | 54_V | 1 | 1 | V | ND | + | + | + | + | ND | ND | ND | ND | + | ND | 1/2a | + | + | + | + | + | + | + | + | + | + | + | + |
| 4603 | II-54_R | 1 | 1 | V | ND | + | + | + | + | ND | ND | ND | ND | + | ND | 1/2a | + | + | + | + | + | + | + | + | + | + | + | + |
| 4496 | 263_R | 1 | 1 | V | ND | + | + | + | + | ND | ND | ND | ND | + | ND | 1/2a | + | + | + | + | + | + | + | + | + | + | + | + |
| 4497 | 263_V | 1 | 1 | V | ND | + | + | + | + | ND | ND | ND | ND | + | ND | 1/2a | + | + | + | + | + | + | + | + | + | + | + | + |
| 4551 | II-239_V | 1 | 1 | V | ND | ND | + | + | + | ND | ND | ND | ND | + | ND | 1/2a | + | + | + | + | + | + | + | + | + | + | + | + |
| 4557 | II-263_R | 1 | 1 | V | ND | + | + | + | + | ND | ND | ND | ND | + | ND | 1/2a | + | + | + | + | + | + | + | + | + | + | + | + |
| 4561 | II-282_V | 1 | 1 | V | ND | ND | + | + | + | ND | ND | ND | ND | + | ND | 1/2a | + | + | + | + | + | + | + | + | + | + | + | + |
| 4638 | Neo_5 | 1 | 1 | Ib | ND | + | + | + | + | ND | ND | ND | ND | + | ND | 1/2a | + | + | + | + | + | + | + | + | + | + | + | + |
| 4508 | 375_V | 1 | 1 | II | ND | + | + | + | + | ND | ND | ND | ND | + | ND | 1/2a | + | + | + | + | + | + | + | + | + | + | + | + |
| 4579 | II-403_V | 1 | 1 | II | ND | + | + | + | + | ND | ND | ND | ND | + | ND | 1/2a | + | + | + | + | + | + | + | + | + | + | + | + |
| 4525 | II-1_R | 1 | 1 | II | ND | + | + | + | + | ND | ND | ND | ND | + | ND | 1/2a | + | + | + | + | + | + | + | + | + | + | + | + |
| 4526 | II-1_V | 1 | 1 | II | ND | + | + | + | + | ND | ND | ND | ND | + | ND | 1/2a | + | + | + | + | + | + | + | + | + | + | + | + |
| 4499 | 268_V | 1 | 1 | II | ND | + | + | + | + | ND | ND | ND | ND | + | ND | 1/2a | + | + | + | + | + | + | + | + | + | + | + | + |
| 4560 | II-281_V | 1 | 1 | II | ND | + | + | + | + | ND | ND | ND | ND | + | ND | 1/2a | + | + | + | + | + | + | + | + | + | + | + | + |
| 4488 | 23_V | 1 | 196 | IV | ND | + | + | + | + | ND | ND | + | ND | ND | ND | 1/2a | + | + | + | + | + | + | + | + | + | + | + | + |
| 4548 | II-23_V | 1 | 196 | IV | ND | ND | + | + | + | ND | ND | + | ND | ND | ND | 1/2a | + | + | + | + | + | + | + | + | + | + | + | + |
| 4563 | II-284_V | 1 | 196 | IV | ND | + | + | + | + | ND | ND | + | ND | ND | ND | 1/2a | + | + | + | + | + | + | + | + | + | + | + | + |
| 4633 | Neo_1 | 1 | 196 | IV | ND | + | + | + | + | ND | ND | + | ND | ND | ND | 1/2a | + | + | + | + | + | + | + | + | + | + | + | + |
| 4591 | II-454_R | 1 | 2 | VIII | + | + | + | + | + | ND | ND | ND | ND | ND | ND | 1/2b | + | + | + | + | + | + | + | + | + | + | + | + |
| 4510 | 422_V | 1 | 4 | Ia | ND | + | + | + | + | ND | ND | + | ND | ND | ND | 1/2b | + | + | + | + | + | + | + | + | + | + | + | + |
| 4490 | 238_R | 1 | 414 | IV | ND | + | + | + | + | ND | ND | ND | ND | + | ND | 2a | + | + | + | + | + | + | + | + | + | + | + | + |
| 4585 | II-433_V | 1 | 1 | VI | ND | + | + | + | + | ND | + | ND | ND | ND | ND | 1/2a | + | + | + | + | + | + | + | + | + | + | + | + |
| 4608 | II-8_V | 1 | 1 | VI | ND | + | + | + | + | ND | ND | ND | ND | ND | ND | 1/2a | + | + | + | + | + | + | + | + | + | + | + | + |
| 4492 | 254_V | 1 | 1 | VI | ND | + | + | + | + | ND | + | ND | ND | ND | ND | 1/2a | + | + | + | + | + | + | + | + | + | + | + | + |
| 4520 | 496_R | 1 | 1 | VI | ND | + | + | + | + | ND | + | ND | ND | ND | ND | 1/2a | + | + | + | + | + | + | + | + | + | + | + | + |
| 4610 | II-9_R | 1 | 1 | VI | ND | + | + | + | + | ND | + | ND | ND | ND | ND | 1/2a | + | + | + | + | + | + | + | + | + | + | + | + |
| 4549 | II-230_R | 1 | 1 | VI | ND | + | + | + | + | ND | + | ND | ND | ND | ND | 1/2a | + | + | + | + | + | + | + | + | + | + | + | + |
| 4634 | Neo_10 | 1 | 1 | VI | ND | + | + | + | + | ND | + | ND | ND | ND | ND | 1/2a | + | + | + | + | + | + | + | + | + | + | + | + |
| 4539 | II-185_V | 19 | 19 | II | + | + | + | + | + | ND | ND | ND | ND | ND | ND | 1/2a | + | + | + | + | + | + | + | + | + | + | + | + |
| 4513 | 462_V | 19 | 28 | II | + | + | + | + | + | ND | ND | ND | ND | ND | ND | 1/2a | + | + | + | + | + | + | + | + | + | + | + | + |
| 4537 | II-176_V | 19 | 28 | II | + | ND | + | + | + | ND | ND | ND | ND | ND | ND | 1/2a | + | + | + | + | + | + | + | + | + | + | + | + |
| 4601 | II-512_V | 19 | 19 | V | ND | ND | + | + | + | ND | ND | + | ND | ND | ND | 1/2a | + | + | + | + | + | + | + | + | + | + | + | + |
| 4484 | 198_R | 19 | 19 | V | ND | + | + | + | + | ND | ND | + | ND | ND | ND | 1/2a | + | + | + | + | + | + | + | + | + | + | + | + |
| 4502 | 287_V | 19 | 19 | V | ND | + | + | + | + | ND | ND | + | ND | ND | ND | 1/2a | + | + | + | + | + | + | + | + | + | + | + | + |
| 4535 | II-162_V | 19 | 19 | V | ND | + | + | + | + | ND | ND | + | ND | ND | ND | 1/2a | + | + | + | + | + | + | + | + | + | + | + | + |
| 4494 | 26_R | 19 | 19 | V | ND | ND | + | + | + | ND | ND | + | ND | ND | ND | 1/2a | + | + | + | + | + | + | + | + | + | + | + | + |
| 4495 | 26_V | 19 | 19 | V | ND | + | + | + | + | ND | ND | + | ND | ND | ND | 1/2a | + | + | + | + | + | + | + | + | + | + | + | + |
| 4556 | II-26_V | 19 | 19 | V | ND | + | + | + | + | ND | ND | + | ND | ND | ND | 1/2a | + | + | + | + | + | + | + | + | + | + | + | + |
| 4481 | 137_V | 19 | 529 | III | + | + | + | + | + | ND | ND | ND | ND | ND | ND | 1/2a | + | + | + | + | + | + | + | + | + | + | + | ND |
| 4597 | II-493_V | 19 | 27 | III | + | + | + | + | + | ND | ND | ND | ND | ND | ND | 2a | + | + | + | + | ND | + | + | + | + | + | ND | ND |
| 4636 | Neo_3 | 19 | 19 | III | + | + | + | + | + | ND | ND | ND | ND | ND | ND | 1/2a | + | + | + | + | + | + | + | + | + | + | + | ND |
| 4599 | II-507_V | 19 | 19 | III | + | + | + | + | + | ND | ND | ND | ND | ND | ND | 1/2a | + | + | + | + | + | + | + | + | + | + | + | ND |
| 4552 | II-243_R | 19 | 19 | III | + | ND | + | + | + | ND | ND | ND | ND | ND | ND | 1/2a | + | + | + | + | + | + | + | + | + | + | + | ND |
| 4533 | II-155_V | 19 | 335 | III | + | + | + | + | + | ND | ND | ND | ND | ND | ND | 1/2a | + | + | + | + | + | + | + | + | + | + | + | ND |
| 4554 | II-248_V | 19 | 335 | III | + | + | + | + | + | ND | ND | ND | ND | ND | ND | 1/2a | + | + | + | + | + | + | + | + | + | + | + | ND |
| 4475 | 107_V | 19 | 335 | III | + | + | + | + | + | ND | ND | ND | ND | ND | ND | 1/2a | + | + | + | + | + | + | + | + | + | + | + | ND |
| 4504 | 333_V | 19 | 335 | III | + | + | + | + | + | ND | ND | ND | ND | ND | ND | 1/2a | + | + | + | + | + | + | + | + | + | + | + | ND |
| 4512 | 456_V | 19 | 335 | III | + | + | + | + | + | ND | ND | ND | ND | ND | ND | 1/2a | + | + | + | + | + | + | + | + | + | + | + | ND |
| 4501 | 277_V | 19 | 19 | III | + | ND | + | + | + | ND | ND | ND | ND | ND | ND | 1/2a | + | + | + | + | + | + | + | + | + | + | + | ND |
| 4505 | 340_V | 19 | 19 | III | + | + | + | + | + | ND | ND | ND | ND | ND | ND | 1/2a | + | + | + | + | + | + | + | + | + | + | + | ND |
| 4523 | 90_V | 19 | 19 | III | + | + | + | + | + | ND | ND | ND | ND | ND | ND | 1/2a | + | + | + | + | + | + | + | + | + | + | + | ND |
| 4611 | II-90_R | 19 | 19 | III | + | + | + | + | + | ND | ND | ND | ND | ND | ND | 1/2a | + | + | + | + | + | + | + | + | + | + | + | ND |
| 4569 | II-302_V | 19 | 1167 | III | + | + | + | + | + | ND | ND | ND | ND | ND | ND | 1/2a | + | + | + | + | + | + | ND | ND | + | + | + | ND |
| 4514 | 469_V | 19 | 1167 | III | + | + | + | + | + | ND | ND | ND | ND | ND | ND | 1/2a | + | + | + | + | + | + | + | + | + | + | ND | ND |
| 4529 | II-110_V | 19 | 1167 | III | + |  | + | + | + | ND | ND | ND | ND | ND | ND | 1/2a | + | + | + | + | + | + | + | + | + | + | + | ND |
| 4565 | II-292_V | 19 | 861 | III | + | + | + | + | + | ND | ND | ND | ND | ND | ND | 1/2a | + | + | + | + | + | + | + | + | + | + | + | ND |
| 4567 | II-295_V | 19 | 861 | III | + | + | + | + | + | ND | ND | ND | ND | ND | ND | 1/2a | + | + | + | + | + | + | + | + | + | + | + | ND |
| 4576 | II-376_V | 19 | 861 | III | + | + | + | + | + | ND | ND | ND | ND | ND | ND | 1/2a | + | + | + | + | + | + | + | + | + | + | + | ND |
| 4637 | Neo_4 | 12 | 509 | Ia | ND | + | + | + | + | + | + | ND | ND | ND | ND | 1/2a | + | + | + | + | + | + | + | + | + | + | + | + |
| 4491 | 250_V | 12 | 12 | II | ND | + | + | + | + | + | + | ND | ND | ND | ND | 1/2a | + | + | + | + | + | + | + | + | + | + | + | + |
| 4570 | II-315_V | 12 | 12 | II | ND | + | + | + | + | + | + | ND | ND | ND | ND | 1/2a | + | + | + | + | + | + | + | + | + | + | + | + |
| 4503 | 291_V | 12 | 41 | V | + | + | + | + | + | + | ND | ND | ND | ND | ND | 1/2a | ND | ND | ND | ND | ND | ND | ND | ND | ND | ND | ND | ND |
| 4564 | II-291_V | 12 | 41 | V | + | + | + | + | + | + | ND | ND | ND | ND | ND | 1/2a | ND | ND | ND | ND | ND | ND | ND | ND | ND | ND | ND | ND |
| 4642 | Neo_9 | 12 | 585 | V | + | + | + | + | + | + | ND | ND | ND | ND | ND | 1/2a | ND | ND | ND | ND | ND | ND | ND | ND | ND | ND | ND | ND |
| 4485 | 202_V | 12 | 41 | V | + | + | + | + | + | + | ND | ND | ND | ND | ND | 1/2a | ND | ND | ND | ND | ND | ND | ND | ND | ND | ND | ND | ND |
| 4543 | II-202_V | 12 | 41 | V | + | + | + | + | + | + | ND | ND | ND | ND | ND | 1/2a | ND | ND | ND | ND | ND | ND | ND | ND | ND | ND | ND | ND |
| 4587 | II-435_V | 12 | 569 | II | ND | + | + | + | + | + | + | ND | ND | ND | ND | 1/2a | + | + | + | + | + | + | + | + | + | + | + | + |
| 4584 | II-431_V | 12 | 12 | II | ND | + | + | + | + | + | + | ND | ND | ND | ND | 2a | ND | + | + | + | + | + | + | + | + | + | + | + |
| 4598 | II-494_V | 12 | 12 | II | ND | + | + | + | + | + | + | ND | ND | ND | ND | 2a | + | + | + | + | + | + | + | + | + | + | + | + |
| 4483 | 160_V | 12 | 12 | II | ND | + | + | + | + | + | + | ND | ND | ND | ND | 2a | + | + | + | + | + | + | + | + | + | + | + | + |
| 4534 | II-160_V | 12 | 12 | II | ND | ND | + | + | + | + | + | ND | ND | ND | ND | 2a | + | + | + | + | + | + | + | + | + | + | + | + |
| 4530 | II-12_V | 12 | 12 | II | ND | ND | + | + | + | + | + | ND | ND | ND | ND | 1/2a | + | + | + | + | + | + | + | + | + | + | + | + |
| 4538 | II-183_V | 12 | 10 | Ib | ND | + | + | + | + | + | + | ND | ND | ND | ND | 1/2a | + | + | + | + | + | + | + | + | + | + | + | + |
| 4593 | II-458_R | 12 | 12 | Ib | ND | + | + | + | + | + | + | ND | ND | ND | ND | 1/2a | + | + | + | + | + | + | + | + | + | + | + | + |
| 4578 | II-400_V | 12 | 8 | Ib | ND | + | + | + | + | + | + | ND | ND | ND | ND | 1/2a | + | + | + | + | + | + | + | + | + | + | + | + |
| 4639 | Neo_6 | 12 | 8 | Ib | ND | + | + | + | + | + | + | ND | ND | ND | ND | 1/2a | + | ND | + | + | + | + | + | + | + | + | + | + |
| 4595 | II-482_V | 12 | 8 | Ib | ND | + | + | + | + | + | + | ND | ND | ND | ND | 1/2a | + | + | + | + | + | + | + | + | + | + | + | + |
| 4562 | II-283_V | 12 | 8 | Ib | ND | + | + | + | + | + | + | ND | ND | ND | ND | 1/2a | + | + | + | + | + | + | + | + | + | + | + | + |
| 4600 | II-510_V | 12 | 8 | Ib | ND | + | + | + | + | + | + | ND | ND | ND | ND | 1/2a | + | + | + | + | + | + | + | + | + | + | + | + |
| 4473 | 104_V | 12 | 15 | Ib | ND | ND | + | + | + | + | + | ND | ND | ND | ND | 2a | + | + | + | + | + | + | + | + | + | + | + | + |
| 4592 | II-455_V | 12 | 130 | IX | ND | ND | + | + | + | + | + | ND | ND | ND | ND | 2a | + | + | + | + | + | + | + | + | + | + | + | ND |
| 4519 | 495_R | S | 248 | Ia | ND | ND | + | ND | ND | ND | ND | + | ND | ND | ND | 2b | + | + | + | + | + | + | + | + | + | + | + | + |
| 4586 | II-434_V | 17 | 291 | IV | + | ND | + | + | + | ND | ND | ND | ND | ND | ND | 1/2b | + | + | + | + | + | + | + | + | + | + | + | + |
| 4609 | II-84_V | 17 | 17 | III | + | ND | + | + | + | ND | ND | ND | ND | ND | ND | 1/2b | + | + | + | + | + | + | + | + | + | + | + | + |
| 4480 | 132_V | 17 | 17 | III | + | ND | + | + | + | ND | ND | ND | ND | ND | ND | 1/2b | + | + | + | + | + | + | + | + | + | + | + | + |
| 4573 | II-350_V | 17 | 17 | III | + | ND | + | + | + | ND | ND | ND | ND | ND | ND | 1/2b | + | + | + | + | + | + | + | + | + | + | + | + |
| 4640 | Neo_7 | 17 | 17 | III | + | ND | + | + | + | ND | ND | ND | ND | ND | ND | 2b | + | + | + | + | + | + | + | + | + | + | + | + |
| 4524 | 92_V | 17 | 17 | III | + | ND | + | + | + | ND | ND | ND | ND | ND | ND | 1/2b | + | + | + | + | + | + | + | + | + | + | + | + |
| 4498 | 266_V | 17 | 17 | III | + | ND | + | + | + | ND | ND | ND | ND | ND | ND | 1/2b | + | + | + | + | + | + | + | + | + | + | + | + |
| 4541 | II-198_V | 17 | 17 | III | + | ND | + | + | + | ND | ND | ND | ND | ND | ND | 1/2b | + | + | + | + | + | + | + | + | + | + | + | + |
| 4487 | 22_V | 17 | 17 | III | + | ND | + | + | + | ND | ND | ND | ND | ND | ND | 1/2b | + | + | + | + | + | + | + | + | + | + | + | + |
| 4544 | II-22_V | 17 | 17 | III | + | ND | + | + | + | ND | ND | ND | ND | ND | ND | 1/2b | + | + | + | + | + | + | + | + | + | + | + | + |
| 4474 | 105_R | S | 22 | II | ND | ND | + | + | + | ND | + | ND | ND | ND | ND | 2a | ND | ND | ND | ND | ND | ND | ND | ND | ND | ND | ND | + |
| 4518 | 481_V | S | 22 | II | ND | ND | ND | + | + | ND | + | ND | ND | ND | ND | 2a | + | + | + | + | + | + | + | + | + | + | + | + |
| 4521 | 499_V | S | 22 | II | ND | ND | + | + | + | ND | + | ND | ND | ND | ND | 2a | + | + | + | + | + | + | + | + | + | + | + | + |
| 4546 | II-226_V | S | 22 | II | ND | ND | + | + | + | ND | + | ND | ND | ND | ND | 2a | + | + | + | + | + | + | + | + | + | + | + | + |
| 4580 | II-407_V | S | 22 | II | ND | ND | + | + | + | ND | + | ND | ND | ND | ND | 2a | + | + | + | + | + | + | + | + | + | + | + | + |
| 4482 | 152_V | 23 | NEW | Ia | ND | ND | + | ND | ND | ND | ND | ND | + | ND | ND | 1/2a | + | ND | + | + | + | + | + | + | ND | + | + | ND |
| 4547 | II-227_R | 23 | 23 | III | + | ND | + | + | + | ND | ND | ND | ND | ND | ND | 1/2a | + | + | + | + | + | + | + | + | + | + | + | + |
| 4559 | II-279_V | 23 | 88 | Ia | ND | ND | + | + | + | ND | ND | ND | + | ND | ND | 1/2a | + | + | + | + | + | + | + | + | + | + | + | + |
| 4517 | 480_V | 23 | 890 | V | ND | ND | + | + | + | ND | + | ND | ND | ND | ND | 2a | + | + | + | + | + | + | + | + | + | + | + | + |
| 4509 | 408_V | 23 | 24 | V | ND | ND | + | + | + | ND | + | ND | ND | ND | ND | 2a | + | + | + | + | + | + | + | + | + | + | + | + |
| 4590 | II-451_V | 23 | 24 | V | ND | ND | + | + | + | ND | + | ND | ND | ND | ND | 2a | + | + | + | + | + | + | + | + | + | + | + | + |
| 4536 | II-175_V | 23 | 144 | Ia | + | ND | + | + | + | ND | ND | ND | ND | ND | ND | 2a | + | + | + | + | + | + | + | + | + | + | + | + |
| 4582 | II-415_V | 23 | 144 | Ia | + | ND | + | + | + | ND | ND | ND | ND | ND | ND | 2a | + | + | + | + | + | + | + | + | + | + | + | + |
| 4571 | II-320_V | 23 | 23 | Ia | ND | ND | + | + | + | ND | ND | + | ND | ND | ND | 2a | + | + | + | + | + | + | + | + | + | + | + | + |
| 4612 | II-97_V | 23 | 23 | Ia | ND | ND | + | + | + | ND | ND | + | ND | ND | ND | 2a | + | + | + | + | + | + | + | + | + | + | + | + |
| 4575 | II-373_R | 23 | 23 | Ia | ND | ND | + | + | + | ND | ND | + | ND | ND | ND | 2a | + | + | + | + | + | + | + | + | + | + | + | + |
| 4515 | 47_V | 23 | 23 | Ia | ND | ND | + | + | + | ND | ND | + | ND | ND | ND | 2a | + | + | + | + | + | + | + | + | + | + | + | + |
| 4558 | II-263_V | 23 | 23 | Ia | ND | ND | + | + | + | ND | ND | + | ND | ND | ND | 2a | + | + | + | + | + | + | + | + | + | + | + | + |
| 4583 | II-423_V | 23 | 23 | Ia | ND | ND | + | + | + | ND | ND | + | ND | ND | ND | 2a | + | + | + | + | + | + | + | + | + | + | + | + |
| 4568 | II-297_V | 23 | 23 | Ia | ND | ND | + | + | + | ND | ND | + | ND | ND | ND | 2a | + | + | + | + | + | + | + | + | + | + | + | + |
| 4604 | II-60_V | 23 | 23 | Ia | ND | ND | + | + | + | ND | ND | + | ND | ND | ND | 2a | + | + | + | + | + | + | + | + | + | + | + | + |
| 4607 | II-69_V | 23 | 24 | Ia | ND | ND | + | + | + | ND | + | ND | ND | ND | ND | 2a | + | + | + | + | + | + | + | + | + | + | + | + |
| 4594 | II-459_V | 23 | 23 | Ia | ND | ND | + | + | + | ND | ND | + | ND | ND | ND | 2a | + | + | + | + | + | + | + | + | + | + | + | + |
| 4553 | II-245_V | 23 | 23 | Ia | ND | ND | + | + | + | ND | ND | + | ND | ND | ND | 2a | + | + | + | + | + | + | + | + | + | + | + | + |
| 4506 | 344_V | 23 | 23 | Ia | ND | ND | + | + | + | ND | ND | + | ND | ND | ND | 2a | + | + | + | + | + | + | + | + | + | + | + | + |
| 4602 | II-53_V | 23 | 23 | Ia | ND | ND | + | + | + | ND | ND | + | ND | ND | ND | 2a | + | + | + | + | + | + | + | + | + | + | + | + |
| 4545 | II-221_V | 23 | 23 | Ia | ND | ND | + | + | + | ND | ND | + | ND | ND | ND | 2a | + | + | + | + | + | + | + | + | + | + | + | + |
| 4566 | II-294_V | 23 | 23 | Ia | ND | ND | + | + | + | ND | ND | + | ND | ND | ND | 2a | + | + | + | + | + | + | + | + | + | + | + | + |
| 4489 | 231_V | 23 | 23 | Ia | ND | ND | + | + | + | ND | ND | + | ND | ND | ND | 2a | + | + | + | + | + | + | + | + | + | + | + | + |
| 4511 | 437_V | 23 | 23 | Ia | ND | ND | + | + | + | ND | ND | + | ND | ND | ND | 2a | + | + | + | + | + | + | + | + | + | + | + | + |
| 4641 | Neo_8 | 23 | 23 | Ia | ND | ND | + | + | + | ND | ND | + | ND | ND | ND | 2a | + | + | + | + | + | + | + | + | + | + | + | + |
| 4581 | II-409_V | 23 | 23 | Ia | ND | ND | + | + | + | ND | ND | + | ND | ND | ND | 2a | + | + | + | + | + | + | + | + | + | + | + | + |
| 4606 | II-64_V | 23 | 23 | Ia | ND | ND | + | + | + | ND | ND | + | ND | ND | ND | 2a | + | + | + | + | + | + | + | + | + | + | + | + |
| 4507 | 35_V | 23 | 23 | Ia | ND | ND | + | + | + | ND | ND | + | ND | ND | ND | 2a | + | + | + | + | + | + | + | + | + | + | + | + |
| 4476 | 112_R | 23 | 23 | Ia | ND | ND | + | + | + | ND | ND | + | ND | ND | ND | 2a | + | + | + | + | + | + | + | + | + | + | + | + |
| 4477 | 112_V | 23 | 23 | Ia | ND | ND | + | + | + | ND | ND | + | ND | ND | ND | 2a | + | + | + | + | + | + | + | + | + | + | + | + |
| 4572 | II-332_V | 23 | 23 | VI | ND | ND | + | + | + | ND | ND | + | ND | ND | ND | 2a | + | + | + | + | + | + | + | + | + | + | + | + |
| 4542 | II-200_V | 23 | 23 | Ia | ND | ND | + | + | + | ND | ND | + | ND | ND | ND | 2a | + | + | + | + | + | + | + | + | + | + | + | + |
| 4643 | 200_V | 23 | 23 | Ia | ND | ND | + | + | + | ND | ND | + | ND | ND | ND | 2a | + | + | + | + | + | + | + | + | + | + | + | + |
| 4527 | II-10_V | 23 | 23 | Ia | ND | ND | + | + | + | ND | ND | + | ND | ND | ND | 2a | + | + | + | + | + | + | + | + | + | + | + | + |
| 4478 | 121_V | 23 | 23 | Ia | ND | ND | + | + | + | ND | ND | + | ND | ND | ND | 2a | + | + | + | + | + | + | + | + | + | + | + | + |
| 4479 | 123_V | 23 | 23 | Ia | ND | ND | + | + | + | ND | ND | + | ND | ND | ND | 2a | + | + | + | + | + | + | + | + | + | + | + | + |
| 4605 | II-63_V | 23 | 23 | Ia | ND | ND | + | + | + | ND | ND | + | ND | ND | ND | 2a | + | + | + | + | + | + | + | + | + | + | + | + |
| 4589 | II-446_V | 23 | 23 | Ia | ND | ND | + | + | + | ND | ND | + | ND | ND | ND | 2a | + | + | + | + | + | + | + | + | + | + | + | + |
| 4516 | 48_V | 23 | 23 | Ia | ND | ND | + | + | + | ND | ND | ND | ND | ND | ND | 2a | + | + | + | + | + | + | + | + | + | + | + | + |
| 4588 | II-444_V | 23 | 23 | Ia | ND | ND | + | + | + | ND | ND | + | ND | ND | ND | 2a | + | + | + | + | + | + | + | + | + | + | + | + |

ND, Not Detected; CC, Clonal Complex; MLST, Multi=locus sequence type; S, Singleton.
