# Supplementary material for: Genomic characterisation of perinatal Western Australian Streptococcus agalactiae isolates
Source: PLoS One. 2019 Oct 2;14(10):e0223256. doi: 10.1371/journal.pone.0223256 (PMC6774530; doi:10.1371/journal.pone.0223256)
Supplement: S2 Table — (DOCX) [file pone.0223256.s002.docx]

**Supporting information**

**S2 Table.** Quality statistics for the sequencing and assembly of the 171 *Streptococcus agalactiae* isolates.

| **PubMLST ID** | **Study ID** | **Isolate** | **Number of reads** | **Maximum read length** | **Total length (>= 0 bp)** | **Total length (>=1000 bp)** | **# contigs** | **Largest contig** | **Total length** | **GC (%)** |
| --- | --- | --- | --- | --- | --- | --- | --- | --- | --- | --- |
| 4608 | II-8_V | 4608 | 941605 | 301 | 2128043 | 2113024 | 36 | 305017 | 2114806 | 35.34 |
| 4530 | II-12_V | 4530 | 715178 | 301 | 2059442 | 2040622 | 69 | 232006 | 2047279 | 35.21 |
| 4544 | II-22_V | 4544 | 685559 | 301 | 2058130 | 2040513 | 62 | 166043 | 2042018 | 35.28 |
| 4556 | II-26_V | 4556 | 685770 | 301 | 2081427 | 2067955 | 77 | 145973 | 2071020 | 35.46 |
| 4574 | II-36_V | 4574 | 502242 | 301 | 2073833 | 2059540 | 67 | 148983 | 2061459 | 35.34 |
| 4577 | II-40_V | 4577 | 658893 | 301 | 2116293 | 2099579 | 78 | 137564 | 2106342 | 35.2 |
| 4602 | II-53_V | 4602 | 618580 | 301 | 1972756 | 1957312 | 58 | 137911 | 1962262 | 35.19 |
| 4604 | II-60_V | 4604 | 627720 | 301 | 2023096 | 2010569 | 51 | 219682 | 2012078 | 35.35 |
| 4605 | II-63_V | 4605 | 669825 | 301 | 2046390 | 2034457 | 45 | 263381 | 2036548 | 35.15 |
| 4606 | II-64_V | 4606 | 787874 | 301 | 1961963 | 1950105 | 32 | 263573 | 1950939 | 35.18 |
| 4607 | II-69_V | 4607 | 1146300 | 301 | 1997544 | 1978108 | 63 | 131967 | 1980071 | 35.22 |
| 4609 | II-84_V | 4609 | 736335 | 301 | 2191047 | 1932350 | 87 | 134277 | 1942839 | 35.26 |
| 4612 | II-97_V | 4612 | 488892 | 301 | 2127488 | 2067627 | 75 | 125067 | 2074325 | 35.34 |
| 4528 | II-108_V | 4528 | 792244 | 301 | 2061519 | 2046736 | 53 | 261957 | 2049696 | 35.3 |
| 4529 | II-110_V | 4529 | 537462 | 301 | 2055533 | 2035777 | 115 | 111076 | 2042596 | 35.46 |
| 4533 | II-155_V | 4533 | 759434 | 301 | 2182590 | 2158471 | 86 | 120796 | 2164113 | 35.41 |
| 4534 | II-160_V | 4534 | 484076 | 301 | 2134179 | 2111859 | 144 | 136765 | 2122945 | 35.37 |
| 4535 | II-162_V | 4535 | 523538 | 301 | 2100516 | 2087084 | 93 | 115919 | 2090975 | 35.49 |
| 4536 | II-175_V | 4536 | 837014 | 301 | 2077857 | 2061563 | 74 | 172474 | 2064376 | 35.39 |
| 4537 | II-176_V | 4537 | 562991 | 301 | 2074296 | 2060239 | 74 | 120228 | 2067622 | 35.26 |
| 4539 | II-185_V | 4539 | 593377 | 301 | 2043465 | 2034109 | 46 | 270460 | 2035499 | 35.25 |
| 4540 | II-196_V | 4540 | 681477 | 301 | 2089997 | 2072502 | 76 | 189526 | 2075454 | 35.39 |
| 4541 | II-198_V | 4541 | 793104 | 301 | 2130589 | 2115765 | 75 | 165608 | 2117572 | 35.46 |
| 4542 | II-200_V | 4542 | 1637820 | 301 | 2036858 | 2009610 | 31 | 394840 | 2013084 | 35.18 |
| 4545 | II-221_V | 4545 | 604199 | 301 | 2143457 | 2014255 | 70 | 164193 | 2025721 | 35.24 |
| 4546 | II-226_V | 4546 | 652806 | 301 | 2052302 | 2041794 | 104 | 122104 | 2042712 | 35.28 |
| 4550 | II-236_V | 4550 | 476082 | 301 | 2066562 | 2052807 | 52 | 137569 | 2055706 | 35.29 |
| 4551 | II-239_V | 4551 | 572175 | 301 | 2115180 | 2087825 | 89 | 150352 | 2095500 | 35.39 |
| 4554 | II-248_V | 4554 | 455852 | 301 | 2140623 | 2131076 | 64 | 196277 | 2133126 | 35.35 |
| 4555 | II-251_V | 4555 | 560972 | 301 | 2118905 | 2101431 | 61 | 193610 | 2108080 | 35.26 |
| 4559 | II-279_V | 4559 | 456677 | 301 | 2078246 | 2060904 | 63 | 171635 | 2065767 | 35.33 |
| 4560 | II-281_V | 4560 | 441428 | 301 | 2053098 | 2039866 | 73 | 129064 | 2043282 | 35.3 |
| 4561 | II-282_V | 4561 | 500435 | 301 | 2155142 | 2081016 | 68 | 140635 | 2084605 | 35.36 |
| 4562 | II-283_V | 4562 | 528937 | 301 | 2054450 | 2041450 | 61 | 183868 | 2042914 | 35.26 |
| 4563 | II-284_V | 4563 | 667781 | 301 | 2206217 | 2185524 | 86 | 159656 | 2191785 | 35.42 |
| 4566 | II-294_V | 4566 | 725938 | 301 | 2087411 | 2008715 | 70 | 172774 | 2015348 | 35.35 |
| 4567 | II-295_V | 4567 | 523827 | 301 | 2419247 | 2239959 | 109 | 181574 | 2253118 | 35.53 |
| 4568 | II-297_V | 4568 | 711335 | 301 | 2140453 | 2104061 | 66 | 126112 | 2109029 | 35.45 |
| 4569 | II-302_V | 4569 | 472052 | 301 | 2152374 | 2060736 | 179 | 73574 | 2075141 | 35.5 |
| 4570 | II-315_V | 4570 | 557635 | 301 | 2146533 | 2135106 | 76 | 139417 | 2138896 | 35.35 |
| 4571 | II-320_V | 4571 | 638233 | 301 | 2046855 | 2020720 | 71 | 137776 | 2028096 | 35.2 |
| 4572 | II-332_V | 4572 | 659133 | 301 | 2157633 | 2000685 | 118 | 151701 | 2045352 | 35.43 |
| 4573 | II-350_V | 4573 | 692733 | 301 | 2000211 | 1983458 | 91 | 109523 | 1985947 | 35.28 |
| 4576 | II-376_V | 4576 | 548863 | 301 | 2103555 | 2092453 | 87 | 111206 | 2094122 | 35.5 |
| 4578 | II-400_V | 4578 | 646575 | 301 | 2125799 | 2110385 | 103 | 110922 | 2117993 | 35.16 |
| 4579 | II-403_V | 4579 | 477626 | 301 | 2121881 | 2105675 | 58 | 321808 | 2108530 | 35.42 |
| 4580 | II-407_V | 4580 | 552713 | 301 | 2100685 | 2084814 | 145 | 101620 | 2089468 | 35.34 |
| 4581 | II-409_V | 4581 | 529925 | 301 | 2048432 | 2030536 | 78 | 131827 | 2034490 | 35.36 |
| 4582 | II-415_V | 4582 | 461430 | 301 | 2147871 | 2035167 | 50 | 290123 | 2038290 | 35.27 |
| 4583 | II-423_V | 4583 | 514714 | 301 | 2053617 | 2041314 | 61 | 155736 | 2044489 | 35.36 |
| 4585 | II-433_V | 4585 | 534076 | 301 | 2048285 | 2033352 | 37 | 368605 | 2033937 | 35.31 |
| 4586 | II-434_V | 4586 | 660126 | 301 | 2024549 | 1985894 | 53 | 166056 | 1987020 | 35.22 |
| 4587 | II-435_V | 4587 | 552718 | 301 | 2013399 | 1998818 | 41 | 199330 | 1999678 | 35.11 |
| 4588 | II-444_V | 4588 | 552169 | 301 | 2086950 | 2075868 | 40 | 267206 | 2077438 | 35.38 |
| 4589 | II-446_V | 4589 | 472067 | 301 | 2761856 | 2065631 | 160 | 267449 | 2133639 | 35.38 |
| 4590 | II-451_V | 4590 | 282328 | 301 | 2044012 | 2019812 | 160 | 77988 | 2032862 | 35.26 |
| 4592 | II-455_V | 4592 | 472502 | 301 | 2150719 | 2135132 | 80 | 202633 | 2141655 | 35.57 |
| 4594 | II-459_V | 4594 | 543218 | 301 | 2589057 | 2053967 | 172 | 172175 | 2127167 | 35.42 |
| 4595 | II-482_V | 4595 | 552617 | 301 | 2104818 | 2088655 | 83 | 176053 | 2095680 | 35.33 |
| 4596 | II-490_V | 4596 | 596780 | 301 | 2060782 | 2044793 | 67 | 166904 | 2048919 | 35.31 |
| 4598 | II-494_V | 4598 | 522388 | 301 | 2183364 | 2172738 | 84 | 137103 | 2174392 | 35.34 |
| 4599 | II-507_V | 4599 | 673839 | 301 | 2200482 | 2176326 | 95 | 182626 | 2184159 | 35.55 |
| 4600 | II-510_V | 4600 | 823680 | 301 | 2069277 | 2051143 | 36 | 283315 | 2053968 | 35.27 |
| 4601 | II-512_V | 4601 | 713343 | 301 | 2157988 | 2142892 | 86 | 168285 | 2145133 | 35.6 |
| 4548 | II-23_V | 4548 | 614827 | 301 | 2132660 | 2107980 | 102 | 118194 | 2119771 | 35.6 |
| 4538 | II-183_V | 4538 | 657416 | 301 | 2035922 | 2016860 | 93 | 111080 | 2023726 | 35.29 |
| 4543 | II-202_V | 4543 | 597282 | 301 | 2161639 | 2132876 | 117 | 105247 | 2146003 | 35.38 |
| 4564 | II-291_V | 4564 | 597561 | 301 | 2078337 | 2065363 | 57 | 206382 | 2067025 | 35.37 |
| 4584 | II-431_V | 4584 | 605662 | 301 | 2137685 | 2129651 | 52 | 289685 | 2130511 | 35.27 |
| 4597 | II-493_V | 4597 | 1000150 | 301 | 2246283 | 2223935 | 86 | 159766 | 2228822 | 35.47 |
| 4526 | II-1_V | 4526 | 610446 | 301 | 2078822 | 2066445 | 67 | 189929 | 2068971 | 35.33 |
| 4525 | II-1_R | 4525 | 460120 | 301 | 2080243 | 2065765 | 57 | 124697 | 2068932 | 35.33 |
| 4527 | II-10_V | 4527 | 701592 | 301 | 2025798 | 2009548 | 47 | 224932 | 2013534 | 35.19 |
| 4531 | II-13_V | 4531 | 670840 | 301 | 2125865 | 2100195 | 69 | 283609 | 2107079 | 35.32 |
| 4532 | II-133_V | 4532 | 493393 | 301 | 2067933 | 2040982 | 105 | 100204 | 2048548 | 35.32 |
| 4565 | II-292_V | 4565 | 772801 | 301 | 2124011 | 2108173 | 70 | 172297 | 2112270 | 35.53 |
| 4553 | II-245_V | 4553 | 951258 | 301 | 2038700 | 2014926 | 31 | 268768 | 2015760 | 35.44 |
| 4610 | II-9_R | 4610 | 470165 | 301 | 2083872 | 2065792 | 63 | 251466 | 2071107 | 35.32 |
| 4603 | II-54_R | 4603 | 612882 | 301 | 2170510 | 2151412 | 78 | 162198 | 2158383 | 35.52 |
| 4611 | II-90_R | 4611 | 587603 | 301 | 2088115 | 2075365 | 98 | 165329 | 2080163 | 35.52 |
| 4547 | II-227_R | 4547 | 680632 | 301 | 2096474 | 2077875 | 82 | 125757 | 2083413 | 35.4 |
| 4549 | II-230_R | 4549 | 538294 | 301 | 2079447 | 2063269 | 91 | 102426 | 2070839 | 35.43 |
| 4552 | II-243_R | 4552 | 523576 | 301 | 2080198 | 2056645 | 105 | 101589 | 2062960 | 35.25 |
| 4575 | II-373_R | 4575 | 542772 | 301 | 2399127 | 1965971 | 148 | 132169 | 2017060 | 35.21 |
| 4591 | II-454_R | 4591 | 531116 | 301 | 2022230 | 2011199 | 68 | 207476 | 2014538 | 35.37 |
| 4593 | II-458_R | 4593 | 555583 | 301 | 2186327 | 2176598 | 42 | 248818 | 2177458 | 35.11 |
| 4487 | 22_V | 4487 | 592257 | 301 | 2164662 | 2037825 | 94 | 192764 | 2061904 | 35.24 |
| 4488 | 23_V | 4488 | 549279 | 301 | 2145619 | 2115537 | 84 | 273275 | 2122551 | 35.59 |
| 4507 | 35_V | 4507 | 721220 | 301 | 2030025 | 2011661 | 26 | 488450 | 2013217 | 35.18 |
| 4515 | 47_V | 4515 | 403968 | 301 | 1974287 | 1958934 | 31 | 297107 | 1960987 | 35.17 |
| 4516 | 48_V | 4516 | 892153 | 301 | 2156841 | 2006896 | 83 | 428958 | 2045065 | 35.13 |
| 4522 | 54_V | 4522 | 623199 | 301 | 2131631 | 2114951 | 49 | 151821 | 2118439 | 35.42 |
| 4523 | 90_V | 4523 | 505557 | 301 | 2089794 | 2081482 | 65 | 247532 | 2082342 | 35.49 |
| 4524 | 92_V | 4524 | 508326 | 301 | 2037865 | 2017348 | 43 | 280210 | 2017348 | 35.3 |
| 4473 | 104_V | 4473 | 1081744 | 301 | 2056131 | 2035547 | 34 | 280984 | 2036407 | 35.2 |
| 4474 | 105_R | 4474 | 615542 | 301 | 2054877 | 2015045 | 89 | 140379 | 2016361 | 35.3 |
| 4475 | 107_V | 4475 | 803848 | 301 | 2147197 | 2119138 | 51 | 196477 | 2120604 | 35.37 |
| 4477 | 112_V | 4477 | 727178 | 301 | 2074973 | 2060453 | 30 | 263864 | 2062009 | 35.29 |
| 4476 | 112_R | 4476 | 1086885 | 301 | 2077240 | 2060325 | 25 | 488409 | 2061881 | 35.29 |
| 4478 | 121_V | 4478 | 581892 | 301 | 2023661 | 2010691 | 32 | 267486 | 2012789 | 35.18 |
| 4479 | 123_V | 4479 | 422803 | 301 | 2057099 | 2037178 | 29 | 446358 | 2038529 | 35.15 |
| 4480 | 132_V | 4480 | 1114522 | 301 | 2055202 | 2036094 | 43 | 244042 | 2036094 | 35.12 |
| 4481 | 137_V | 4481 | 555523 | 301 | 2250466 | 2227780 | 95 | 120970 | 2234208 | 35.52 |
| 4482 | 152_V | 4482 | 1383167 | 301 | 2049141 | 1962368 | 306 | 40298 | 1987475 | 34.96 |
| 4483 | 160_V | 4483 | 852998 | 301 | 2143860 | 2130469 | 50 | 215328 | 2131329 | 35.31 |
| 4643 | 200_V | 4643 | 649588 | 301 | 2025505 | 2012209 | 29 | 488205 | 2013765 | 35.18 |
| 4485 | 202_V | 4485 | 520759 | 301 | 2167683 | 2147885 | 62 | 286319 | 2150621 | 35.36 |
| 4486 | 218_V | 4486 | 797837 | 301 | 2070827 | 2057425 | 29 | 242034 | 2057425 | 35.2 |
| 4489 | 231_V | 4489 | 654259 | 301 | 2060128 | 2048307 | 27 | 361056 | 2049646 | 35.34 |
| 4490 | 238_R | 4490 | 762433 | 301 | 2028082 | 2014192 | 50 | 173410 | 2015793 | 35.16 |
| 4491 | 250_V | 4491 | 193605 | 301 | 2181250 | 2164775 | 66 | 137103 | 2169283 | 35.41 |
| 4492 | 254_V | 4492 | 618544 | 301 | 2096044 | 2084289 | 42 | 190009 | 2085768 | 35.38 |
| 4493 | 259_V | 4493 | 373667 | 301 | 2067332 | 2052271 | 32 | 318215 | 2052271 | 35.28 |
| 4498 | 266_V | 4498 | 572298 | 301 | 2031366 | 2000100 | 40 | 334213 | 2000100 | 35.35 |
| 4499 | 268_V | 4499 | 554901 | 301 | 2051581 | 2036829 | 48 | 121735 | 2038322 | 35.29 |
| 4500 | 273_R | 4500 | 560016 | 301 | 2077847 | 2055369 | 31 | 320987 | 2056233 | 35.27 |
| 4501 | 277_V | 4501 | 142969 | 301 | 2112170 | 2095805 | 195 | 57405 | 2102897 | 35.51 |
| 4502 | 287_V | 4502 | 427832 | 301 | 2165659 | 2132987 | 65 | 265783 | 2139958 | 35.59 |
| 4504 | 333_V | 4504 | 658481 | 301 | 2076039 | 2066660 | 72 | 194567 | 2069716 | 35.23 |
| 4505 | 340_V | 4505 | 611157 | 301 | 2144440 | 2125542 | 90 | 149306 | 2133134 | 35.46 |
| 4506 | 344_V | 4506 | 1024638 | 301 | 1999864 | 1979885 | 36 | 310691 | 1980719 | 35.18 |
| 4508 | 375_V | 4508 | 966115 | 301 | 2086456 | 2071745 | 38 | 321518 | 2074537 | 35.36 |
| 4509 | 408_V | 4509 | 655059 | 301 | 2048438 | 2036961 | 32 | 234574 | 2037795 | 35.22 |
| 4510 | 422_V | 4510 | 625354 | 301 | 2101539 | 2090395 | 59 | 227304 | 2091568 | 35.37 |
| 4511 | 437_V | 4511 | 555844 | 301 | 2059189 | 2040788 | 63 | 157415 | 2045253 | 35.35 |
| 4512 | 456_V | 4512 | 712959 | 301 | 2177377 | 2160952 | 66 | 196278 | 2164119 | 35.39 |
| 4513 | 462_V | 4513 | 1371637 | 301 | 2077867 | 2062509 | 31 | 204375 | 2064004 | 35.34 |
| 4514 | 469_V | 4514 | 517326 | 301 | 2089907 | 2077434 | 88 | 127852 | 2080488 | 35.5 |
| 4517 | 480_V | 4517 | 564526 | 301 | 2145427 | 2107006 | 67 | 140809 | 2112448 | 35.43 |
| 4518 | 481_V | 4518 | 506076 | 301 | 2087969 | 2078935 | 118 | 129033 | 2080205 | 35.36 |
| 4519 | 495_R | 4519 | 598802 | 301 | 2142854 | 2119883 | 48 | 234651 | 2123872 | 35.39 |
| 4520 | 496_R | 4520 | 744181 | 301 | 2131749 | 2115467 | 53 | 327193 | 2116857 | 35.32 |
| 4521 | 499_V | 4521 | 938395 | 301 | 2008436 | 1994290 | 109 | 90164 | 1996285 | 35.28 |
| 4497 | 263_V | 4497 | 806801 | 301 | 2113988 | 2094901 | 53 | 186072 | 2096354 | 35.37 |
| 4496 | 263_R | 4496 | 640933 | 301 | 2110899 | 2093249 | 63 | 176915 | 2096377 | 35.37 |
| 4558 | II-263_V | 4558 | 683513 | 301 | 2058443 | 2049052 | 36 | 264469 | 2049886 | 35.41 |
| 4557 | II-263_R | 4557 | 712878 | 301 | 2245765 | 2096531 | 65 | 152485 | 2102105 | 35.39 |
| 4495 | 26_V | 4495 | 901764 | 301 | 2081862 | 2071308 | 54 | 247521 | 2072168 | 35.44 |
| 4494 | 26_R | 4494 | 474811 | 301 | 2081283 | 2069056 | 87 | 115961 | 2072801 | 35.45 |
| 4503 | 291_V | 4503 | 496613 | 301 | 2080707 | 2062767 | 73 | 159964 | 2068698 | 35.39 |
| 4484 | 198_R | 4484 | 709269 | 301 | 2135964 | 2118570 | 70 | 159005 | 2120999 | 35.54 |
| 4633 | Neo_1 | 4633 | 586117 | 301 | 2122925 | 2106271 | 65 | 197883 | 2111654 | 35.46 |
| 4635 | Neo_2 | 4635 | 620745 | 301 | 2116624 | 2052913 | 72 | 134697 | 2054944 | 35.3 |
| 4636 | Neo_3 | 4636 | 536494 | 301 | 2124759 | 2115654 | 74 | 201606 | 2117657 | 35.3 |
| 4637 | Neo_4 | 4637 | 444959 | 301 | 2175495 | 2160331 | 104 | 128785 | 2167663 | 35.43 |
| 4638 | Neo_5 | 4638 | 454580 | 301 | 2054115 | 2043370 | 62 | 153470 | 2046420 | 35.3 |
| 4639 | Neo_6 | 4639 | 588567 | 301 | 2068801 | 2058155 | 66 | 201666 | 2061733 | 35.14 |
| 4640 | Neo_7 | 4640 | 529453 | 301 | 2182794 | 2168740 | 95 | 95537 | 2171687 | 35.45 |
| 4641 | Neo_8 | 4641 | 454094 | 301 | 2043845 | 2028373 | 42 | 187122 | 2029207 | 35.26 |
| 4642 | Neo_9 | 4642 | 572438 | 301 | 2123047 | 2055315 | 110 | 163543 | 2063871 | 35.43 |
| 4634 | Neo_10 | 4634 | 593101 | 301 | 2069590 | 2058382 | 54 | 254574 | 2061416 | 35.34 |
