# Supplementary material for: Genomic characterisation of perinatal Western Australian Streptococcus agalactiae isolates
Source: PLoS One. 2019 Oct 2;14(10):e0223256. doi: 10.1371/journal.pone.0223256 (PMC6774530; doi:10.1371/journal.pone.0223256)
Supplement: S3 Table — (DOCX) [file pone.0223256.s003.docx]

**Supporting information**

**S3 Table.** Isolates included in the global phylogenetic comparison, including characteristics and reference to publication, accession number or PubMLST contributor [60, 62-64].

| **PubMLST ID** | **Reference/Accession number** | **Isolate ID** | **Continent** | **Year** | **ST** |
| --- | --- | --- | --- | --- | --- |
| 709 | [GCA_000289375.1](https://www.ebi.ac.uk/ena/data/view/GCA_000289375.1) | MRI Z1-213 | Europe | 2004 | 591 |
| 710 | [GCA_000311305.1](https://www.ebi.ac.uk/ena/data/view/GCA_000311305.1) | MRI Z1-215 | Europe | 2004 | 589 |
| 711 | [GCA_000322845.1](https://www.ebi.ac.uk/ena/data/view/GCA_000322845.1) | MRI Z1-218 | Europe | 2005 | 590 |
| 745 | [GCA_000427055.1](https://www.ebi.ac.uk/ena/data/view/GCA_000427055.1) | ILRI112 | Africa | 2002 | 617 |
| 763 | [GCA_000427075.1](https://www.ebi.ac.uk/ena/data/view/GCA_000427075.1) | ILRI005 | Africa | 2004 | 609 |
| 1062 | SAMEA4027055 | ERR1624735 | Europe |  | 6 |
| 1130 | SAMEA4027062 | ERR1624742 | Europe |  | 10 |
| 1135 | SAMEA4027063 | ERR1624743 | Europe |  | 6 |
| 1164 | AE009948.1 | 2603V/R |  |  | 110 |
| 1174 | [GCA_000221325.2](https://www.ebi.ac.uk/ena/data/view/GCA_000221325.2) | FSL S3-026 |  |  | 67 |
| 1178 | [GCA_000311405.1](https://www.ebi.ac.uk/ena/data/view/GCA_000311405.1) | BSU133 |  |  | 6 |
| 1181 | [GCA_000310625.1](https://www.ebi.ac.uk/ena/data/view/GCA_000310625.1) | BSU174 |  |  | 41 |
| 1184 | [GCA_000289895.1](https://www.ebi.ac.uk/ena/data/view/GCA_000289895.1) | BSU247 |  |  | 26 |
| 1188 | [GCA_000311365.1](https://www.ebi.ac.uk/ena/data/view/GCA_000311365.1) | BSU260 |  |  | 88 |
| 1189 | [GCA_000289055.1](https://www.ebi.ac.uk/ena/data/view/GCA_000289055.1) | BSU442 |  |  | 22 |
| 1192 | [GCA_000311385.1](https://www.ebi.ac.uk/ena/data/view/GCA_000311385.1) | BSU451 |  |  | 103 |
| 1196 | [GCA_000510405.1](https://www.ebi.ac.uk/ena/data/view/GCA_000510405.1) | BV3L5 |  |  | 110 |
| 1197 | [GCA_000311185.1](https://www.ebi.ac.uk/ena/data/view/GCA_000311185.1) | CCUG 17336 | Europe | 1985 | 17 |
| 1200 | [GCA_000288715.1](https://www.ebi.ac.uk/ena/data/view/GCA_000288715.1) | CCUG 25532 |  |  | 26 |
| 1203 | [GCA_000310485.1](https://www.ebi.ac.uk/ena/data/view/GCA_000310485.1) | CCUG 29782 | Europe | 1992 | 23 |
| 1205 | [GCA_000323085.1](https://www.ebi.ac.uk/ena/data/view/GCA_000323085.1) | CCUG 34230 | Europe | 1995 | 23 |
| 1215 | [GCA_000288855.1](https://www.ebi.ac.uk/ena/data/view/GCA_000288855.1) | CCUG 39096 A |  |  | 9 |
| 1220 | [GCA_000310525.1](https://www.ebi.ac.uk/ena/data/view/GCA_000310525.1) | CCUG 44110 |  |  | 88 |
| 1222 | [GCA_000311245.1](https://www.ebi.ac.uk/ena/data/view/GCA_000311245.1) | CCUG 44186 | Europe | 2000 | 17 |
| 1225 | [GCA_000311625.1](https://www.ebi.ac.uk/ena/data/view/GCA_000311625.1) | CCUG 49072 |  |  | 524 |
| 1231 | [GCA_000310285.1](https://www.ebi.ac.uk/ena/data/view/GCA_000310285.1) | FSL C1-487 |  |  | 415 |
| 1234 | [GCA_000289875.1](https://www.ebi.ac.uk/ena/data/view/GCA_000289875.1) | FSL F2-343 |  |  | 88 |
| 1237 | [GCA_000310465.1](https://www.ebi.ac.uk/ena/data/view/GCA_000310465.1) | FSL S3-005 |  |  | 22 |
| 1241 | [GCA_000322565.1](https://www.ebi.ac.uk/ena/data/view/GCA_000322565.1) | FSL S3-034 |  |  | 61 |
| 1242 | [GCA_000323145.1](https://www.ebi.ac.uk/ena/data/view/GCA_000323145.1) | FSL S3-043 |  |  | 61 |
| 1246 | [GCA_000310445.1](https://www.ebi.ac.uk/ena/data/view/GCA_000310445.1) | FSL S3-102 |  |  | 31 |
| 1251 | [GCA_000323125.1](https://www.ebi.ac.uk/ena/data/view/GCA_000323125.1) | FSL S3-229 |  |  | 415 |
| 1253 | [GCA_000311145.1](https://www.ebi.ac.uk/ena/data/view/GCA_000311145.1) | FSL S3-268 |  |  | 22 |
| 1257 | [GCA_000322605.1](https://www.ebi.ac.uk/ena/data/view/GCA_000322605.1) | FSL S3-568 |  |  | 415 |
| 1258 | [GCA_000310365.1](https://www.ebi.ac.uk/ena/data/view/GCA_000310365.1) | FSL S3-586 |  |  | 67 |
| 1259 | [GCA_000289815.1](https://www.ebi.ac.uk/ena/data/view/GCA_000289815.1) | FSL S3-603 |  |  | 61 |
| 1260 | [GCA_000322785.1](https://www.ebi.ac.uk/ena/data/view/GCA_000322785.1) | FSL S3-608 |  |  | 490 |
| 1261 | [GCA_000322825.1](https://www.ebi.ac.uk/ena/data/view/GCA_000322825.1) | FSL S3-654 |  |  | 61 |
| 1272 | Singh et al. (2012) | GB00112 | North America | 1999 | 17 |
| 1274 | [GCA_000290115.1](https://www.ebi.ac.uk/ena/data/view/GCA_000290115.1) | GB00174 |  |  | 22 |
| 1283 | [GCA_000289575.1](https://www.ebi.ac.uk/ena/data/view/GCA_000289575.1) | GB00300 |  |  | 130 |
| 1285 | [GCA_000290175.1](https://www.ebi.ac.uk/ena/data/view/GCA_000290175.1) | GB00543 |  |  | 36 |
| 1286 | [GCA_000290195.1](https://www.ebi.ac.uk/ena/data/view/GCA_000290195.1) | GB00548 |  |  | 88 |
| 1290 | [GCA_000290295.1](https://www.ebi.ac.uk/ena/data/view/GCA_000290295.1) | GB00588 |  |  | 447 |
| 1291 | [GCA_000290315.1](https://www.ebi.ac.uk/ena/data/view/GCA_000290315.1) | GB00601 |  |  | 24 |
| 1292 | [GCA_000290335.1](https://www.ebi.ac.uk/ena/data/view/GCA_000290335.1) | GB00614 |  |  | 448 |
| 1293 | [GCA_000290355.1](https://www.ebi.ac.uk/ena/data/view/GCA_000290355.1) | GB00640 |  |  | 26 |
| 1305 | [GCA_000289675.1](https://www.ebi.ac.uk/ena/data/view/GCA_000289675.1) | GB00888 |  |  | 41 |
| 1311 | [GCA_000288295.1](https://www.ebi.ac.uk/ena/data/view/GCA_000288295.1) | GB00901 |  |  | 459 |
| 1316 | [GCA_000288435.1](https://www.ebi.ac.uk/ena/data/view/GCA_000288435.1) | GB00922 |  |  | 88 |
| 1320 | [GCA_000288555.1](https://www.ebi.ac.uk/ena/data/view/GCA_000288555.1) | GB00933 |  |  | 452 |
| 1323 | [GCA_000322905.1](https://www.ebi.ac.uk/ena/data/view/GCA_000322905.1) | GB00951 |  |  | 28 |
| 1324 | [GCA_000322645.1](https://www.ebi.ac.uk/ena/data/view/GCA_000322645.1) | GB00954 |  |  | 22 |
| 1329 | [GCA_000322665.1](https://www.ebi.ac.uk/ena/data/view/GCA_000322665.1) | GB00965 |  |  | 88 |
| 1330 | [GCA_000288635.1](https://www.ebi.ac.uk/ena/data/view/GCA_000288635.1) | GB00975 |  |  | 22 |
| 1336 | [GCA_000322685.1](https://www.ebi.ac.uk/ena/data/view/GCA_000322685.1) | GB01004 |  |  | 22 |
| 1339 | [GCA_000419215.1](https://www.ebi.ac.uk/ena/data/view/GCA_000419215.1) | GBS12 |  |  | 88 |
| 1344 | [GCA_000310265.1](https://www.ebi.ac.uk/ena/data/view/GCA_000310265.1) | LDS 623 |  |  | 61 |
| 1345 | [GCA_000288055.1](https://www.ebi.ac.uk/ena/data/view/GCA_000288055.1) | LDS 628 |  |  | 61 |
| 1350 | [GCA_000310805.1](https://www.ebi.ac.uk/ena/data/view/GCA_000310805.1) | LMG 15081 |  |  | 25 |
| 1356 | [GCA_000288935.1](https://www.ebi.ac.uk/ena/data/view/GCA_000288935.1) | LMG 15091 |  |  | 1166 |
| 1358 | [GCA_000288975.1](https://www.ebi.ac.uk/ena/data/view/GCA_000288975.1) | LMG 15093 |  |  | 110 |
| 1366 | [GCA_000419095.1](https://www.ebi.ac.uk/ena/data/view/GCA_000419095.1) | MC627 |  |  | 22 |
| 1368 | [GCA_000419055.1](https://www.ebi.ac.uk/ena/data/view/GCA_000419055.1) | MC629 |  |  | 88 |
| 1374 | [GCA_000310685.1](https://www.ebi.ac.uk/ena/data/view/GCA_000310685.1) | MRI Z1-022 |  |  | 121 |
| 1375 | [GCA_000311425.1](https://www.ebi.ac.uk/ena/data/view/GCA_000311425.1) | MRI Z1-023 |  |  | 103 |
| 1378 | [GCA_000310705.1](https://www.ebi.ac.uk/ena/data/view/GCA_000310705.1) | MRI Z1-035 |  |  | 88 |
| 1379 | [GCA_000289355.2](https://www.ebi.ac.uk/ena/data/view/GCA_000289355.2) | MRI Z1-038 |  |  | 296 |
| 1395 | [GCA_000289395.1](https://www.ebi.ac.uk/ena/data/view/GCA_000289395.1) | MRI Z1-214 |  |  | 591 |
| 1396 | [GCA_000289415.1](https://www.ebi.ac.uk/ena/data/view/GCA_000289415.1) | MRI Z1-216 |  |  | 591 |
| 1418 | [GCA_000347415.1](https://www.ebi.ac.uk/ena/data/view/GCA_000347415.1) | SS1014 |  |  | 6 |
| 1425 | [GCA_000310305.1](https://www.ebi.ac.uk/ena/data/view/GCA_000310305.1) | STIR-CD-14 |  |  | 491 |
| 1432 | [GCA_000310325.1](https://www.ebi.ac.uk/ena/data/view/GCA_000310325.1) | STIR-CD-26 |  |  | 500 |
| 1434 | [GCA_000289255.1](https://www.ebi.ac.uk/ena/data/view/GCA_000289255.1) | STIR-CD-28 |  |  | 500 |
| 1437 | [GCA_000288075.1](https://www.ebi.ac.uk/ena/data/view/GCA_000288075.1) | Gottschalk 1003A |  |  | 19 |
| 1438 | [GCA_000289435.1](https://www.ebi.ac.uk/ena/data/view/GCA_000289435.1) | Gottschalk 1005B |  |  | 288 |
| 1448 | [GCA_000288115.1](https://www.ebi.ac.uk/ena/data/view/GCA_000288115.1) | GB00018 |  |  | 444 |
| 1449 | [GCA_000290075.1](https://www.ebi.ac.uk/ena/data/view/GCA_000290075.1) | GB00111 |  |  | 32 |
| 1451 | [GCA_000288275.1](https://www.ebi.ac.uk/ena/data/view/GCA_000288275.1) | GB00247 |  |  | 24 |
| 1452 | [GCA_000288395.1](https://www.ebi.ac.uk/ena/data/view/GCA_000288395.1) | GB00911 |  |  | 452 |
| 1455 | [GCA_000310385.1](https://www.ebi.ac.uk/ena/data/view/GCA_000310385.1) | MRI Z1-219 |  |  | 590 |
| 1474 | Mehershahi et al. (2015) | SG-M1 | Asia | 2015 | 283 |
| 1475 | Teatero et al. (2014) | NGBS10 | North America | 2009 | 1 |
| 1476 | Teatero et al. (2014) | NGBS107 | North America | 2010 | 1 |
| 1477 | Teatero et al. (2014) | NGBS110 | North America | 2010 | 1 |
| 1478 | Teatero et al. (2014) | NGBS117 | North America | 2010 | 1 |
| 1484 | Teatero et al. (2014) | NGBS180 | North America | 2010 | 1 |
| 1485 | Teatero et al. (2014) | NGBS200 | North America | 2010 | 1 |
| 1486 | Teatero et al. (2014) | NGBS21 | North America | 2009 | 1 |
| 1488 | Teatero et al. (2014) | NGBS22 | North America | 2009 | 1 |
| 1490 | Teatero et al. (2014) | NGBS234 | North America | 2010 |  |
| 1492 | Teatero et al. (2014) | NGBS244 | North America | 2011 | 871 |
| 1494 | Teatero et al. (2014) | NGBS25 | North America | 2009 | 1 |
| 1495 | Teatero et al. (2014) | NGBS267 | North America | 2010 | 1 |
| 1498 | Teatero et al. (2014) | NGBS275 | North America | 2010 | 1 |
| 1499 | Teatero et al. (2014) | NGBS279 | North America | 2010 | 1 |
| 1500 | Teatero et al. (2014) | NGBS28 | North America | 2009 | 1 |
| 1501 | Teatero et al. (2014) | NGBS283 | North America | 2010 | 1 |
| 1502 | Teatero et al. (2014) | NGBS287 | North America | 2010 | 1 |
| 1503 | Teatero et al. (2014) | NGBS288 | North America | 2010 | 1 |
| 1505 | Teatero et al. (2014) | NGBS30 | North America | 2010 | 1 |
| 1506 | Teatero et al. (2014) | NGBS303 | North America | 2010 | 1 |
| 1515 | Teatero et al. (2014) | NGBS35 | North America | 2010 | 1 |
| 1535 | Teatero et al. (2014) | NGBS54 | North America | 2010 | 1 |
| 1545 | Teatero et al. (2014) | NGBS63 | North America | 2010 | 1 |
| 1547 | Teatero et al. (2014) | NGBS633 | North America | 2012 | 531 |
| 1548 | Teatero et al. (2014) | NGBS68 | North America | 2010 | 1 |
| 1549 | Teatero et al. (2014) | NGBS8 | North America | 2009 | 1 |
| 1550 | Teatero et al. (2014) | NGBS9 | North America | 2010 | 1 |
| 1551 | Teatero et al. (2014) | NGBS92 | North America | 2010 | 1 |
| 1552 | Teatero et al. (2014) | NGBS93 | North America | 2010 | 1 |
| 1553 | Teatero et al. (2014) | NGBS94 | North America | 2010 | 1 |
| 1554 | Teatero et al. (2014) | NGBS99 | North America | 2010 | 1 |
| 1555 | Teatero et al. (2014) | SS100 | North America | 2006 | 1 |
| 1556 | Teatero et al. (2014) | SS102 | North America | 2006 | 1 |
| 1557 | Teatero et al. (2014) | SS103 | North America | 2006 | 1 |
| 1558 | Teatero et al. (2014) | SS104 | North America | 2006 | 1 |
| 1559 | Teatero et al. (2014) | SS105 | North America | 2006 |  |
| 1560 | Teatero et al. (2014) | SS106 | North America | 2006 | 1 |
| 1561 | Teatero et al. (2014) | SS107 | North America | 2006 | 1 |
| 1562 | Teatero et al. (2014) | SS108 | North America | 2006 | 872 |
| 1563 | Teatero et al. (2014) | SS109 | North America | 2006 | 1 |
| 1564 | Teatero et al. (2014) | SS110 | North America | 2007 | 1 |
| 1565 | Teatero et al. (2014) | SS111 | North America | 2007 | 1 |
| 1566 | Teatero et al. (2014) | SS114 | North America | 2007 | 1 |
| 1567 | Teatero et al. (2014) | SS115 | North America | 2007 | 1 |
| 1568 | Teatero et al. (2014) | SS116 | North America | 2007 | 1 |
| 1569 | Teatero et al. (2014) | SS118 | North America | 2008 |  |
| 1570 | Teatero et al. (2014) | SS119 | North America | 2008 | 1 |
| 1571 | Teatero et al. (2014) | SS120 | North America | 2008 | 1 |
| 1572 | Teatero et al. (2014) | SS122 | North America | 2009 | 1 |
| 1573 | Teatero et al. (2014) | SS125 | North America | 2009 | 1 |
| 1574 | Teatero et al. (2014) | SS126 | North America | 2009 | 1 |
| 1575 | Teatero et al. (2014) | SS127 | North America | 2009 | 1 |
| 1576 | Teatero et al. (2014) | SS129 | North America | 2009 | 1 |
| 1577 | Teatero et al. (2014) | SS132 | North America | 2009 | 1 |
| 1578 | Teatero et al. (2014) | SS133 | North America | 2009 | 1 |
| 1579 | Teatero et al. (2014) | SS135 | North America | 2007 | 1 |
| 1580 | Teatero et al. (2014) | SS136 | North America | 2007 | 297 |
| 1581 | Teatero et al. (2014) | SS138 | North America | 2010 | 1 |
| 1582 | Teatero et al. (2014) | SS140 | North America | 2008 | 1 |
| 1583 | Teatero et al. (2014) | SS141 | North America | 2009 | 1 |
| 1584 | Teatero et al. (2014) | SS143 | North America | 2010 | 1 |
| 1585 | Teatero et al. (2014) | SS144 | North America | 2010 | 1 |
| 1586 | Teatero et al. (2014) | SS145 | North America | 2010 | 1 |
| 1587 | Teatero et al. (2014) | SS146 | North America | 2010 | 1 |
| 1588 | Teatero et al. (2014) | SS147 | North America | 2010 | 1 |
| 1593 | Teatero et al. (2014) | SS31 | North America | 1992 | 1 |
| 1594 | Teatero et al. (2014) | SS32 | North America | 1992 | 1 |
| 1595 | Teatero et al. (2014) | SS33 | North America | 1994 | 1 |
| 1596 | Teatero et al. (2014) | SS34 | North America | 1994 | 1 |
| 1597 | Teatero et al. (2014) | SS35 | North America | 1994 | 1 |
| 1598 | Teatero et al. (2014) | SS36 | North America | 1997 | 1 |
| 1599 | Teatero et al. (2014) | SS37 | North America | 1997 | 1 |
| 1600 | Teatero et al. (2014) | SS38 | North America | 1998 | 1 |
| 1601 | Teatero et al. (2014) | SS39 | North America | 1998 | 1 |
| 1602 | Teatero et al. (2014) | SS40 | North America | 1998 | 1 |
| 1603 | Teatero et al. (2014) | SS41 | North America | 1998 | 1 |
| 1604 | Teatero et al. (2014) | SS42 | North America | 1998 | 1 |
| 1605 | Teatero et al. (2014) | SS43 | North America | 1998 | 1 |
| 1606 | Teatero et al. (2014) | SS44 | North America | 1999 | 1 |
| 1607 | Teatero et al. (2014) | SS45 | North America | 1999 | 1 |
| 1608 | Teatero et al. (2014) | SS46 | North America | 1999 | 1 |
| 1609 | Teatero et al. (2014) | SS47 | North America | 2000 | 1 |
| 1610 | Teatero et al. (2014) | SS48 | North America | 2000 | 1 |
| 1611 | Teatero et al. (2014) | SS49 | North America | 2000 | 873 |
| 1612 | Teatero et al. (2014) | SS50 | North America | 2000 | 1 |
| 1613 | Teatero et al. (2014) | SS51 | North America | 2000 | 1 |
| 1614 | Teatero et al. (2014) | SS52 | North America | 2000 | 1 |
| 1615 | Teatero et al. (2014) | SS53 | North America | 2000 | 1 |
| 1616 | Teatero et al. (2014) | SS54 | North America | 2001 | 1 |
| 1617 | Teatero et al. (2014) | SS56 | North America | 2001 | 1 |
| 1618 | Teatero et al. (2014) | SS57 | North America | 2001 | 1 |
| 1619 | Teatero et al. (2014) | SS58 | North America | 2001 | 1 |
| 1620 | Teatero et al. (2014) | SS59 | North America | 2001 | 1 |
| 1621 | Teatero et al. (2014) | SS60 | North America | 2001 | 1 |
| 1622 | Teatero et al. (2014) | SS61 | North America | 2002 | 1 |
| 1623 | Teatero et al. (2014) | SS62 | North America | 2002 | 1 |
| 1624 | Teatero et al. (2014) | SS63 | North America | 2002 | 1 |
| 1625 | Teatero et al. (2014) | SS64 | North America | 2002 | 1 |
| 1626 | Teatero et al. (2014) | SS65 | North America | 2002 | 1 |
| 1627 | Teatero et al. (2014) | SS66 | North America | 2002 | 1 |
| 1628 | Teatero et al. (2014) | SS67 | North America | 2002 | 1 |
| 1629 | Teatero et al. (2014) | SS68 | North America | 2003 | 1 |
| 1630 | Teatero et al. (2014) | SS69 | North America | 2003 | 1 |
| 1631 | Teatero et al. (2014) | SS70 | North America | 2003 |  |
| 1632 | Teatero et al. (2014) | SS71 | North America | 2003 | 1 |
| 1633 | Teatero et al. (2014) | SS72 | North America | 2003 | 1 |
| 1634 | Teatero et al. (2014) | SS74 | North America | 2004 | 1 |
| 1635 | Teatero et al. (2014) | SS75 | North America | 2004 | 1 |
| 1636 | Teatero et al. (2014) | SS76 | North America | 2004 | 1 |
| 1637 | Teatero et al. (2014) | SS77 | North America | 2004 | 1 |
| 1638 | Teatero et al. (2014) | SS78 | North America | 2004 | 1 |
| 1639 | Teatero et al. (2014) | SS79 | North America | 2004 | 153 |
| 1640 | Teatero et al. (2014) | SS80 | North America | 2004 | 1 |
| 1641 | Teatero et al. (2014) | SS81 | North America | 2004 | 1 |
| 1642 | Teatero et al. (2014) | SS82 | North America | 2004 | 1 |
| 1643 | Teatero et al. (2014) | SS83 | North America | 2004 | 1 |
| 1644 | Teatero et al. (2014) | SS84 | North America | 2005 | 1 |
| 1645 | Teatero et al. (2014) | SS85 | North America | 2005 | 1 |
| 1646 | Teatero et al. (2014) | SS86 | North America | 2005 | 1 |
| 1647 | Teatero et al. (2014) | SS87 | North America | 2005 | 1 |
| 1648 | Teatero et al. (2014) | SS88 | North America | 2005 | 1 |
| 1649 | Teatero et al. (2014) | SS89 | North America | 2005 | 1 |
| 1650 | Teatero et al. (2014) | SS92 | North America | 2005 | 1 |
| 1651 | Teatero et al. (2014) | SS93 | North America | 2005 | 1 |
| 1652 | Teatero et al. (2014) | SS94 | North America | 2005 | 1 |
| 1653 | Teatero et al. (2014) | SS95 | North America | 2005 | 1 |
| 1654 | Teatero et al. (2014) | SS96 | North America | 2005 | 1 |
| 1655 | Teatero et al. (2014) | SS97 | North America | 2005 | 1 |
| 1656 | Teatero et al. (2014) | SS98 | North America | 2005 | 1 |
| 1657 | Teatero et al. (2014) | NGBS003 | North America | 2009 | 2 |
| 1658 | Teatero et al. (2014) | NGBS050 | North America | 2010 | 17 |
| 1659 | Teatero et al. (2014) | NGBS069 | North America | 2010 | 17 |
| 1661 | Teatero et al. (2014) | NGBS079 | North America | 2010 | 17 |
| 1662 | Teatero et al. (2014) | NGBS082 | North America | 2010 | 17 |
| 1663 | Teatero et al. (2014) | NGBS106 | North America | 2010 |  |
| 1665 | Teatero et al. (2014) | NGBS128 | North America | 2010 | 17 |
| 1666 | Teatero et al. (2014) | NGBS169 | North America | 2010 | 17 |
| 1667 | Teatero et al. (2014) | NGBS186 | North America | 2010 | 17 |
| 1670 | Teatero et al. (2014) | NGBS222 | North America | 2010 | 17 |
| 1673 | Teatero et al. (2014) | NGBS250 | North America | 2011 | 17 |
| 1674 | Teatero et al. (2014) | NGBS271 | North America | 2011 |  |
| 1675 | Teatero et al. (2014) | NGBS277 | North America | 2010 | 17 |
| 1676 | Teatero et al. (2014) | NGBS282 | North America | 2010 | 17 |
| 1677 | Teatero et al. (2014) | NGBS291 | North America | 2010 | 17 |
| 1678 | Teatero et al. (2014) | NGBS296 | North America | 2010 | 17 |
| 1679 | Teatero et al. (2014) | NGBS297 | North America | 2010 | 17 |
| 1683 | Teatero et al. (2014) | NGBS318 | North America | 2011 | 290 |
| 1684 | Teatero et al. (2014) | NGBS327 | North America | 2011 | 484 |
| 1686 | Teatero et al. (2014) | NGBS345 | North America | 2011 | 874 |
| 1711 | Teatero et al. (2014) | NGBS502 | North America | 2012 | 95 |
| 1714 | Teatero et al. (2014) | NGBS531 | North America | 2012 | 148 |
| 1722 | Teatero et al. (2014) | NGBS608 | North America | 2012 | 17 |
| 1727 | Teatero et al. (2014) | NGBS622 | North America | 2012 | 148 |
| 1730 | Teatero et al. (2014) | NGBS632 | North America | 2012 | 17 |
| 1731 | Teatero et al. (2014) | NGBS636 | North America | 2010 | 17 |
| 1732 | Teatero et al. (2014) | NGBS641 | North America | 2010 | 17 |
| 1733 | Teatero et al. (2014) | NGBS644 | North America | 2011 | 17 |
| 1734 | Teatero et al. (2014) | NGBS650 | North America | 2011 | 17 |
| 1735 | Teatero et al. (2014) | NGBS44 | North America | 2010 | 1 |
| 1897 | Seale et al. (2016) | K59213 | Africa | 2012 | 1 |
| 1898 | Seale et al. (2016) | K58722 | Africa | 2012 | 182 |
| 1899 | Seale et al. (2016) | K56677 | Africa | 2012 | 1 |
| 1900 | Seale et al. (2016) | K62205 | Africa | 2013 | 23 |
| 1901 | Seale et al. (2016) | K61856 | Africa | 2013 | 23 |
| 1902 | Seale et al. (2016) | K61593 | Africa | 2013 | 1 |
| 1903 | Seale et al. (2016) | K61569 | Africa | 2013 | 10 |
| 1904 | Seale et al. (2016) | K61520 | Africa | 2013 | 484 |
| 1905 | Seale et al. (2016) | K61337 | Africa | 2013 | 8 |
| 1906 | Seale et al. (2016) | K61281 | Africa | 2013 | 1 |
| 1907 | Seale et al. (2016) | K61004 | Africa | 2013 | 17 |
| 1908 | Seale et al. (2016) | K60927 | Africa | 2013 | 23 |
| 1909 | Seale et al. (2016) | K60926 | Africa | 2013 | 23 |
| 1910 | Seale et al. (2016) | K60802 | Africa | 2013 | 28 |
| 1911 | Seale et al. (2016) | K60691 | Africa | 2013 | 484 |
| 1912 | Seale et al. (2016) | K59750 | Africa | 2012 | 484 |
| 1913 | Seale et al. (2016) | K59732 | Africa | 2012 | 17 |
| 1914 | Seale et al. (2016) | K59579 | Africa | 2012 | 8 |
| 1915 | Seale et al. (2016) | K59519 | Africa | 2012 | 1 |
| 1916 | Seale et al. (2016) | K59497 | Africa | 2012 | 17 |
| 1917 | Seale et al. (2016) | K59496 | Africa | 2012 | 10 |
| 1918 | Seale et al. (2016) | K59380 | Africa | 2012 | 182 |
| 1919 | Seale et al. (2016) | K59319 | Africa | 2012 | 1 |
| 1920 | Seale et al. (2016) | K59216 | Africa | 2012 | 17 |
| 1921 | Seale et al. (2016) | K59112 | Africa | 2012 | 1 |
| 1922 | Seale et al. (2016) | K58953 | Africa | 2012 | 1 |
| 1923 | Seale et al. (2016) | K58904 | Africa | 2012 | 1 |
| 1924 | Seale et al. (2016) | K58847 | Africa | 2012 | 1 |
| 1925 | Seale et al. (2016) | K58834 | Africa | 2012 | 23 |
| 1926 | Seale et al. (2016) | K58784 | Africa | 2012 | 17 |
| 1927 | Seale et al. (2016) | K58759 | Africa | 2012 | 484 |
| 1928 | Seale et al. (2016) | K58245 | Africa | 2012 | 17 |
| 1929 | Seale et al. (2016) | K58244 | Africa | 2012 | 10 |
| 1930 | Seale et al. (2016) | K57971 | Africa | 2012 | 10 |
| 1931 | Seale et al. (2016) | K57970 | Africa | 2012 | 8 |
| 1932 | Seale et al. (2016) | K57879 | Africa | 2012 | 8 |
| 1933 | Seale et al. (2016) | K57791 | Africa | 2012 | 10 |
| 1934 | Seale et al. (2016) | K57354 | Africa | 2012 | 17 |
| 1935 | Seale et al. (2016) | K57353 | Africa | 2012 | 17 |
| 1936 | Seale et al. (2016) | K57172 | Africa | 2012 | 14 |
| 1937 | Seale et al. (2016) | K57063 | Africa | 2012 | 17 |
| 1938 | Seale et al. (2016) | K56978 | Africa | 2012 | 182 |
| 1939 | Seale et al. (2016) | K56941 | Africa | 2012 | 17 |
| 1940 | Seale et al. (2016) | K56795 | Africa | 2012 | 23 |
| 1941 | Seale et al. (2016) | K56485 | Africa | 2012 | 786 |
| 1942 | Seale et al. (2016) | K56441 | Africa | 2012 | 23 |
| 1943 | Seale et al. (2016) | K56376 | Africa | 2012 | 23 |
| 1952 | Seale et al. (2016) | K57091 | Africa |  | 327 |
| 1961 | Seale et al. (2016) | K62585 | Africa | 2013 | 23 |
| 1962 | Seale et al. (2016) | K62584 | Africa | 2013 | 19 |
| 1963 | Seale et al. (2016) | K62583 | Africa | 2013 | 17 |
| 1964 | Seale et al. (2016) | K62582 | Africa | 2013 | 17 |
| 1965 | Seale et al. (2016) | K62581 | Africa | 2013 | 10 |
| 1966 | Seale et al. (2016) | K62580 | Africa | 2013 | 23 |
| 1967 | Seale et al. (2016) | K62570 | Africa | 2013 | 23 |
| 1968 | Seale et al. (2016) | K62569 | Africa | 2013 | 17 |
| 1969 | Seale et al. (2016) | K62567 | Africa | 2013 | 23 |
| 1970 | Seale et al. (2016) | K62558 | Africa | 2013 | 291 |
| 1971 | Seale et al. (2016) | K62557 | Africa | 2013 | 19 |
| 1972 | Seale et al. (2016) | K62556 | Africa | 2013 | 23 |
| 1973 | Seale et al. (2016) | K62554 | Africa | 2013 | 17 |
| 1974 | Seale et al. (2016) | K62534 | Africa | 2013 | 23 |
| 1975 | Seale et al. (2016) | K62476 | Africa | 2013 | 17 |
| 1976 | Seale et al. (2016) | K62475 | Africa | 2013 | 484 |
| 1977 | Seale et al. (2016) | K62474 | Africa | 2013 | 17 |
| 1978 | Seale et al. (2016) | K62464 | Africa | 2013 | 17 |
| 1979 | Seale et al. (2016) | K62463 | Africa | 2013 | 10 |
| 1980 | Seale et al. (2016) | K62462 | Africa | 2013 | 10 |
| 1981 | Seale et al. (2016) | K62461 | Africa | 2013 | 23 |
| 1982 | Seale et al. (2016) | K62460 | Africa | 2013 | 1 |
| 1983 | Seale et al. (2016) | K62459 | Africa | 2013 | 484 |
| 1984 | Seale et al. (2016) | K62446 | Africa | 2013 | 23 |
| 1985 | Seale et al. (2016) | K62445 | Africa | 2013 | 23 |
| 1986 | Seale et al. (2016) | K62444 | Africa | 2013 | 23 |
| 1987 | Seale et al. (2016) | K62419 | Africa | 2013 | 23 |
| 1988 | Seale et al. (2016) | K62418 | Africa | 2013 | 17 |
| 1989 | Seale et al. (2016) | K62393 | Africa | 2013 | 17 |
| 1990 | Seale et al. (2016) | K62392 | Africa | 2013 | 328 |
| 1991 | Seale et al. (2016) | K62350 | Africa | 2013 | 10 |
| 1992 | Seale et al. (2016) | K62349 | Africa | 2013 | 484 |
| 1993 | Seale et al. (2016) | K62348 | Africa | 2013 | 17 |
| 1994 | Seale et al. (2016) | K62347 | Africa | 2013 | 19 |
| 1995 | Seale et al. (2016) | K62313 | Africa | 2013 | 17 |
| 1996 | Seale et al. (2016) | K62312 | Africa | 2013 | 23 |
| 1997 | Seale et al. (2016) | K62311 | Africa | 2013 | 1 |
| 1998 | Seale et al. (2016) | K62252 | Africa | 2013 | 19 |
| 1999 | Seale et al. (2016) | K62251 | Africa | 2013 | 182 |
| 2000 | Seale et al. (2016) | K62250 | Africa | 2013 | 484 |
| 2001 | Seale et al. (2016) | K62249 | Africa | 2013 | 327 |
| 2002 | Seale et al. (2016) | K62210 | Africa | 2013 | 1 |
| 2003 | Seale et al. (2016) | K62209 | Africa | 2013 | 3 |
| 2004 | Seale et al. (2016) | K62208 | Africa | 2013 | 1 |
| 2005 | Seale et al. (2016) | K62207 | Africa | 2013 | 19 |
| 2006 | Seale et al. (2016) | K62204 | Africa | 2013 | 23 |
| 2007 | Seale et al. (2016) | K62170 | Africa | 2013 | 8 |
| 2008 | Seale et al. (2016) | K62168 | Africa | 2013 | 17 |
| 2009 | Seale et al. (2016) | K62167 | Africa | 2013 | 1 |
| 2010 | Seale et al. (2016) | K62165 | Africa | 2013 | 1 |
| 2011 | Seale et al. (2016) | K62164 | Africa | 2013 | 19 |
| 2012 | Seale et al. (2016) | K62157 | Africa | 2013 | 103 |
| 2013 | Seale et al. (2016) | K62156 | Africa | 2013 | 17 |
| 2014 | Seale et al. (2016) | K62155 | Africa | 2013 | 1 |
| 2015 | Seale et al. (2016) | K62154 | Africa | 2013 | 17 |
| 2016 | Seale et al. (2016) | K62153 | Africa | 2013 | 17 |
| 2017 | Seale et al. (2016) | K62152 | Africa | 2013 | 23 |
| 2018 | Seale et al. (2016) | K62151 | Africa | 2013 | 8 |
| 2019 | Seale et al. (2016) | K62145 | Africa | 2013 | 801 |
| 2020 | Seale et al. (2016) | K62144 | Africa | 2013 | 1 |
| 2021 | Seale et al. (2016) | K62143 | Africa | 2013 | 484 |
| 2022 | Seale et al. (2016) | K62142 | Africa | 2013 | 484 |
| 2023 | Seale et al. (2016) | K62111 | Africa | 2013 | 1 |
| 2024 | Seale et al. (2016) | K62110 | Africa | 2013 | 17 |
| 2025 | Seale et al. (2016) | K62080 | Africa | 2013 | 17 |
| 2026 | Seale et al. (2016) | K62079 | Africa | 2013 | 17 |
| 2027 | Seale et al. (2016) | K62064 | Africa | 2013 | 10 |
| 2028 | Seale et al. (2016) | K62063 | Africa | 2013 | 182 |
| 2029 | Seale et al. (2016) | K62062 | Africa | 2013 | 23 |
| 2030 | Seale et al. (2016) | K62044 | Africa | 2013 | 1 |
| 2031 | Seale et al. (2016) | K62043 | Africa | 2013 | 17 |
| 2032 | Seale et al. (2016) | K62042 | Africa | 2013 | 17 |
| 2033 | Seale et al. (2016) | K62032 | Africa | 2013 | 17 |
| 2034 | Seale et al. (2016) | K62031 | Africa | 2013 | 10 |
| 2035 | Seale et al. (2016) | K62030 | Africa | 2013 | 10 |
| 2036 | Seale et al. (2016) | K62029 | Africa | 2013 | 28 |
| 2037 | Seale et al. (2016) | K62026 | Africa | 2013 | 10 |
| 2038 | Seale et al. (2016) | K62025 | Africa | 2013 | 17 |
| 2039 | Seale et al. (2016) | K61994 | Africa | 2013 | 17 |
| 2040 | Seale et al. (2016) | K61855 | Africa | 2013 | 327 |
| 2041 | Seale et al. (2016) | K61845 | Africa | 2013 | 17 |
| 2042 | Seale et al. (2016) | K61844 | Africa | 2013 | 17 |
| 2043 | Seale et al. (2016) | K61843 | Africa | 2013 | 19 |
| 2044 | Seale et al. (2016) | K61842 | Africa | 2013 | 10 |
| 2045 | Seale et al. (2016) | K61841 | Africa | 2013 | 17 |
| 2046 | Seale et al. (2016) | K61840 | Africa | 2013 | 17 |
| 2047 | Seale et al. (2016) | K61832 | Africa | 2013 | 17 |
| 2048 | Seale et al. (2016) | K61823 | Africa | 2013 | 19 |
| 2049 | Seale et al. (2016) | K61781 | Africa | 2013 | 10 |
| 2050 | Seale et al. (2016) | K61780 | Africa | 2013 | 23 |
| 2051 | Seale et al. (2016) | K61773 | Africa | 2013 | 28 |
| 2052 | Seale et al. (2016) | K61772 | Africa | 2013 | 23 |
| 2053 | Seale et al. (2016) | K61771 | Africa | 2013 | 23 |
| 2054 | Seale et al. (2016) | K61768 | Africa | 2013 | 23 |
| 2055 | Seale et al. (2016) | K61767 | Africa | 2013 | 484 |
| 2056 | Seale et al. (2016) | K61766 | Africa | 2013 | 12 |
| 2057 | Seale et al. (2016) | K61765 | Africa | 2013 | 12 |
| 2058 | Seale et al. (2016) | K61764 | Africa | 2013 | 10 |
| 2059 | Seale et al. (2016) | K61763 | Africa | 2013 | 17 |
| 2060 | Seale et al. (2016) | K61749 | Africa | 2013 | 28 |
| 2061 | Seale et al. (2016) | K61748 | Africa | 2013 | 182 |
| 2062 | Seale et al. (2016) | K61747 | Africa | 2013 | 23 |
| 2063 | Seale et al. (2016) | K61746 | Africa | 2013 | 23 |
| 2064 | Seale et al. (2016) | K61739 | Africa | 2013 | 23 |
| 2065 | Seale et al. (2016) | K61733 | Africa | 2013 | 23 |
| 2066 | Seale et al. (2016) | K61732 | Africa | 2013 | 1 |
| 2067 | Seale et al. (2016) | K61731 | Africa | 2013 | 17 |
| 2068 | Seale et al. (2016) | K61730 | Africa | 2013 | 23 |
| 2069 | Seale et al. (2016) | K61729 | Africa | 2013 | 19 |
| 2070 | Seale et al. (2016) | K61725 | Africa | 2013 | 28 |
| 2071 | Seale et al. (2016) | K61704 | Africa | 2013 | 484 |
| 2072 | Seale et al. (2016) | K61692 | Africa | 2013 | 28 |
| 2073 | Seale et al. (2016) | K61691 | Africa | 2013 | 23 |
| 2074 | Seale et al. (2016) | K61690 | Africa | 2013 | 1 |
| 2075 | Seale et al. (2016) | K61689 | Africa | 2013 | 23 |
| 2076 | Seale et al. (2016) | K61688 | Africa | 2013 | 109 |
| 2077 | Seale et al. (2016) | K61685 | Africa | 2013 | 1 |
| 2078 | Seale et al. (2016) | K61684 | Africa | 2013 | 182 |
| 2080 | Seale et al. (2016) | K61670 | Africa | 2013 | 1 |
| 2081 | Seale et al. (2016) | K61669 | Africa | 2013 | 23 |
| 2082 | Seale et al. (2016) | K61668 | Africa | 2013 | 23 |
| 2083 | Seale et al. (2016) | K61640 | Africa | 2013 | 17 |
| 2084 | Seale et al. (2016) | K61639 | Africa | 2013 | 17 |
| 2085 | Seale et al. (2016) | K61622 | Africa | 2013 | 10 |
| 2086 | Seale et al. (2016) | K61620 | Africa | 2013 | 17 |
| 2087 | Seale et al. (2016) | K61618 | Africa | 2013 | 10 |
| 2088 | Seale et al. (2016) | K61611 | Africa | 2013 | 17 |
| 2089 | Seale et al. (2016) | K61610 | Africa | 2013 | 23 |
| 2090 | Seale et al. (2016) | K61608 | Africa | 2013 | 17 |
| 2091 | Seale et al. (2016) | K61606 | Africa | 2013 | 28 |
| 2092 | Seale et al. (2016) | K61594 | Africa | 2013 | 484 |
| 2093 | Seale et al. (2016) | K61592 | Africa | 2013 | 10 |
| 2094 | Seale et al. (2016) | K61591 | Africa | 2013 | 2 |
| 2095 | Seale et al. (2016) | K61590 | Africa | 2013 | 17 |
| 2096 | Seale et al. (2016) | K61589 | Africa | 2013 | 798 |
| 2097 | Seale et al. (2016) | K61586 | Africa | 2013 | 484 |
| 2098 | Seale et al. (2016) | K61572 | Africa | 2013 | 17 |
| 2099 | Seale et al. (2016) | K61571 | Africa | 2013 | 23 |
| 2100 | Seale et al. (2016) | K61533 | Africa | 2013 | 10 |
| 2101 | Seale et al. (2016) | K61521 | Africa | 2013 | 17 |
| 2102 | Seale et al. (2016) | K61516 | Africa | 2013 | 17 |
| 2103 | Seale et al. (2016) | K61515 | Africa | 2013 | 8 |
| 2104 | Seale et al. (2016) | K61514 | Africa | 2013 | 484 |
| 2105 | Seale et al. (2016) | K61512 | Africa | 2013 | 327 |
| 2106 | Seale et al. (2016) | K61491 | Africa | 2013 |  |
| 2107 | Seale et al. (2016) | K61481 | Africa | 2013 | 327 |
| 2108 | Seale et al. (2016) | K61465 | Africa | 2013 | 182 |
| 2109 | Seale et al. (2016) | K61464 | Africa | 2013 | 797 |
| 2110 | Seale et al. (2016) | K61463 | Africa | 2013 | 10 |
| 2111 | Seale et al. (2016) | K61461 | Africa | 2013 | 484 |
| 2112 | Seale et al. (2016) | K61460 | Africa | 2013 | 182 |
| 2113 | Seale et al. (2016) | K61459 | Africa | 2013 | 23 |
| 2114 | Seale et al. (2016) | K61457 | Africa | 2013 | 10 |
| 2115 | Seale et al. (2016) | K61438 | Africa | 2013 | 23 |
| 2116 | Seale et al. (2016) | K61437 | Africa | 2013 | 8 |
| 2117 | Seale et al. (2016) | K61436 | Africa | 2013 | 10 |
| 2118 | Seale et al. (2016) | K61435 | Africa | 2013 | 17 |
| 2119 | Seale et al. (2016) | K61434 | Africa | 2013 | 1 |
| 2120 | Seale et al. (2016) | K61432 | Africa | 2013 | 10 |
| 2121 | Seale et al. (2016) | K61431 | Africa | 2013 | 790 |
| 2122 | Seale et al. (2016) | K61428 | Africa | 2013 | 23 |
| 2123 | Seale et al. (2016) | K61416 | Africa | 2013 | 10 |
| 2124 | Seale et al. (2016) | K61415 | Africa | 2013 | 484 |
| 2125 | Seale et al. (2016) | K61414 | Africa | 2013 | 23 |
| 2126 | Seale et al. (2016) | K61407 | Africa | 2013 | 484 |
| 2127 | Seale et al. (2016) | K61395 | Africa | 2013 | 23 |
| 2128 | Seale et al. (2016) | K61394 | Africa | 2013 | 23 |
| 2129 | Seale et al. (2016) | K61381 | Africa | 2013 | 1 |
| 2130 | Seale et al. (2016) | K61379 | Africa | 2013 | 23 |
| 2131 | Seale et al. (2016) | K61378 | Africa | 2013 | 10 |
| 2132 | Seale et al. (2016) | K61363 | Africa | 2013 | 23 |
| 2133 | Seale et al. (2016) | K61362 | Africa | 2013 | 17 |
| 2134 | Seale et al. (2016) | K61361 | Africa | 2013 | 10 |
| 2135 | Seale et al. (2016) | K61360 | Africa | 2013 | 796 |
| 2136 | Seale et al. (2016) | K61359 | Africa | 2013 | 10 |
| 2137 | Seale et al. (2016) | K61342 | Africa | 2013 | 28 |
| 2138 | Seale et al. (2016) | K61341 | Africa | 2013 | 24 |
| 2139 | Seale et al. (2016) | K61336 | Africa | 2013 | 484 |
| 2140 | Seale et al. (2016) | K61335 | Africa | 2013 | 17 |
| 2141 | Seale et al. (2016) | K61334 | Africa | 2013 | 23 |
| 2142 | Seale et al. (2016) | K61331 | Africa | 2013 | 17 |
| 2143 | Seale et al. (2016) | K61330 | Africa | 2013 | 8 |
| 2144 | Seale et al. (2016) | K61329 | Africa | 2013 | 10 |
| 2145 | Seale et al. (2016) | K61328 | Africa | 2013 | 23 |
| 2146 | Seale et al. (2016) | K61308 | Africa | 2013 | 328 |
| 2147 | Seale et al. (2016) | K61307 | Africa | 2013 | 196 |
| 2148 | Seale et al. (2016) | K61306 | Africa | 2013 | 8 |
| 2149 | Seale et al. (2016) | K61305 | Africa | 2013 | 17 |
| 2150 | Seale et al. (2016) | K61304 | Africa | 2013 | 23 |
| 2151 | Seale et al. (2016) | K61282 | Africa | 2013 | 17 |
| 2152 | Seale et al. (2016) | K61280 | Africa | 2013 | 484 |
| 2153 | Seale et al. (2016) | K61279 | Africa | 2013 | 1 |
| 2154 | Seale et al. (2016) | K61278 | Africa | 2013 | 23 |
| 2155 | Seale et al. (2016) | K61276 | Africa | 2013 | 17 |
| 2156 | Seale et al. (2016) | K61270 | Africa | 2013 | 19 |
| 2157 | Seale et al. (2016) | K61269 | Africa | 2013 | 23 |
| 2158 | Seale et al. (2016) | K61265 | Africa | 2013 | 8 |
| 2159 | Seale et al. (2016) | K61263 | Africa | 2013 | 1 |
| 2160 | Seale et al. (2016) | K61261 | Africa | 2013 | 2 |
| 2161 | Seale et al. (2016) | K61259 | Africa | 2013 | 8 |
| 2162 | Seale et al. (2016) | K61258 | Africa | 2013 | 23 |
| 2163 | Seale et al. (2016) | K61256 | Africa | 2013 | 8 |
| 2164 | Seale et al. (2016) | K61255 | Africa | 2013 | 182 |
| 2165 | Seale et al. (2016) | K61252 | Africa | 2013 | 484 |
| 2166 | Seale et al. (2016) | K61251 | Africa | 2013 | 17 |
| 2167 | Seale et al. (2016) | K61250 | Africa | 2013 | 182 |
| 2168 | Seale et al. (2016) | K61236 | Africa | 2013 | 1 |
| 2169 | Seale et al. (2016) | K61235 | Africa | 2013 | 2 |
| 2170 | Seale et al. (2016) | K61223 | Africa | 2013 | 23 |
| 2171 | Seale et al. (2016) | K61219 | Africa | 2013 | 2 |
| 2172 | Seale et al. (2016) | K61211 | Africa | 2013 | 23 |
| 2173 | Seale et al. (2016) | K61196 | Africa | 2013 | 23 |
| 2174 | Seale et al. (2016) | K61194 | Africa | 2013 | 23 |
| 2175 | Seale et al. (2016) | K61193 | Africa | 2013 | 24 |
| 2176 | Seale et al. (2016) | K61192 | Africa | 2013 | 182 |
| 2177 | Seale et al. (2016) | K61191 | Africa | 2013 | 23 |
| 2178 | Seale et al. (2016) | K61177 | Africa | 2013 | 484 |
| 2179 | Seale et al. (2016) | K61175 | Africa | 2013 | 23 |
| 2180 | Seale et al. (2016) | K61164 | Africa | 2013 | 484 |
| 2181 | Seale et al. (2016) | K61163 | Africa | 2013 | 328 |
| 2182 | Seale et al. (2016) | K61143 | Africa | 2013 | 196 |
| 2183 | Seale et al. (2016) | K61142 | Africa | 2013 | 196 |
| 2184 | Seale et al. (2016) | K61141 | Africa | 2013 | 10 |
| 2185 | Seale et al. (2016) | K61138 | Africa | 2013 |  |
| 2186 | Seale et al. (2016) | K61119 | Africa | 2013 | 182 |
| 2187 | Seale et al. (2016) | K61118 | Africa | 2013 | 182 |
| 2188 | Seale et al. (2016) | K61116 | Africa | 2013 | 28 |
| 2189 | Seale et al. (2016) | K61115 | Africa | 2013 | 8 |
| 2190 | Seale et al. (2016) | K61079 | Africa | 2013 | 17 |
| 2191 | Seale et al. (2016) | K61078 | Africa | 2013 | 17 |
| 2192 | Seale et al. (2016) | K61069 | Africa | 2013 | 223 |
| 2193 | Seale et al. (2016) | K61068 | Africa | 2013 | 484 |
| 2194 | Seale et al. (2016) | K61067 | Africa | 2013 | 182 |
| 2195 | Seale et al. (2016) | K61066 | Africa | 2013 | 23 |
| 2196 | Seale et al. (2016) | K61063 | Africa | 2013 | 17 |
| 2197 | Seale et al. (2016) | K61062 | Africa | 2013 | 23 |
| 2198 | Seale et al. (2016) | K61061 | Africa | 2013 | 1 |
| 2199 | Seale et al. (2016) | K61060 | Africa | 2013 | 1 |
| 2200 | Seale et al. (2016) | K61053 | Africa | 2013 | 484 |
| 2201 | Seale et al. (2016) | K61035 | Africa | 2013 | 23 |
| 2202 | Seale et al. (2016) | K61034 | Africa | 2013 | 23 |
| 2203 | Seale et al. (2016) | K61018 | Africa | 2013 | 484 |
| 2204 | Seale et al. (2016) | K61017 | Africa | 2013 | 794 |
| 2205 | Seale et al. (2016) | K61016 | Africa | 2013 | 17 |
| 2206 | Seale et al. (2016) | K61008 | Africa | 2013 | 8 |
| 2207 | Seale et al. (2016) | K61006 | Africa | 2013 | 17 |
| 2208 | Seale et al. (2016) | K61005 | Africa | 2013 | 23 |
| 2209 | Seale et al. (2016) | K61000 | Africa | 2013 | 2 |
| 2210 | Seale et al. (2016) | K60999 | Africa | 2013 | 1 |
| 2211 | Seale et al. (2016) | K60994 | Africa | 2013 | 23 |
| 2212 | Seale et al. (2016) | K60992 | Africa | 2013 | 23 |
| 2213 | Seale et al. (2016) | K60961 | Africa | 2013 | 28 |
| 2214 | Seale et al. (2016) | K60957 | Africa | 2013 | 17 |
| 2216 | Seale et al. (2016) | K60939 | Africa | 2013 | 17 |
| 2217 | Seale et al. (2016) | K60937 | Africa | 2013 | 10 |
| 2218 | Seale et al. (2016) | K60929 | Africa | 2013 | 802 |
| 2219 | Seale et al. (2016) | K60928 | Africa | 2013 | 10 |
| 2220 | Seale et al. (2016) | K60905 | Africa | 2013 | 182 |
| 2221 | Seale et al. (2016) | K60903 | Africa | 2013 | 10 |
| 2222 | Seale et al. (2016) | K60902 | Africa | 2013 | 28 |
| 2223 | Seale et al. (2016) | K60901 | Africa | 2013 |  |
| 2224 | Seale et al. (2016) | K60888 | Africa | 2013 | 23 |
| 2225 | Seale et al. (2016) | K60887 | Africa | 2013 | 17 |
| 2226 | Seale et al. (2016) | K60886 | Africa | 2013 | 23 |
| 2227 | Seale et al. (2016) | K60885 | Africa | 2013 | 17 |
| 2228 | Seale et al. (2016) | K60881 | Africa | 2013 | 8 |
| 2229 | Seale et al. (2016) | K60880 | Africa | 2013 | 8 |
| 2230 | Seale et al. (2016) | K60878 | Africa | 2013 | 1 |
| 2231 | Seale et al. (2016) | K60877 | Africa | 2013 | 799 |
| 2232 | Seale et al. (2016) | K60876 | Africa | 2013 | 23 |
| 2233 | Seale et al. (2016) | K60871 | Africa | 2013 | 23 |
| 2234 | Seale et al. (2016) | K60870 | Africa | 2013 | 196 |
| 2235 | Seale et al. (2016) | K60844 | Africa | 2013 | 23 |
| 2236 | Seale et al. (2016) | K60843 | Africa | 2013 | 800 |
| 2237 | Seale et al. (2016) | K60831 | Africa | 2013 | 17 |
| 2238 | Seale et al. (2016) | K60830 | Africa | 2013 | 17 |
| 2239 | Seale et al. (2016) | K60829 | Africa | 2013 | 8 |
| 2240 | Seale et al. (2016) | K60812 | Africa | 2013 | 17 |
| 2241 | Seale et al. (2016) | K60811 | Africa | 2013 | 19 |
| 2242 | Seale et al. (2016) | K60801 | Africa | 2013 | 10 |
| 2243 | Seale et al. (2016) | K60800 | Africa | 2013 | 28 |
| 2244 | Seale et al. (2016) | K60799 | Africa | 2013 | 182 |
| 2245 | Seale et al. (2016) | K60797 | Africa | 2013 | 17 |
| 2246 | Seale et al. (2016) | K60796 | Africa | 2013 | 28 |
| 2247 | Seale et al. (2016) | K60794 | Africa | 2013 | 23 |
| 2248 | Seale et al. (2016) | K60793 | Africa | 2013 | 484 |
| 2249 | Seale et al. (2016) | K60792 | Africa | 2013 |  |
| 2250 | Seale et al. (2016) | K60790 | Africa | 2013 | 17 |
| 2251 | Seale et al. (2016) | K60789 | Africa | 2013 | 327 |
| 2252 | Seale et al. (2016) | K60788 | Africa | 2013 | 17 |
| 2253 | Seale et al. (2016) | K60785 | Africa | 2013 | 8 |
| 2254 | Seale et al. (2016) | K60756 | Africa | 2013 | 17 |
| 2255 | Seale et al. (2016) | K60721 | Africa | 2013 | 182 |
| 2256 | Seale et al. (2016) | K60720 | Africa | 2013 | 17 |
| 2257 | Seale et al. (2016) | K60719 | Africa | 2013 | 17 |
| 2258 | Seale et al. (2016) | K60715 | Africa | 2013 | 484 |
| 2259 | Seale et al. (2016) | K60714 | Africa | 2013 | 23 |
| 2260 | Seale et al. (2016) | K60693 | Africa | 2013 | 799 |
| 2261 | Seale et al. (2016) | K60690 | Africa | 2013 | 1 |
| 2262 | Seale et al. (2016) | K60689 | Africa | 2013 | 24 |
| 2263 | Seale et al. (2016) | K60688 | Africa | 2013 | 10 |
| 2264 | Seale et al. (2016) | K60670 | Africa | 2013 | 17 |
| 2265 | Seale et al. (2016) | K60668 | Africa | 2013 | 10 |
| 2266 | Seale et al. (2016) | K60666 | Africa | 2013 | 1 |
| 2267 | Seale et al. (2016) | K60664 | Africa | 2013 | 23 |
| 2268 | Seale et al. (2016) | K60663 | Africa | 2013 | 17 |
| 2269 | Seale et al. (2016) | K60658 | Africa | 2013 | 23 |
| 2270 | Seale et al. (2016) | K60657 | Africa | 2013 | 796 |
| 2271 | Seale et al. (2016) | K60577 | Africa | 2013 | 23 |
| 2272 | Seale et al. (2016) | K60576 | Africa | 2013 | 8 |
| 2273 | Seale et al. (2016) | K60575 | Africa | 2013 | 196 |
| 2274 | Seale et al. (2016) | K60574 | Africa | 2013 | 17 |
| 2275 | Seale et al. (2016) | K60573 | Africa | 2013 | 55 |
| 2276 | Seale et al. (2016) | K60572 | Africa | 2013 | 182 |
| 2277 | Seale et al. (2016) | K60566 | Africa | 2013 | 17 |
| 2278 | Seale et al. (2016) | K60565 | Africa | 2013 | 484 |
| 2279 | Seale et al. (2016) | K60564 | Africa | 2013 | 17 |
| 2280 | Seale et al. (2016) | K60516 | Africa | 2013 | 8 |
| 2281 | Seale et al. (2016) | K60508 | Africa | 2013 | 8 |
| 2282 | Seale et al. (2016) | K60442 | Africa | 2013 | 8 |
| 2283 | Seale et al. (2016) | K60434 | Africa | 2013 | 23 |
| 2284 | Seale et al. (2016) | K60433 | Africa | 2013 | 182 |
| 2285 | Seale et al. (2016) | K60432 | Africa | 2013 | 17 |
| 2286 | Seale et al. (2016) | K60428 | Africa | 2013 | 793 |
| 2287 | Seale et al. (2016) | K60427 | Africa | 2013 | 793 |
| 2288 | Seale et al. (2016) | K60426 | Africa | 2013 | 328 |
| 2289 | Seale et al. (2016) | K60291 | Africa | 2013 | 17 |
| 2290 | Seale et al. (2016) | K60290 | Africa | 2013 | 19 |
| 2291 | Seale et al. (2016) | K60028 | Africa | 2012 | 327 |
| 2292 | Seale et al. (2016) | K59962 | Africa | 2012 | 19 |
| 2293 | Seale et al. (2016) | K59927 | Africa | 2012 | 19 |
| 2294 | Seale et al. (2016) | K59917 | Africa | 2012 | 8 |
| 2295 | Seale et al. (2016) | K59881 | Africa | 2012 | 23 |
| 2296 | Seale et al. (2016) | K59813 | Africa | 2012 | 10 |
| 2297 | Seale et al. (2016) | K59793 | Africa | 2012 | 182 |
| 2298 | Seale et al. (2016) | K59792 | Africa | 2012 | 327 |
| 2299 | Seale et al. (2016) | K59791 | Africa | 2012 | 17 |
| 2300 | Seale et al. (2016) | K59790 | Africa | 2012 | 1 |
| 2301 | Seale et al. (2016) | K59789 | Africa | 2012 | 10 |
| 2302 | Seale et al. (2016) | K59778 | Africa | 2012 | 484 |
| 2303 | Seale et al. (2016) | K59777 | Africa | 2012 | 23 |
| 2304 | Seale et al. (2016) | K59776 | Africa | 2012 | 28 |
| 2305 | Seale et al. (2016) | K59775 | Africa | 2012 | 182 |
| 2306 | Seale et al. (2016) | K59774 | Africa | 2012 | 8 |
| 2307 | Seale et al. (2016) | K59773 | Africa | 2012 | 8 |
| 2308 | Seale et al. (2016) | K59772 | Africa | 2012 | 17 |
| 2309 | Seale et al. (2016) | K59754 | Africa | 2012 | 182 |
| 2310 | Seale et al. (2016) | K59753 | Africa | 2012 | 17 |
| 2311 | Seale et al. (2016) | K59752 | Africa | 2012 | 28 |
| 2312 | Seale et al. (2016) | K59749 | Africa | 2012 | 484 |
| 2313 | Seale et al. (2016) | K59748 | Africa | 2012 | 1 |
| 2314 | Seale et al. (2016) | K59747 | Africa | 2012 | 17 |
| 2315 | Seale et al. (2016) | K59746 | Africa | 2012 | 23 |
| 2316 | Seale et al. (2016) | K59733 | Africa | 2012 | 17 |
| 2317 | Seale et al. (2016) | K59731 | Africa | 2012 | 17 |
| 2318 | Seale et al. (2016) | K59730 | Africa | 2012 | 23 |
| 2319 | Seale et al. (2016) | K59729 | Africa | 2012 | 484 |
| 2320 | Seale et al. (2016) | K59712 | Africa | 2012 | 795 |
| 2321 | Seale et al. (2016) | K59711 | Africa | 2012 | 17 |
| 2322 | Seale et al. (2016) | K59710 | Africa | 2012 | 17 |
| 2323 | Seale et al. (2016) | K59673 | Africa | 2012 | 484 |
| 2324 | Seale et al. (2016) | K59648 | Africa | 2012 | 17 |
| 2325 | Seale et al. (2016) | K59646 | Africa | 2012 | 23 |
| 2326 | Seale et al. (2016) | K59645 | Africa | 2012 | 17 |
| 2327 | Seale et al. (2016) | K59605 | Africa | 2012 | 484 |
| 2328 | Seale et al. (2016) | K59604 | Africa | 2012 | 182 |
| 2329 | Seale et al. (2016) | K59594 | Africa | 2012 | 1 |
| 2330 | Seale et al. (2016) | K59593 | Africa | 2012 | 182 |
| 2331 | Seale et al. (2016) | K59591 | Africa | 2012 | 10 |
| 2332 | Seale et al. (2016) | K59590 | Africa | 2012 | 17 |
| 2333 | Seale et al. (2016) | K59589 | Africa | 2012 | 23 |
| 2334 | Seale et al. (2016) | K59580 | Africa | 2012 | 8 |
| 2335 | Seale et al. (2016) | K59567 | Africa | 2012 | 10 |
| 2336 | Seale et al. (2016) | K59566 | Africa | 2012 | 801 |
| 2337 | Seale et al. (2016) | K59564 | Africa | 2012 | 17 |
| 2338 | Seale et al. (2016) | K59528 | Africa | 2012 | 23 |
| 2339 | Seale et al. (2016) | K59527 | Africa | 2012 | 23 |
| 2340 | Seale et al. (2016) | K59526 | Africa | 2012 | 1 |
| 2341 | Seale et al. (2016) | K59524 | Africa | 2012 | 1 |
| 2342 | Seale et al. (2016) | K59522 | Africa | 2012 | 484 |
| 2343 | Seale et al. (2016) | K59518 | Africa | 2012 | 1 |
| 2344 | Seale et al. (2016) | K59501 | Africa | 2012 | 23 |
| 2345 | Seale et al. (2016) | K59500 | Africa | 2012 | 10 |
| 2346 | Seale et al. (2016) | K59499 | Africa | 2012 | 23 |
| 2347 | Seale et al. (2016) | K59498 | Africa | 2012 | 484 |
| 2348 | Seale et al. (2016) | K59495 | Africa | 2012 | 10 |
| 2349 | Seale et al. (2016) | K59494 | Africa | 2012 | 1 |
| 2350 | Seale et al. (2016) | K59463 | Africa | 2012 | 1 |
| 2351 | Seale et al. (2016) | K59441 | Africa | 2012 | 23 |
| 2352 | Seale et al. (2016) | K59440 | Africa | 2012 | 23 |
| 2353 | Seale et al. (2016) | K59439 | Africa | 2012 | 17 |
| 2354 | Seale et al. (2016) | K59426 | Africa | 2012 | 17 |
| 2355 | Seale et al. (2016) | K59425 | Africa | 2012 | 17 |
| 2356 | Seale et al. (2016) | K59423 | Africa | 2012 | 17 |
| 2357 | Seale et al. (2016) | K59411 | Africa | 2012 | 1 |
| 2358 | Seale et al. (2016) | K59410 | Africa | 2012 | 484 |
| 2359 | Seale et al. (2016) | K59354 | Africa | 2012 | 1 |
| 2360 | Seale et al. (2016) | K59352 | Africa | 2012 | 182 |
| 2361 | Seale et al. (2016) | K59292 | Africa | 2012 | 23 |
| 2362 | Seale et al. (2016) | K59290 | Africa | 2012 | 182 |
| 2363 | Seale et al. (2016) | K59288 | Africa | 2012 | 23 |
| 2364 | Seale et al. (2016) | K59245 | Africa | 2012 | 484 |
| 2365 | Seale et al. (2016) | K59230 | Africa | 2012 | 328 |
| 2366 | Seale et al. (2016) | K59219 | Africa | 2012 | 182 |
| 2367 | Seale et al. (2016) | K59218 | Africa | 2012 | 182 |
| 2368 | Seale et al. (2016) | K59217 | Africa | 2012 | 10 |
| 2369 | Seale et al. (2016) | K59200 | Africa | 2012 | 17 |
| 2370 | Seale et al. (2016) | K59199 | Africa | 2012 | 28 |
| 2371 | Seale et al. (2016) | K59184 | Africa | 2012 | 796 |
| 2372 | Seale et al. (2016) | K59183 | Africa | 2012 | 17 |
| 2373 | Seale et al. (2016) | K59182 | Africa | 2012 | 1 |
| 2374 | Seale et al. (2016) | K59181 | Africa | 2012 | 10 |
| 2375 | Seale et al. (2016) | K59167 | Africa | 2012 | 23 |
| 2376 | Seale et al. (2016) | K59154 | Africa | 2012 | 182 |
| 2377 | Seale et al. (2016) | K59148 | Africa | 2012 | 498 |
| 2378 | Seale et al. (2016) | K59126 | Africa | 2012 | 17 |
| 2379 | Seale et al. (2016) | K59113 | Africa | 2012 | 17 |
| 2380 | Seale et al. (2016) | K59097 | Africa | 2012 | 17 |
| 2381 | Seale et al. (2016) | K59095 | Africa | 2012 | 17 |
| 2382 | Seale et al. (2016) | K59094 | Africa | 2012 | 182 |
| 2383 | Seale et al. (2016) | K59086 | Africa | 2012 | 23 |
| 2384 | Seale et al. (2016) | K59071 | Africa | 2012 | 10 |
| 2385 | Seale et al. (2016) | K59070 | Africa | 2012 | 1 |
| 2386 | Seale et al. (2016) | K59068 | Africa | 2012 | 167 |
| 2387 | Seale et al. (2016) | K59067 | Africa | 2012 | 1 |
| 2388 | Seale et al. (2016) | K58954 | Africa | 2012 | 1 |
| 2389 | Seale et al. (2016) | K58915 | Africa | 2012 | 28 |
| 2390 | Seale et al. (2016) | K58906 | Africa | 2012 | 10 |
| 2391 | Seale et al. (2016) | K58905 | Africa | 2012 | 1 |
| 2392 | Seale et al. (2016) | K58884 | Africa | 2012 | 19 |
| 2393 | Seale et al. (2016) | K58864 | Africa | 2012 | 1 |
| 2394 | Seale et al. (2016) | K58833 | Africa | 2012 | 23 |
| 2395 | Seale et al. (2016) | K58820 | Africa | 2012 | 23 |
| 2396 | Seale et al. (2016) | K58806 | Africa | 2012 | 10 |
| 2397 | Seale et al. (2016) | K58805 | Africa | 2012 | 17 |
| 2398 | Seale et al. (2016) | K58801 | Africa | 2012 | 23 |
| 2399 | Seale et al. (2016) | K58795 | Africa | 2012 | 17 |
| 2400 | Seale et al. (2016) | K58773 | Africa | 2012 | 1 |
| 2401 | Seale et al. (2016) | K58771 | Africa | 2012 | 23 |
| 2402 | Seale et al. (2016) | K58760 | Africa | 2012 | 17 |
| 2403 | Seale et al. (2016) | K58758 | Africa | 2012 | 484 |
| 2404 | Seale et al. (2016) | K58737 | Africa | 2012 | 182 |
| 2405 | Seale et al. (2016) | K58736 | Africa | 2012 | 10 |
| 2406 | Seale et al. (2016) | K58734 | Africa | 2012 | 19 |
| 2407 | Seale et al. (2016) | K58711 | Africa | 2012 | 484 |
| 2408 | Seale et al. (2016) | K58710 | Africa | 2012 | 10 |
| 2409 | Seale et al. (2016) | K58694 | Africa | 2012 | 17 |
| 2410 | Seale et al. (2016) | K58690 | Africa | 2012 | 10 |
| 2411 | Seale et al. (2016) | K58634 | Africa | 2012 | 17 |
| 2412 | Seale et al. (2016) | K58631 | Africa | 2012 | 23 |
| 2413 | Seale et al. (2016) | K58613 | Africa | 2012 | 796 |
| 2414 | Seale et al. (2016) | K58611 | Africa | 2012 | 23 |
| 2415 | Seale et al. (2016) | K58610 | Africa | 2012 | 23 |
| 2416 | Seale et al. (2016) | K58593 | Africa | 2012 | 196 |
| 2417 | Seale et al. (2016) | K58592 | Africa | 2012 | 23 |
| 2418 | Seale et al. (2016) | K58591 | Africa | 2012 | 182 |
| 2419 | Seale et al. (2016) | K58574 | Africa | 2012 | 484 |
| 2420 | Seale et al. (2016) | K58551 | Africa | 2012 | 484 |
| 2421 | Seale et al. (2016) | K58495 | Africa | 2012 | 182 |
| 2422 | Seale et al. (2016) | K58493 | Africa | 2012 | 17 |
| 2423 | Seale et al. (2016) | K58491 | Africa | 2012 | 17 |
| 2424 | Seale et al. (2016) | K58489 | Africa | 2012 | 10 |
| 2425 | Seale et al. (2016) | K58460 | Africa | 2012 | 23 |
| 2426 | Seale et al. (2016) | K58435 | Africa | 2012 | 17 |
| 2427 | Seale et al. (2016) | K58418 | Africa | 2012 | 10 |
| 2428 | Seale et al. (2016) | K58417 | Africa | 2012 | 23 |
| 2429 | Seale et al. (2016) | K58331 | Africa | 2012 | 182 |
| 2430 | Seale et al. (2016) | K58317 | Africa | 2012 | 23 |
| 2431 | Seale et al. (2016) | K58316 | Africa | 2012 | 10 |
| 2432 | Seale et al. (2016) | K58308 | Africa | 2012 | 1 |
| 2433 | Seale et al. (2016) | K58307 | Africa | 2012 | 8 |
| 2434 | Seale et al. (2016) | K58306 | Africa | 2012 | 8 |
| 2435 | Seale et al. (2016) | K58274 | Africa | 2012 | 8 |
| 2436 | Seale et al. (2016) | K58243 | Africa | 2012 | 17 |
| 2437 | Seale et al. (2016) | K58242 | Africa | 2012 | 10 |
| 2438 | Seale et al. (2016) | K58211 | Africa | 2012 | 801 |
| 2439 | Seale et al. (2016) | K58185 | Africa | 2012 | 8 |
| 2440 | Seale et al. (2016) | K58137 | Africa | 2012 | 1 |
| 2441 | Seale et al. (2016) | K58136 | Africa | 2012 | 17 |
| 2442 | Seale et al. (2016) | K58105 | Africa | 2012 | 8 |
| 2443 | Seale et al. (2016) | K58104 | Africa | 2012 | 17 |
| 2444 | Seale et al. (2016) | K58057 | Africa | 2012 | 23 |
| 2445 | Seale et al. (2016) | K58056 | Africa | 2012 | 1 |
| 2446 | Seale et al. (2016) | K57992 | Africa | 2012 | 10 |
| 2447 | Seale et al. (2016) | K57976 | Africa | 2012 | 182 |
| 2448 | Seale et al. (2016) | K57973 | Africa | 2012 | 8 |
| 2449 | Seale et al. (2016) | K57972 | Africa | 2012 | 796 |
| 2450 | Seale et al. (2016) | K57969 | Africa | 2012 | 23 |
| 2451 | Seale et al. (2016) | K57965 | Africa | 2012 | 17 |
| 2452 | Seale et al. (2016) | K57964 | Africa | 2012 | 23 |
| 2453 | Seale et al. (2016) | K57912 | Africa | 2012 | 28 |
| 2454 | Seale et al. (2016) | K57910 | Africa | 2012 | 17 |
| 2455 | Seale et al. (2016) | K57878 | Africa | 2012 | 23 |
| 2456 | Seale et al. (2016) | K57877 | Africa | 2012 | 8 |
| 2457 | Seale et al. (2016) | K57843 | Africa | 2012 | 17 |
| 2458 | Seale et al. (2016) | K57809 | Africa | 2012 | 791 |
| 2459 | Seale et al. (2016) | K57808 | Africa | 2012 |  |
| 2460 | Seale et al. (2016) | K57807 | Africa | 2012 | 1 |
| 2461 | Seale et al. (2016) | K57806 | Africa | 2012 | 17 |
| 2462 | Seale et al. (2016) | K57805 | Africa | 2012 | 1 |
| 2463 | Seale et al. (2016) | K57804 | Africa | 2012 | 8 |
| 2464 | Seale et al. (2016) | K57803 | Africa | 2012 | 23 |
| 2465 | Seale et al. (2016) | K57788 | Africa | 2012 | 10 |
| 2466 | Seale et al. (2016) | K57787 | Africa | 2012 | 10 |
| 2467 | Seale et al. (2016) | K57786 | Africa | 2012 | 23 |
| 2468 | Seale et al. (2016) | K57751 | Africa | 2012 | 484 |
| 2469 | Seale et al. (2016) | K57749 | Africa | 2012 | 484 |
| 2470 | Seale et al. (2016) | K57643 | Africa | 2012 | 1 |
| 2471 | Seale et al. (2016) | K57571 | Africa | 2012 | 10 |
| 2472 | Seale et al. (2016) | K57570 | Africa | 2012 | 327 |
| 2473 | Seale et al. (2016) | K57546 | Africa | 2012 | 10 |
| 2474 | Seale et al. (2016) | K57545 | Africa | 2012 | 1 |
| 2475 | Seale et al. (2016) | K57543 | Africa | 2012 | 17 |
| 2476 | Seale et al. (2016) | K57541 | Africa | 2012 | 182 |
| 2477 | Seale et al. (2016) | K57540 | Africa | 2012 | 28 |
| 2478 | Seale et al. (2016) | K57539 | Africa | 2012 | 327 |
| 2479 | Seale et al. (2016) | K57538 | Africa | 2012 | 484 |
| 2480 | Seale et al. (2016) | K57525 | Africa | 2012 | 1 |
| 2481 | Seale et al. (2016) | K57524 | Africa | 2012 | 182 |
| 2482 | Seale et al. (2016) | K57515 | Africa | 2012 | 796 |
| 2483 | Seale et al. (2016) | K57514 | Africa | 2012 | 23 |
| 2484 | Seale et al. (2016) | K57513 | Africa | 2012 | 17 |
| 2485 | Seale et al. (2016) | K57479 | Africa | 2012 | 182 |
| 2486 | Seale et al. (2016) | K57478 | Africa | 2012 | 23 |
| 2487 | Seale et al. (2016) | K57477 | Africa | 2012 | 17 |
| 2488 | Seale et al. (2016) | K57403 | Africa | 2012 | 8 |
| 2489 | Seale et al. (2016) | K57402 | Africa | 2012 | 484 |
| 2490 | Seale et al. (2016) | K57401 | Africa | 2012 | 182 |
| 2491 | Seale et al. (2016) | K57400 | Africa | 2012 | 8 |
| 2492 | Seale et al. (2016) | K57355 | Africa | 2012 | 10 |
| 2493 | Seale et al. (2016) | K57352 | Africa | 2012 | 28 |
| 2494 | Seale et al. (2016) | K57351 | Africa | 2012 | 17 |
| 2495 | Seale et al. (2016) | K57350 | Africa | 2012 | 17 |
| 2496 | Seale et al. (2016) | K57349 | Africa | 2012 | 17 |
| 2497 | Seale et al. (2016) | K57348 | Africa | 2012 | 196 |
| 2498 | Seale et al. (2016) | K57347 | Africa | 2012 | 223 |
| 2499 | Seale et al. (2016) | K57346 | Africa | 2012 | 10 |
| 2500 | Seale et al. (2016) | K57335 | Africa | 2012 | 17 |
| 2501 | Seale et al. (2016) | K57334 | Africa | 2012 | 23 |
| 2502 | Seale et al. (2016) | K57199 | Africa | 2012 | 23 |
| 2503 | Seale et al. (2016) | K57198 | Africa | 2012 | 23 |
| 2504 | Seale et al. (2016) | K57174 | Africa | 2012 | 23 |
| 2505 | Seale et al. (2016) | K57173 | Africa | 2012 | 23 |
| 2506 | Seale et al. (2016) | K57171 | Africa | 2012 | 23 |
| 2507 | Seale et al. (2016) | K57149 | Africa | 2012 | 144 |
| 2508 | Seale et al. (2016) | K57135 | Africa | 2012 | 23 |
| 2509 | Seale et al. (2016) | K57124 | Africa | 2012 | 196 |
| 2510 | Seale et al. (2016) | K57093 | Africa | 2012 | 23 |
| 2511 | Seale et al. (2016) | K57092 | Africa | 2012 | 23 |
| 2512 | Seale et al. (2016) | K57090 | Africa | 2012 | 23 |
| 2513 | Seale et al. (2016) | K57089 | Africa | 2012 | 17 |
| 2514 | Seale et al. (2016) | K57069 | Africa | 2012 | 327 |
| 2515 | Seale et al. (2016) | K57068 | Africa | 2012 | 182 |
| 2516 | Seale et al. (2016) | K57066 | Africa | 2012 | 28 |
| 2517 | Seale et al. (2016) | K57064 | Africa | 2012 | 23 |
| 2518 | Seale et al. (2016) | K57062 | Africa | 2012 | 10 |
| 2519 | Seale et al. (2016) | K57056 | Africa | 2012 | 10 |
| 2520 | Seale et al. (2016) | K57050 | Africa | 2012 | 196 |
| 2521 | Seale et al. (2016) | K57049 | Africa | 2012 | 17 |
| 2522 | Seale et al. (2016) | K57015 | Africa | 2012 | 23 |
| 2523 | Seale et al. (2016) | K56996 | Africa | 2012 | 1 |
| 2524 | Seale et al. (2016) | K56995 | Africa | 2012 | 484 |
| 2525 | Seale et al. (2016) | K56992 | Africa | 2012 | 8 |
| 2526 | Seale et al. (2016) | K56991 | Africa | 2012 | 484 |
| 2527 | Seale et al. (2016) | K56989 | Africa | 2012 | 484 |
| 2528 | Seale et al. (2016) | K56988 | Africa | 2012 | 17 |
| 2529 | Seale et al. (2016) | K56981 | Africa | 2012 | 790 |
| 2530 | Seale et al. (2016) | K56957 | Africa | 2012 | 23 |
| 2531 | Seale et al. (2016) | K56949 | Africa | 2012 | 17 |
| 2532 | Seale et al. (2016) | K56943 | Africa | 2012 | 327 |
| 2533 | Seale et al. (2016) | K56917 | Africa | 2012 | 486 |
| 2534 | Seale et al. (2016) | K56914 | Africa | 2012 | 23 |
| 2535 | Seale et al. (2016) | K56913 | Africa | 2012 | 23 |
| 2536 | Seale et al. (2016) | K56889 | Africa | 2012 | 17 |
| 2537 | Seale et al. (2016) | K56872 | Africa | 2012 | 8 |
| 2538 | Seale et al. (2016) | K56866 | Africa | 2012 | 484 |
| 2539 | Seale et al. (2016) | K56857 | Africa | 2012 | 17 |
| 2540 | Seale et al. (2016) | K56814 | Africa | 2012 | 484 |
| 2541 | Seale et al. (2016) | K56813 | Africa | 2012 | 327 |
| 2542 | Seale et al. (2016) | K56810 | Africa | 2012 | 23 |
| 2543 | Seale et al. (2016) | K56809 | Africa | 2012 | 17 |
| 2544 | Seale et al. (2016) | K56802 | Africa | 2012 | 8 |
| 2545 | Seale et al. (2016) | K56800 | Africa | 2012 | 23 |
| 2546 | Seale et al. (2016) | K56799 | Africa | 2012 | 19 |
| 2547 | Seale et al. (2016) | K56798 | Africa | 2012 | 23 |
| 2548 | Seale et al. (2016) | K56797 | Africa | 2012 | 182 |
| 2549 | Seale et al. (2016) | K56796 | Africa | 2012 | 17 |
| 2550 | Seale et al. (2016) | K56794 | Africa | 2012 | 17 |
| 2551 | Seale et al. (2016) | K56781 | Africa | 2012 | 23 |
| 2552 | Seale et al. (2016) | K56778 | Africa | 2012 | 23 |
| 2553 | Seale et al. (2016) | K56777 | Africa | 2012 | 23 |
| 2554 | Seale et al. (2016) | K56776 | Africa | 2012 | 23 |
| 2555 | Seale et al. (2016) | K56758 | Africa | 2012 | 23 |
| 2556 | Seale et al. (2016) | K56745 | Africa | 2012 | 23 |
| 2557 | Seale et al. (2016) | K56722 | Africa | 2012 | 182 |
| 2558 | Seale et al. (2016) | K56721 | Africa | 2012 | 484 |
| 2559 | Seale et al. (2016) | K56719 | Africa | 2012 | 8 |
| 2560 | Seale et al. (2016) | K56718 | Africa | 2012 | 23 |
| 2561 | Seale et al. (2016) | K56704 | Africa | 2012 | 182 |
| 2562 | Seale et al. (2016) | K56702 | Africa | 2012 | 23 |
| 2563 | Seale et al. (2016) | K56689 | Africa | 2012 | 23 |
| 2564 | Seale et al. (2016) | K56687 | Africa | 2012 | 23 |
| 2565 | Seale et al. (2016) | K56685 | Africa | 2012 | 8 |
| 2566 | Seale et al. (2016) | K56684 | Africa | 2012 | 10 |
| 2567 | Seale et al. (2016) | K56668 | Africa | 2012 | 10 |
| 2568 | Seale et al. (2016) | K56667 | Africa | 2012 | 1 |
| 2569 | Seale et al. (2016) | K56665 | Africa | 2012 | 484 |
| 2570 | Seale et al. (2016) | K56664 | Africa | 2012 | 484 |
| 2571 | Seale et al. (2016) | K56646 | Africa | 2012 | 3 |
| 2572 | Seale et al. (2016) | K56645 | Africa | 2012 | 17 |
| 2573 | Seale et al. (2016) | K56635 | Africa | 2012 | 327 |
| 2574 | Seale et al. (2016) | K56634 | Africa | 2012 | 23 |
| 2575 | Seale et al. (2016) | K56619 | Africa | 2012 | 23 |
| 2576 | Seale et al. (2016) | K56615 | Africa | 2012 | 17 |
| 2577 | Seale et al. (2016) | K56614 | Africa | 2012 | 182 |
| 2578 | Seale et al. (2016) | K56613 | Africa | 2012 | 28 |
| 2579 | Seale et al. (2016) | K56612 | Africa | 2012 | 498 |
| 2580 | Seale et al. (2016) | K56611 | Africa | 2012 | 486 |
| 2581 | Seale et al. (2016) | K56610 | Africa | 2012 | 23 |
| 2582 | Seale et al. (2016) | K56586 | Africa | 2012 | 10 |
| 2583 | Seale et al. (2016) | K56585 | Africa | 2012 | 10 |
| 2584 | Seale et al. (2016) | K56583 | Africa | 2012 | 24 |
| 2585 | Seale et al. (2016) | K56581 | Africa | 2012 | 24 |
| 2586 | Seale et al. (2016) | K56570 | Africa | 2012 | 23 |
| 2587 | Seale et al. (2016) | K56555 | Africa | 2012 | 23 |
| 2588 | Seale et al. (2016) | K56545 | Africa | 2012 | 28 |
| 2589 | Seale et al. (2016) | K56509 | Africa | 2012 | 28 |
| 2590 | Seale et al. (2016) | K56508 | Africa | 2012 | 17 |
| 2591 | Seale et al. (2016) | K56507 | Africa | 2012 | 23 |
| 2592 | Seale et al. (2016) | K56506 | Africa | 2012 | 19 |
| 2593 | Seale et al. (2016) | K56494 | Africa | 2012 | 23 |
| 2594 | Seale et al. (2016) | K56493 | Africa | 2012 | 10 |
| 2595 | Seale et al. (2016) | K56492 | Africa | 2012 | 1 |
| 2596 | Seale et al. (2016) | K56487 | Africa | 2012 | 1 |
| 2597 | Seale et al. (2016) | K56486 | Africa | 2012 | 23 |
| 2598 | Seale et al. (2016) | K56484 | Africa | 2012 | 498 |
| 2599 | Seale et al. (2016) | K56483 | Africa | 2012 | 10 |
| 2600 | Seale et al. (2016) | K56482 | Africa | 2012 | 182 |
| 2601 | Seale et al. (2016) | K56479 | Africa | 2012 | 8 |
| 2602 | Seale et al. (2016) | K56477 | Africa | 2012 | 23 |
| 2603 | Seale et al. (2016) | K56463 | Africa | 2012 | 182 |
| 2604 | Seale et al. (2016) | K56453 | Africa | 2012 | 196 |
| 2605 | Seale et al. (2016) | K56442 | Africa | 2012 | 23 |
| 2606 | Seale et al. (2016) | K56436 | Africa | 2012 | 23 |
| 2607 | Seale et al. (2016) | K56435 | Africa | 2012 | 23 |
| 2608 | Seale et al. (2016) | K56432 | Africa | 2012 | 23 |
| 2609 | Seale et al. (2016) | K56431 | Africa | 2012 | 23 |
| 2610 | Seale et al. (2016) | K56419 | Africa | 2012 | 23 |
| 2611 | Seale et al. (2016) | K56418 | Africa | 2012 | 23 |
| 2612 | Seale et al. (2016) | K56377 | Africa | 2012 | 23 |
| 2613 | Seale et al. (2016) | K56374 | Africa | 2012 | 196 |
| 2614 | Seale et al. (2016) | K56373 | Africa | 2012 | 182 |
| 2615 | Seale et al. (2016) | K56372 | Africa | 2012 | 792 |
| 2616 | Seale et al. (2016) | K56371 | Africa | 2012 | 17 |
| 2617 | Seale et al. (2016) | K56370 | Africa | 2012 | 1 |
| 2618 | Seale et al. (2016) | K56369 | Africa | 2012 | 17 |
| 2619 | Seale et al. (2016) | K56367 | Africa | 2012 | 327 |
| 2620 | Seale et al. (2016) | K56366 | Africa | 2012 | 1 |
| 2621 | Seale et al. (2016) | K56341 | Africa | 2012 | 8 |
| 2622 | Seale et al. (2016) | K56333 | Africa | 2012 | 327 |
| 2623 | Seale et al. (2016) | K56278 | Africa | 2012 | 8 |
| 2624 | Seale et al. (2016) | K56264 | Africa | 2012 | 19 |
| 2625 | Seale et al. (2016) | K56263 | Africa | 2012 | 1 |
| 2626 | Seale et al. (2016) | K56261 | Africa | 2012 | 19 |
| 2627 | Seale et al. (2016) | K56247 | Africa | 2012 | 23 |
| 2628 | Seale et al. (2016) | K56246 | Africa | 2012 | 17 |
| 2629 | Seale et al. (2016) | K56245 | Africa | 2012 | 17 |
| 2630 | Seale et al. (2016) | K56244 | Africa | 2012 | 1 |
| 2631 | Seale et al. (2016) | K56235 | Africa | 2012 | 788 |
| 2632 | Seale et al. (2016) | K56234 | Africa | 2012 | 23 |
| 2633 | Seale et al. (2016) | K56233 | Africa | 2012 | 23 |
| 2634 | Seale et al. (2016) | K56232 | Africa | 2012 | 23 |
| 2635 | Seale et al. (2016) | K56231 | Africa | 2012 | 17 |
| 2636 | Seale et al. (2016) | K56228 | Africa | 2012 | 17 |
| 2637 | Seale et al. (2016) | K56218 | Africa | 2012 | 17 |
| 2638 | Seale et al. (2016) | K56217 | Africa | 2012 | 23 |
| 2639 | Seale et al. (2016) | K56198 | Africa | 2012 | 23 |
| 2640 | Seale et al. (2016) | K56197 | Africa | 2012 | 19 |
| 2641 | Seale et al. (2016) | K56196 | Africa | 2012 | 10 |
| 2642 | Seale et al. (2016) | K56195 | Africa | 2012 | 801 |
| 2643 | Seale et al. (2016) | K56194 | Africa | 2012 | 182 |
| 2644 | Seale et al. (2016) | K56193 | Africa | 2012 | 28 |
| 2645 | Seale et al. (2016) | K56186 | Africa | 2012 | 498 |
| 2646 | Seale et al. (2016) | K56185 | Africa | 2012 | 17 |
| 2647 | Seale et al. (2016) | K56184 | Africa | 2012 | 182 |
| 2648 | Seale et al. (2016) | K56183 | Africa | 2012 | 17 |
| 2649 | Seale et al. (2016) | K56159 | Africa | 2012 | 1 |
| 2650 | Seale et al. (2016) | K56114 | Africa | 2012 | 1 |
| 2651 | Seale et al. (2016) | K56113 | Africa | 2012 | 23 |
| 2652 | Seale et al. (2016) | K56076 | Africa | 2012 | 10 |
| 2653 | Seale et al. (2016) | K56074 | Africa | 2012 | 182 |
| 2654 | Seale et al. (2016) | K56073 | Africa | 2012 | 182 |
| 2655 | Seale et al. (2016) | K56071 | Africa | 2012 | 484 |
| 2656 | Seale et al. (2016) | K56070 | Africa | 2012 | 10 |
| 2657 | Seale et al. (2016) | K56069 | Africa | 2012 | 23 |
| 2658 | Seale et al. (2016) | K56064 | Africa | 2012 | 484 |
| 2659 | Seale et al. (2016) | K56062 | Africa | 2012 | 17 |
| 2660 | Seale et al. (2016) | K56061 | Africa | 2012 | 484 |
| 2661 | Seale et al. (2016) | K56046 | Africa | 2012 | 182 |
| 2662 | Seale et al. (2016) | K56031 | Africa | 2012 | 484 |
| 2663 | Seale et al. (2016) | K55997 | Africa | 2012 | 23 |
| 2664 | Seale et al. (2016) | K55996 | Africa | 2012 | 8 |
| 2665 | Seale et al. (2016) | K55980 | Africa | 2012 | 23 |
| 2666 | Seale et al. (2016) | K55979 | Africa | 2012 | 23 |
| 2667 | Seale et al. (2016) | K55960 | Africa | 2012 | 327 |
| 2668 | Seale et al. (2016) | K55959 | Africa | 2012 | 182 |
| 2669 | Seale et al. (2016) | K55958 | Africa | 2012 | 28 |
| 2670 | Seale et al. (2016) | K55896 | Africa | 2012 | 23 |
| 2671 | Seale et al. (2016) | K55895 | Africa | 2012 | 182 |
| 2672 | Seale et al. (2016) | K55881 | Africa | 2012 | 484 |
| 2673 | Seale et al. (2016) | K55880 | Africa | 2012 | 23 |
| 2674 | Seale et al. (2016) | K55761 | Africa | 2012 | 23 |
| 2675 | Seale et al. (2016) | K55711 | Africa | 2012 | 796 |
| 2676 | Seale et al. (2016) | K55655 | Africa | 2012 | 801 |
| 2677 | Seale et al. (2016) | K55631 | Africa | 2012 | 17 |
| 2678 | Seale et al. (2016) | K55630 | Africa | 2012 | 10 |
| 2679 | Seale et al. (2016) | K55603 | Africa | 2012 | 17 |
| 2680 | Seale et al. (2016) | K55594 | Africa | 2012 | 484 |
| 2681 | Seale et al. (2016) | K55593 | Africa | 2012 | 17 |
| 2682 | Seale et al. (2016) | K55547 | Africa | 2012 | 17 |
| 2683 | Seale et al. (2016) | K55532 | Africa | 2012 | 23 |
| 2684 | Seale et al. (2016) | K55531 | Africa | 2012 | 10 |
| 2685 | Seale et al. (2016) | K55530 | Africa | 2012 | 328 |
| 2686 | Seale et al. (2016) | K55529 | Africa | 2012 | 10 |
| 2687 | Seale et al. (2016) | K55513 | Africa | 2012 | 328 |
| 2688 | Seale et al. (2016) | K55494 | Africa | 2012 | 785 |
| 2689 | Seale et al. (2016) | K55493 | Africa | 2012 | 17 |
| 2690 | Seale et al. (2016) | K55454 | Africa | 2012 | 8 |
| 2691 | Seale et al. (2016) | K55453 | Africa | 2012 | 1 |
| 2692 | Seale et al. (2016) | K55452 | Africa | 2012 | 24 |
| 2693 | Seale et al. (2016) | K55442 | Africa | 2012 | 23 |
| 2694 | Seale et al. (2016) | K55441 | Africa | 2012 | 23 |
| 2695 | Seale et al. (2016) | K55427 | Africa | 2012 | 328 |
| 2696 | Seale et al. (2016) | K55425 | Africa | 2012 | 10 |
| 2697 | Seale et al. (2016) | K55416 | Africa | 2012 | 17 |
| 2698 | Seale et al. (2016) | K55398 | Africa | 2012 | 327 |
| 2699 | Seale et al. (2016) | K55357 | Africa | 2012 | 23 |
| 2700 | Seale et al. (2016) | K55356 | Africa | 2012 | 327 |
| 2701 | Seale et al. (2016) | K55320 | Africa | 2012 | 17 |
| 2702 | Seale et al. (2016) | K55292 | Africa | 2012 | 17 |
| 2703 | Seale et al. (2016) | K55286 | Africa | 2012 | 291 |
| 2704 | Seale et al. (2016) | K55280 | Africa | 2012 | 23 |
| 2705 | Seale et al. (2016) | K55234 | Africa | 2012 | 10 |
| 2706 | Seale et al. (2016) | K55219 | Africa | 2012 | 196 |
| 2707 | Seale et al. (2016) | K55218 | Africa | 2012 | 8 |
| 2708 | Seale et al. (2016) | K52864 | Africa | 2012 | 23 |
| 2709 | Seale et al. (2016) | K52862 | Africa | 2012 | 17 |
| 2710 | Seale et al. (2016) | K52817 | Africa | 2012 | 327 |
| 2711 | Seale et al. (2016) | K52808 | Africa | 2012 | 28 |
| 2712 | Seale et al. (2016) | K52792 | Africa | 2012 | 10 |
| 2713 | Seale et al. (2016) | K52781 | Africa | 2012 | 1 |
| 2714 | Seale et al. (2016) | K52780 | Africa | 2012 | 182 |
| 2715 | Seale et al. (2016) | K52779 | Africa | 2012 | 327 |
| 2716 | Seale et al. (2016) | K52774 | Africa | 2012 | 23 |
| 2717 | Seale et al. (2016) | K52773 | Africa | 2012 | 17 |
| 2718 | Seale et al. (2016) | K52692 | Africa | 2012 | 23 |
| 2719 | Seale et al. (2016) | K52674 | Africa | 2012 | 10 |
| 2720 | Seale et al. (2016) | K52662 | Africa | 2012 | 10 |
| 2721 | Seale et al. (2016) | K52647 | Africa | 2012 | 17 |
| 2722 | Seale et al. (2016) | K52646 | Africa | 2012 | 17 |
| 2723 | Seale et al. (2016) | K52623 | Africa | 2012 | 17 |
| 2724 | Seale et al. (2016) | K52615 | Africa | 2012 | 182 |
| 2725 | Seale et al. (2016) | K52539 | Africa | 2012 | 23 |
| 2726 | Seale et al. (2016) | K52487 | Africa | 2012 | 1 |
| 2727 | Seale et al. (2016) | K52486 | Africa | 2012 | 17 |
| 2728 | Seale et al. (2016) | K52459 | Africa | 2012 | 10 |
| 2729 | Seale et al. (2016) | K52458 | Africa | 2012 | 196 |
| 2730 | Seale et al. (2016) | K52454 | Africa | 2012 | 327 |
| 2731 | Seale et al. (2016) | K52452 | Africa | 2012 | 24 |
| 2732 | Seale et al. (2016) | K52433 | Africa | 2012 | 1 |
| 2733 | Seale et al. (2016) | K52432 | Africa | 2012 | 10 |
| 2734 | Seale et al. (2016) | K52396 | Africa | 2012 | 182 |
| 2735 | Seale et al. (2016) | K52368 | Africa | 2012 | 1 |
| 2736 | Seale et al. (2016) | K52367 | Africa | 2012 | 17 |
| 2737 | Seale et al. (2016) | K52346 | Africa | 2012 | 17 |
| 2738 | Seale et al. (2016) | K52345 | Africa | 2012 | 19 |
| 2739 | Seale et al. (2016) | K52318 | Africa | 2012 | 1 |
| 2740 | Seale et al. (2016) | K52315 | Africa | 2012 | 17 |
| 2741 | Seale et al. (2016) | K52314 | Africa | 2012 | 8 |
| 2742 | Seale et al. (2016) | K52274 | Africa | 2012 | 1 |
| 2743 | Seale et al. (2016) | K52157 | Africa | 2012 | 328 |
| 2744 | Seale et al. (2016) | K52086 | Africa | 2012 | 787 |
| 2745 | Seale et al. (2016) | K52085 | Africa | 2012 | 182 |
| 2746 | Seale et al. (2016) | K52084 | Africa | 2012 | 23 |
| 2747 | Seale et al. (2016) | K52083 | Africa | 2012 | 1 |
| 2748 | Seale et al. (2016) | K52082 | Africa | 2012 | 28 |
| 2749 | Seale et al. (2016) | K52067 | Africa | 2012 | 28 |
| 2750 | Seale et al. (2016) | K52065 | Africa | 2012 | 801 |
| 2751 | Seale et al. (2016) | K52064 | Africa | 2012 | 10 |
| 2752 | Seale et al. (2016) | K52050 | Africa | 2012 | 1 |
| 2753 | Seale et al. (2016) | K52049 | Africa | 2012 | 17 |
| 2754 | Seale et al. (2016) | K52048 | Africa | 2012 | 28 |
| 2755 | Seale et al. (2016) | K51965 | Africa | 2012 | 327 |
| 2756 | Seale et al. (2016) | K51887 | Africa | 2011 | 327 |
| 2757 | Seale et al. (2016) | K51855 | Africa | 2011 | 28 |
| 2758 | Seale et al. (2016) | K51818 | Africa | 2011 | 17 |
| 2759 | Seale et al. (2016) | K51792 | Africa | 2011 | 10 |
| 2760 | Seale et al. (2016) | K51745 | Africa | 2011 | 17 |
| 2761 | Seale et al. (2016) | K51742 | Africa | 2011 | 17 |
| 2762 | Seale et al. (2016) | K51738 | Africa | 2011 | 17 |
| 2763 | Seale et al. (2016) | K51705 | Africa | 2011 | 19 |
| 2764 | Seale et al. (2016) | K51704 | Africa | 2011 |  |
| 2765 | Seale et al. (2016) | K51698 | Africa | 2011 | 1 |
| 2766 | Seale et al. (2016) | K51617 | Africa | 2011 | 17 |
| 2767 | Seale et al. (2016) | K51608 | Africa | 2011 | 17 |
| 2768 | Seale et al. (2016) | K51590 | Africa | 2011 | 484 |
| 2769 | Seale et al. (2016) | K51578 | Africa | 2011 | 17 |
| 2770 | Seale et al. (2016) | K51572 | Africa | 2011 | 484 |
| 2771 | Seale et al. (2016) | K51527 | Africa | 2011 | 182 |
| 2772 | Seale et al. (2016) | K51526 | Africa | 2011 | 1 |
| 2773 | Seale et al. (2016) | K51525 | Africa | 2011 | 28 |
| 2774 | Seale et al. (2016) | K51478 | Africa | 2011 | 23 |
| 2775 | Seale et al. (2016) | K51463 | Africa | 2011 | 8 |
| 2776 | Seale et al. (2016) | K51420 | Africa | 2011 | 796 |
| 2777 | Seale et al. (2016) | K51361 | Africa | 2011 | 10 |
| 2778 | Seale et al. (2016) | K51349 | Africa | 2011 | 196 |
| 2779 | Seale et al. (2016) | K51348 | Africa | 2011 | 484 |
| 2780 | Seale et al. (2016) | K51329 | Africa | 2011 | 23 |
| 2781 | Seale et al. (2016) | K51319 | Africa | 2011 | 196 |
| 2782 | Seale et al. (2016) | K51318 | Africa | 2011 | 2 |
| 2783 | Seale et al. (2016) | K51280 | Africa | 2011 | 28 |
| 2784 | Seale et al. (2016) | K51261 | Africa | 2011 | 8 |
| 2785 | Seale et al. (2016) | K51260 | Africa | 2011 | 17 |
| 2786 | Seale et al. (2016) | K51258 | Africa | 2011 | 1 |
| 2787 | Seale et al. (2016) | K51257 | Africa | 2011 | 23 |
| 2788 | Seale et al. (2016) | K51256 | Africa | 2011 | 182 |
| 2789 | Seale et al. (2016) | K51255 | Africa | 2011 | 802 |
| 2790 | Seale et al. (2016) | K51230 | Africa | 2011 | 10 |
| 2791 | Seale et al. (2016) | K51216 | Africa | 2011 | 28 |
| 2792 | Seale et al. (2016) | K51201 | Africa | 2011 | 17 |
| 2793 | Seale et al. (2016) | K51175 | Africa | 2011 | 23 |
| 2794 | Seale et al. (2016) | K51157 | Africa | 2011 | 182 |
| 2795 | Seale et al. (2016) | K51139 | Africa | 2011 | 8 |
| 2796 | Seale et al. (2016) | K51133 | Africa | 2011 | 327 |
| 2797 | Seale et al. (2016) | K51118 | Africa | 2011 | 182 |
| 2798 | Seale et al. (2016) | K51091 | Africa | 2011 | 17 |
| 2799 | Seale et al. (2016) | K50972 | Africa | 2011 | 17 |
| 2800 | Seale et al. (2016) | K50939 | Africa | 2011 | 182 |
| 2801 | Seale et al. (2016) | K50938 | Africa | 2011 | 23 |
| 2802 | Seale et al. (2016) | K50909 | Africa | 2011 | 10 |
| 2803 | Seale et al. (2016) | K50877 | Africa | 2011 | 784 |
| 2804 | Seale et al. (2016) | K50864 | Africa | 2011 | 10 |
| 2805 | Seale et al. (2016) | K50827 | Africa | 2011 | 28 |
| 2806 | Seale et al. (2016) | K50826 | Africa | 2011 | 28 |
| 2807 | Seale et al. (2016) | K50792 | Africa | 2011 | 17 |
| 2808 | Seale et al. (2016) | K50791 | Africa | 2011 | 17 |
| 2809 | Seale et al. (2016) | K50790 | Africa | 2011 | 23 |
| 2810 | Seale et al. (2016) | K50725 | Africa | 2011 | 23 |
| 2811 | Seale et al. (2016) | K50724 | Africa | 2011 | 17 |
| 2812 | Seale et al. (2016) | K50709 | Africa | 2011 | 484 |
| 2813 | Seale et al. (2016) | K50708 | Africa | 2011 | 23 |
| 2814 | Seale et al. (2016) | K50669 | Africa | 2011 | 196 |
| 2815 | Seale et al. (2016) | K50525 | Africa | 2011 | 8 |
| 2816 | Seale et al. (2016) | K50492 | Africa | 2011 | 23 |
| 2817 | Seale et al. (2016) | K50466 | Africa | 2011 | 788 |
| 2818 | Seale et al. (2016) | K50442 | Africa | 2011 | 8 |
| 2819 | Seale et al. (2016) | K50441 | Africa | 2011 | 17 |
| 2820 | Seale et al. (2016) | K50426 | Africa | 2011 | 182 |
| 2821 | Seale et al. (2016) | K50415 | Africa | 2011 | 1 |
| 2822 | Seale et al. (2016) | K50414 | Africa | 2011 | 17 |
| 2823 | Seale et al. (2016) | K50387 | Africa | 2011 | 10 |
| 2824 | Seale et al. (2016) | K50365 | Africa | 2011 | 8 |
| 2825 | Seale et al. (2016) | K50364 | Africa | 2011 | 327 |
| 2826 | Seale et al. (2016) | K50342 | Africa | 2011 | 23 |
| 2827 | Seale et al. (2016) | K50227 | Africa | 2011 | 796 |
| 2828 | Seale et al. (2016) | K50223 | Africa | 2011 | 17 |
| 2829 | Seale et al. (2016) | K50222 | Africa | 2011 | 182 |
| 2830 | Seale et al. (2016) | K50167 | Africa | 2011 | 182 |
| 2831 | Seale et al. (2016) | K50146 | Africa | 2011 | 484 |
| 2832 | Seale et al. (2016) | K50145 | Africa | 2011 | 23 |
| 2833 | Seale et al. (2016) | K50144 | Africa | 2011 | 8 |
| 2834 | Seale et al. (2016) | K50143 | Africa | 2011 | 182 |
| 2835 | Seale et al. (2016) | K50142 | Africa | 2011 | 484 |
| 2836 | Seale et al. (2016) | K50140 | Africa | 2011 | 17 |
| 2837 | Seale et al. (2016) | K50057 | Africa | 2011 | 484 |
| 2838 | Seale et al. (2016) | K50009 | Africa | 2011 | 327 |
| 2839 | Seale et al. (2016) | K50008 | Africa | 2011 | 28 |
| 2840 | Seale et al. (2016) | K50007 | Africa | 2011 | 19 |
| 2841 | Seale et al. (2016) | K50006 | Africa | 2011 | 28 |
| 2842 | Seale et al. (2016) | K49986 | Africa | 2011 | 19 |
| 2843 | Seale et al. (2016) | K49981 | Africa | 2011 | 17 |
| 2844 | Seale et al. (2016) | K49970 | Africa | 2011 | 17 |
| 2845 | Seale et al. (2016) | K49969 | Africa | 2011 | 1 |
| 2846 | Seale et al. (2016) | K49965 | Africa | 2011 | 17 |
| 2847 | Seale et al. (2016) | K49933 | Africa | 2011 | 484 |
| 2848 | Seale et al. (2016) | K49931 | Africa | 2011 | 23 |
| 2849 | Seale et al. (2016) | K49917 | Africa | 2011 | 23 |
| 2850 | Seale et al. (2016) | K49870 | Africa | 2011 | 10 |
| 2851 | Seale et al. (2016) | K49839 | Africa | 2011 | 19 |
| 2852 | Seale et al. (2016) | K49831 | Africa | 2011 | 484 |
| 2853 | Seale et al. (2016) | K49793 | Africa | 2011 | 23 |
| 2854 | Seale et al. (2016) | K49791 | Africa | 2011 | 23 |
| 2855 | Seale et al. (2016) | K49768 | Africa | 2011 | 28 |
| 2856 | Seale et al. (2016) | K49740 | Africa | 2011 | 17 |
| 2857 | Seale et al. (2016) | K49739 | Africa | 2011 | 8 |
| 2858 | Seale et al. (2016) | K49738 | Africa | 2011 | 484 |
| 2859 | Seale et al. (2016) | K23965 | Africa | 2007 | 17 |
| 2860 | Seale et al. (2016) | K23286 | Africa | 2007 | 23 |
| 2861 | Seale et al. (2016) | K20349 | Africa | 2006 | 17 |
| 2862 | Seale et al. (2016) | K20332 | Africa | 2006 | 17 |
| 2863 | Seale et al. (2016) | K20251 | Africa |  | 804 |
| 2864 | Seale et al. (2016) | K20230 | Africa | 2006 | 8 |
| 2865 | Seale et al. (2016) | K20019 | Africa | 2006 | 17 |
| 2866 | Seale et al. (2016) | K19924 | Africa | 2006 | 484 |
| 2867 | Seale et al. (2016) | K19244 | Africa | 2006 | 17 |
| 2868 | Seale et al. (2016) | K19212 | Africa | 2006 | 17 |
| 2869 | Seale et al. (2016) | K19170 | Africa | 2006 | 803 |
| 2870 | Seale et al. (2016) | K18723 | Africa | 2006 | 484 |
| 2871 | Seale et al. (2016) | K17900 | Africa | 2006 | 17 |
| 2872 | Seale et al. (2016) | K17897 | Africa | 2006 | 147 |
| 2873 | Seale et al. (2016) | K17389 | Africa | 2005 | 17 |
| 2874 | Seale et al. (2016) | K17308 | Africa | 2005 | 17 |
| 2875 | Seale et al. (2016) | K17224 | Africa | 2005 | 196 |
| 2876 | Seale et al. (2016) | K17110 | Africa | 2005 | 17 |
| 2877 | Seale et al. (2016) | K16827 | Africa | 2005 | 10 |
| 2878 | Seale et al. (2016) | K16755 | Africa | 2005 | 17 |
| 2879 | Seale et al. (2016) | K16534 | Africa | 2005 | 17 |
| 2880 | Seale et al. (2016) | K14206 | Africa | 2005 | 17 |
| 2881 | Seale et al. (2016) | K13758 | Africa | 2004 | 17 |
| 2882 | Seale et al. (2016) | K11385 | Africa | 2003 | 17 |
| 2883 | Seale et al. (2016) | K59563 | Africa | 2012 | 17 |
| 2884 | Seale et al. (2016) | K57810 | Africa | 2012 | 17 |
| 2885 | Seale et al. (2016) | K57085 | Africa | 2012 | 17 |
| 2886 | Seale et al. (2016) | K57018 | Africa | 2012 | 2 |
| 2887 | Seale et al. (2016) | K10268 | Africa | 2003 | 17 |
| 2888 | Seale et al. (2016) | K56640 | Africa | 2012 | 17 |
| 2889 | Seale et al. (2016) | K55826 | Africa | 2012 | 17 |
| 2890 | Seale et al. (2016) | K55813 | Africa | 2012 | 17 |
| 2891 | Seale et al. (2016) | K9182 | Africa | 2002 | 17 |
| 2892 | Seale et al. (2016) | K52758 | Africa | 2012 | 23 |
| 2893 | Seale et al. (2016) | K8568 | Africa | 2002 | 23 |
| 2894 | Seale et al. (2016) | K8555 | Africa | 2002 | 17 |
| 2895 | Seale et al. (2016) | K8309 | Africa | 2002 | 17 |
| 2896 | Seale et al. (2016) | K50468 | Africa |  | 792 |
| 2897 | Seale et al. (2016) | K7834 | Africa | 2001 | 10 |
| 2898 | Seale et al. (2016) | K48612 | Africa | 2011 | 484 |
| 2899 | Seale et al. (2016) | K7453 | Africa | 2001 | 19 |
| 2900 | Seale et al. (2016) | K48051 | Africa | 2011 | 17 |
| 2901 | Seale et al. (2016) | K7229 | Africa | 2001 | 17 |
| 2902 | Seale et al. (2016) | K47340 | Africa | 2011 | 484 |
| 2903 | Seale et al. (2016) | K7129 | Africa | 2001 | 23 |
| 2904 | Seale et al. (2016) | K6781 | Africa | 2001 | 23 |
| 2905 | Seale et al. (2016) | K45678 | Africa | 2010 | 17 |
| 2906 | Seale et al. (2016) | K6666 | Africa | 2000 | 484 |
| 2907 | Seale et al. (2016) | K6625 | Africa | 2000 | 17 |
| 2908 | Seale et al. (2016) | K6245 | Africa | 2000 | 17 |
| 2909 | Seale et al. (2016) | K6234 | Africa | 2000 | 17 |
| 2910 | Seale et al. (2016) | K6229 | Africa | 2000 | 17 |
| 2911 | Seale et al. (2016) | K6209 | Africa | 2000 | 23 |
| 2912 | Seale et al. (2016) | K5666 | Africa | 2000 | 23 |
| 2913 | Seale et al. (2016) | K5136 | Africa | 1999 | 17 |
| 2914 | Seale et al. (2016) | K4958 | Africa | 1999 | 17 |
| 2915 | Seale et al. (2016) | K39258 | Africa | 2009 | 484 |
| 2916 | Seale et al. (2016) | K38783 | Africa | 2009 | 17 |
| 2917 | Seale et al. (2016) | K36829 | Africa | 2009 | 484 |
| 2918 | Seale et al. (2016) | K4436 | Africa | 1999 | 23 |
| 2919 | Seale et al. (2016) | K4340 | Africa | 1999 | 17 |
| 2920 | Seale et al. (2016) | K36235 | Africa | 2009 | 182 |
| 2921 | Seale et al. (2016) | K36205 | Africa | 2009 | 182 |
| 2922 | Seale et al. (2016) | K35752 | Africa | 2008 | 8 |
| 2923 | Seale et al. (2016) | K3931 | Africa | 1998 | 23 |
| 2924 | Seale et al. (2016) | K33921 | Africa | 2008 | 17 |
| 2925 | Seale et al. (2016) | K3605 | Africa | 1998 | 17 |
| 2926 | Seale et al. (2016) | K33534 | Africa | 2008 | 17 |
| 2927 | Seale et al. (2016) | K31176 | Africa | 2008 | 17 |
| 2928 | Seale et al. (2016) | K28931 | Africa | 2008 | 17 |
| 2929 | Seale et al. (2016) | K28764 | Africa | 2008 | 23 |
| 2930 | Seale et al. (2016) | K25271 | Africa | 2007 | 182 |
| 3303 | SAMEA4027127 | ERR1624805 | Europe |  | 19 |
| 3325 | SAMEA4027162 | ERR1624839 | Europe |  | 148 |
| 3335 | SAMEA4027176 | ERR1624853 | Europe |  | 9 |
| 3337 | SAMEA4027178 | ERR1624855 | Europe |  | 109 |
| 3352 | SAMEA4027196 | ERR1624872 | Europe |  | 41 |
| 3372 | SAMEA4027220 | ERR1624896 | Europe |  | 328 |
| 3382 | SAMEA4027230 | ERR1624906 | Europe |  | 109 |
| 3398 | SAMEA4027248 | ERR1624923 | Europe |  | 164 |
| 3452 | SAMEA4027312 | ERR1624986 | Europe |  | 547 |
| 3453 | SAMEA4027313 | ERR1624987 | Europe |  | 9 |
| 3454 | SAMEA4027314 | ERR1624988 | Europe |  |  |
| 3462 | SAMEA4027327 | ERR1625000 | Europe |  | 6 |
| 3475 | SAMEA4027341 | ERR1625014 | Europe |  | 107 |
| 3480 | SAMEA4027347 | ERR1625020 | Europe |  | 6 |
| 3497 | SAMEA4027370 | ERR1625043 | Europe |  | 550 |
| 3499 | SAMEA4027373 | ERR1625046 | Europe |  | 6 |
| 3505 | SAMEA4027380 | ERR1625053 | Europe |  | 148 |
| 3508 | SAMEA4027383 | ERR1625056 | Europe |  | 148 |
| 3524 | SAMEA4027403 | ERR1625076 | Europe |  | 26 |
| 3542 | SAMEA4027428 | ERR1625101 | Europe |  | 167 |
| 3559 | SAMEA4027451 | ERR1625124 | Europe |  | 933 |
| 3588 | SAMEA4027486 | ERR1625159 | Europe |  | 4 |
| 3601 | SAMEA4027503 | ERR1625176 | Europe |  | 110 |
| 3607 | SAMEA4027511 | ERR1625184 | Europe |  | 291 |
| 3617 | SAMEA4027525 | ERR1625198 | Europe |  | 148 |
| 3635 | SAMEA4027546 | ERR1625219 | Europe |  | 536 |
| 3652 | SAMEA4027563 | ERR1625236 | Europe |  | 144 |
| 3659 | SAMEA4027572 | ERR1625245 | Europe |  | 2 |
| 3663 | SAMEA4027576 | ERR1625249 | Europe |  | 110 |
| 3683 | SAMEA4027599 | ERR1625272 | Europe |  | 22 |
| 3685 | SAMEA4027602 | ERR1625275 | Europe |  | 10 |
| 3690 | SAMEA4027609 | ERR1625282 | Europe |  | 459 |
| 3694 | SAMEA4027614 | ERR1625287 | Europe |  | 328 |
| 3703 | SAMEA4027624 | ERR1625297 | Europe |  | 148 |
| 3705 | SAMEA4027626 | ERR1625299 | Europe |  | 6 |
| 3721 | SAMEA4027644 | ERR1625317 | Europe |  | 18 |
| 3735 | SAMEA4027663 | ERR1625336 | Europe |  | 751 |
| 3749 | SAMEA4027690 | ERR1625363 | Europe |  | 1 |
| 3758 | SAMEA4027705 | ERR1625378 | Europe |  | 1004 |
| 3792 | SAMEA4027749 | ERR1625422 | Europe |  | 148 |
| 3799 | SAMEA4027764 | ERR1625437 | Europe |  | 2 |
| 3821 | SAMEA4027792 | ERR1625465 | Europe |  | 1 |
| 3822 | SAMEA4027794 | ERR1625467 | Europe |  | 7 |
| 3829 | SAMEA4027802 | ERR1625475 | Europe |  | 327 |
| 3850 | SAMEA4043903 | ERR1659692 | Europe |  | 287 |
| 3854 | SAMEA4043907 | ERR1659696 | Europe |  | 148 |
| 3857 | SAMEA4043910 | ERR1659699 | Europe |  | 144 |
| 3861 | SAMEA4043915 | ERR1659704 | Europe |  | 144 |
| 3884 | SAMEA4043942 | ERR1659731 | Europe |  | 144 |
| 3906 | SAMEA4043967 | ERR1659756 | Europe |  | 1 |
| 3933 | SAMEA4043998 | ERR1659787 | Europe |  | 110 |
| 3939 | SAMEA4044007 | ERR1659796 | Europe |  | 144 |
| 3940 | SAMEA4044008 | ERR1659797 | Europe |  | 88 |
| 3946 | SAMEA4044015 | ERR1659804 | Europe |  | 287 |
| 3952 | SAMEA4044022 | ERR1659811 | Europe |  | 1 |
| 3960 | SAMEA4044031 | ERR1659820 | Europe |  | 1189 |
| 3961 | SAMEA4044032 | ERR1659821 | Europe |  | 144 |
| 3970 | SAMEA4044042 | ERR1659831 | Europe |  | 22 |
| 3982 | SAMEA4044058 | ERR1659847 | Europe |  | 103 |
| 3987 | SAMEA4044063 | ERR1659852 | Europe |  | 148 |
| 3991 | SAMEA4044069 | ERR1659858 | Europe |  | 255 |
| 3996 | SAMEA4043326 | ERR1672372 | Europe |  | 144 |
| 4023 | SAMEA4043357 | ERR1672403 | Europe |  | 144 |
| 4086 | SAMEA4043435 | ERR1672481 | Europe |  | 2 |
| 4096 | SAMEA4043449 | ERR1672495 | Europe |  | 144 |
| 4115 | SAMEA4043468 | ERR1672514 | Europe |  | 1 |
| 4121 | SAMEA4043474 | ERR1672520 | Europe |  | 144 |
| 4125 | SAMEA4043478 | ERR1672524 | Europe |  | 515 |
| 4137 | SAMEA4043490 | ERR1672536 | Europe |  | 645 |
| 4139 | SAMEA4043493 | ERR1672539 | Europe |  | 4 |
| 4142 | SAMEA4043496 | ERR1672542 | Europe |  | 1 |
| 4173 | SAMEA4043535 | ERR1672581 | Europe |  | 1 |
| 4194 | SAMEA4043559 | ERR1672605 | Europe |  | 19 |
| 4205 | SAMEA4043573 | ERR1672619 | Europe |  | 167 |
| 4208 | SAMEA4043577 | ERR1672623 | Europe |  | 233 |
| 4213 | SAMEA4043582 | ERR1672628 | Europe |  | 144 |
| 4237 | SAMEA4043609 | ERR1672655 | Europe |  | 144 |
| 4243 | SAMEA4043616 | ERR1672662 | Europe |  | 148 |
| 4244 | SAMEA4043617 | ERR1672663 | Europe |  | 291 |
| 4249 | SAMEA4043623 | ERR1672669 | Europe |  | 22 |
| 4255 | SAMEA4043629 | ERR1672675 | Europe |  | 280 |
| 4275 | SAMEA4043653 | ERR1672699 | Europe |  | 4 |
| 4282 | SAMEA4043664 | ERR1672710 | Europe |  | 22 |
| 4288 | SAMEA4043672 | ERR1672718 | Europe |  | 148 |
| 4294 | SAMEA4043678 | ERR1672724 | Europe |  | 148 |
| 4296 | SAMEA4043680 | ERR1672726 | Europe |  | 26 |
| 4298 | SAMEA4043682 | ERR1672728 | Europe |  | 110 |
| 4299 | SAMEA4043683 | ERR1672729 | Europe |  | 515 |
| 4310 | SAMEA4043695 | ERR1672741 | Europe |  | 147 |
| 4315 | SAMEA4043701 | ERR1672747 | Europe |  | 26 |
| 4338 | SAMEA4043732 | ERR1672778 | Europe |  | 144 |
| 4340 | SAMEA4043734 | ERR1672780 | Europe |  | 4 |
| 4343 | SAMEA4043737 | ERR1672783 | Europe |  | 4 |
| 4344 | SAMEA4043738 | ERR1672784 | Europe |  | 144 |
| 4347 | SAMEA4043742 | ERR1672788 | Europe |  | 4 |
| 4353 | SAMEA4043749 | ERR1672795 | Europe |  | 110 |
| 4379 | SAMEA4043782 | ERR1672828 | Europe |  | 153 |
| 4381 | SAMEA4043784 | ERR1672830 | Europe |  | 498 |
| 4385 | SAMEA4043789 | ERR1672835 | Europe |  | 147 |
| 4386 | SAMEA4043790 | ERR1672836 | Europe |  | 144 |
| 4388 | SAMEA4043793 | ERR1672839 | Europe |  | 110 |
| 4411 | SAMEA4043819 | ERR1672865 | Europe |  | 144 |
| 4417 | SAMEA4043825 | ERR1672871 | Europe |  | 109 |
| 4423 | SAMEA4043831 | ERR1672877 | Europe |  | 144 |
| 4424 | SAMEA4043833 | ERR1672879 | Europe |  | 144 |
| 4429 | SAMEA4043839 | ERR1672885 | Europe |  | 144 |
| 4432 | SAMEA4043842 | ERR1672888 | Europe |  | 751 |
| 4437 | SAMEA4043850 | ERR1672896 | Europe |  | 148 |
| 4453 | SAMEA4043870 | ERR1672916 | Europe |  | 95 |
| 4462 | SAMEA4043880 | ERR1672926 | Europe |  | 335 |
| 4466 | SAMEA4043884 | ERR1672930 | Europe |  | 335 |
| 4645 | [GCA_003160735.1](https://www.ebi.ac.uk/ena/data/view/GCA_003160735.1) | SBVN | Asia | 2016 | 7 |
| 4646 | [GCA_003160745.1](https://www.ebi.ac.uk/ena/data/view/GCA_003160745.1) | 3896VN | Asia | 2016 | 7 |
| 4666 | Sender: Pattanapon Kayansamruaj | JP9 | Asia | 2012 | 7 |
| 4667 | Sender: Pattanapon Kayansamruaj | JP17 | Asia | 2012 | 283 |
| 4668 | [GCA_001729925.1](https://www.ebi.ac.uk/ena/data/view/GCA_001729925.1) | WC1535 | Asia | 2015 | 7 |
| 4693 | Sender: Pattanapon Kayansamruaj | CBI142 | Asia | 2013 | 283 |
| 4694 | [GCA_002930695.1](https://www.ebi.ac.uk/ena/data/view/GCA_002930695.1) | UTI114 | Asia | 2013 | 283 |
| 4695 | [GCA_002937095.1](https://www.ebi.ac.uk/ena/data/view/GCA_002937095.1) | PPM3 | Asia | 2015 | 283 |
| 4696 | [GCA_001584745.1](https://www.ebi.ac.uk/ena/data/view/GCA_001584745.1) | PR10 | Asia | 2016 | 283 |
| 4697 | [GCA_002930685.1](https://www.ebi.ac.uk/ena/data/view/GCA_002930685.1) | UBN6 | Asia | 2012 | 283 |
